# Integrated behavioral and metabolically flexible responses of wild orangutans to ecologically driven dietary variation

**DOI:** 10.1126/sciadv.adv7613

**Published:** 2025-08-27

**Authors:** Erin R. Vogel, Shauhin E. Alavi, Malcolm Watford, Rebecca S.A. Brittain, Brooke E. Crowley, Daniel J. Naumenko, William D. Aguado, Timothy D. Bransford, Astri Zulfa, Alysse Moldawer, Wartika Rosa Farida, Maria A. van Noordwijk, Tatang Mitra Setia, Sri Suci Utami Atmoko, Jessica M. Rothman, David Raubenheimer

**Affiliations:** ^1^ Department of Anthropology, Rutgers, The State University of New Jersey, New Brunswick, NJ, USA.; ^2^ Center for Human Evolutionary Studies, Rutgers, The State University of New Jersey, New Brunswick, NJUSA.; ^3^ Department of Ecology of Animal Societies, Max Planck Institute of Animal Behavior, Konstanz, Germany.; ^4^ Centre for the Advanced Study of Collective Behaviour, University of Konstanz, Konstanz, Germany.; ^5^ Department of Nutritional Sciences, Rutgers, The State University of New Jersey, New Brunswick, NJ USA.; ^6^ Department of Anthropology, Yale University, New Haven, CT, USA.; ^7^ Institute of Public Health, Jagiellonian University, Krakow, Poland.; ^8^ Department of Geosciences and Department of Anthropology, University of Cincinnati, Cincinnati, OH, USA.; ^9^ Department of Anthropology, University of Colorado Boulder, Boulder, CO, USA.; ^10^ Institute of Behavioral Science, University of Colorado Boulder, Boulder, CO, USA.; ^11^ Animal Studies Discipline, Eckerd College, St. Petersburg, FL, USA.; ^12^ Department of Biology and Magister Biology, Faculty of Biology and Agriculture, Universitas Nasional, Jakarta, Indonesia.; ^13^ Research Center for Applied Zoology, National Research and Innovation Agency (BRIN), Cibinong-Bogor, Indonesia.; ^14^ Department of Evolutionary Anthropology, University of Zurich, Zurich, Switzerland.; ^15^ Department of Anthropology, Hunter College of the City University of New York, New York, NY, USA.; ^16^ Charles Perkins Centre and School of Life and Environmental Sciences, The University of Sydney, Sydney, Australia.

## Abstract

Diet and nutrition are critical factors influencing energetics and health. Laboratory studies show that organisms adjust to changes in nutrient intake through flexible metabolic responses such as fuel switching. While the physiological effects of nutrient balance in humans have been studied, data from closely related species living in nature are lacking. We integrate macronutrient regulation and metabolic flexibility to elucidate how wild orangutans (*Pongo pygmaeus wurmbii*) are buffered against natural fluctuations in nutritional resources. We found that these orangutans regulate protein and regularly switch between exogenous and endogenous nutritional substrates as preferred food resource availability declines. When total caloric, lipid, and carbohydrate intake declined during episodes of fruit scarcity, orangutans drew on fat and endogenous amino acids for energy. This strategy is beneficial only in the context of alternating periods of fruit scarcity and abundance. We discuss our findings in relation to the current global obesity pandemic, which has arisen with transitions in human diets toward low-cost, energy-dense, protein-dilute foods.

## Teaser

Orangutans have evolved behavioral and metabolic flexibility to cope with a feast-and-famine ecology.

## INTRODUCTION

An animal’s demand for nutrients and energy varies over time, and animals must meet these demands while experiencing fluctuations in the availability of food resources (*1*). Behavioral and physiological adaptations to these fluctuations can influence fitness and population dynamics and have also been implicated in maladaptation in circumstances where species are chronically exposed to altered environments, as is the case with the modern pandemic of human obesity and metabolic disease (*1*–*3*). Recognizing an animal’s flexibility to nutrient supply and demand is thus valuable for understanding ecological and evolutionary processes, which in turn can be applied to conservation strategies for a species (*4*). While behavioral (foraging) flexibility has long been a topic of interest, the concept of “metabolic flexibility,” which describes the ability of an organism to adaptively switch between different fuel substrates (e.g., glucose, glycogen, fatty acids, or amino acids) with changes in nutrient availability, has emerged as an important framework for understanding the adaptive capacity of physiological systems to environmental variation (*3*). This flexibility can have profound effects on health, disease, and life history responses such as life span and reproductive output (*3*). Determining how species, including humans, interact with natural resource variation in the ecological context where they evolved is crucial for understanding these responses.

Laboratory experimental research has shown that organisms ranging from acellular slime molds to insects to mammals regulate their intake of particular macronutrients to meet their physiological requirements, exhibiting metabolic flexibility to mitigate health impacts when faced with perturbations to their food environment (*5*). The multidimensional nutritional geometry framework has enabled a higher resolution characterization of interactions between nutrient intake and physiological response (*5*). This regulation of nutrients can have profound effects on immune function [e.g., (*6*)], fat storage and heat production (*7*), obesity (*8*), aging and life span (*9*), glucose tolerance (*10*), and blood pressure (*11*). It has become clear that specific combinations of different nutrients are required for maintenance, growth, and reproduction, and these dynamic nutritional needs are dependent on the changing physiological condition of the forager in response to variable resource availability (*12*–*15*). In summary, these studies have demonstrated that in a laboratory setting, the integration of both flexible diet selection and metabolism ameliorates the impacts of diet variability, but chronic dietary imbalance can nonetheless lead to impaired fitness and health.

We know very little about how organisms integrate behavioral and physiological responses to tolerate variability in nutritional balance in natural settings. Several studies have shown that wild primates adopt a variety of strategies in the face of natural variation in food type and nutrient availability [reviewed in (*16*, *17*)]. Mountain gorillas (*Gorilla beringei*), a more folivorous species, maintain absolute fat and carbohydrate intake across the annual cycle, while protein intake varies with the availability of fruits [i.e., “non-protein energy (NPe) prioritization”] (*18*). In contrast, more frugivorous spider monkeys (*Ateles chamek*) (*19*), blue monkeys (*Cercopithecus mitus*) (*20*), ruffed lemurs (*Varieca variegata*) (*21*), and chimpanzees (*Pan troglodytes schweinfurthii*) (*22*) maintain relatively constant protein intake, while non-protein energy intake varies substantially (i.e., “protein prioritization”). Omnivorous rhesus macaques (*Macaca mulatta*) prioritize neither protein nor non-protein energy but regulate their diet to maintain constant caloric intake (*23*). Last, folivorous-frugivorous sifakas (*Propithecus diadema*) maintain a relatively constant ratio of non-protein energy to protein intake while varying both the total amount of protein and non-protein energy intake depending on seasonal variation (*24*).

However, very little is known about how primates’ macronutrient balancing strategies interact with physiology to buffer fitness in the face of ecologically driven resource variability. Integrating macronutrient regulation and metabolic flexibility can help elucidate how animals may be buffered against resource instability in the wild. Bornean orangutans (*Pongo pygmaeus* spp.) are a promising model species for examining this. They experience extreme and unpredictable variation in fruit availability relative to Neotropical or African primates (*25*), meeting their nutritional needs from multiple other food types (*26*), and exhibit many of the distinguishing characteristics that define generalist herbivores (*27*, *28*). For example, while orangutans prefer fruit (*29*), within each single population they are known to feed on a large variety of foods, including fruits, flowers, insects, inner bark, and leaves that exhibit high overlap in their nutritional compositions (*26*, *30*). During low fruit periods, several populations of orangutans have been shown to rely on fallback foods, such as leaves and inner cambium, that tend to have lower energy density compared to fruit (*26*, *31*, *32*). These dietary shifts result in striking variation in caloric intake (*31*–*33*), leading to negative energy status, as evidenced by increased fat catabolism (*32*, *34*). At the Tuanan research area in Central Kalimantan, Indonesia, low fruit periods were also associated with reduced muscle mass in orangutans (*35*), yet little is known about how fat and muscle catabolism are related to nutrient deficits across these unpredictable food landscapes. Orangutans have the slowest life-history and highest survivorship to reproductive age of any nonhuman primate (*36*) and have a suite of physiological (e.g., hypometabolism) (*37*), genetic (e.g., high gene copy number coding for protein-digesting enzymes) (*38*), and morphological (e.g., thickly enameled teeth and robust jaws) (*39*, *40*) adaptations that are hypothesized to help them survive lean periods. Pontzer and colleagues (*37*) proposed that the unique hypometabolism found in orangutans is an adaptation to extended episodes of caloric deficits. In support of this, Mattle-Greminger and colleagues (*41*) found evidence for positive selection in Bornean orangutans for genes involved in cardiac activity, lipid metabolism, and energy storage. In addition, the slow life history and semi-solitary lifestyle of orangutans have been associated with periodic energy scarcity (*42*–*44*).

We investigated how wild orangutans (*P. pygmaeus wurmbii*) in the Tuanan research area cope with ecologically driven dietary variation by examining their daily nutritional intake and physiological responses to stochastic episodes of fruit scarcity in this tropical peatland habitat (*33*). We combined full-day focal animal feeding data with nutritional analyses of foods to examine how ecologically driven variation in macronutrient intake is related to urinary biological markers of energetic condition (table S1). An animal’s energetic condition is based on both energetic input and energy expenditure. When energetic input exceeds expenditure, excess energy is stored in adipose tissue. However, when energy expenditure exceeds intake, animals can transition to increased endogenous fat and muscle catabolism to compensate for energetic shortfalls (*45*, *46*).

Previous studies on wild orangutans (*34*, *47*) and other primates (*48*–*53*) have demonstrated that energy balance can be monitored using a combination of urinary C-peptide levels, an index of insulin production (*54*), and urinary ketone bodies, a by-product of fat catabolism [see review by (*48*)]. Likewise, urinary urea concentration and nitrogen isotope (δ^15^N) values can be used to assess the use of body protein for energy (*55*, *56*). During periods of low-caloric or carbohydrate intake, glucose formed from non-carbohydrate precursors via gluconeogenesis primarily fulfills the brain’s obligatory requirement for glucose oxidation (*45*, *46*). Gluconeogenic precursors include lactate, glycerol from triglycerides, amino acids, and possibly propionate from microbial fermentation of soluble dietary fiber (*46*, *57*). At times of energetic deficit, glycerol and amino acids derived from the diet and endogenous protein breakdown are major substrates for gluconeogenesis, releasing free glucose or storing it as hepatic glycogen. Liberated nitrogen is used for urea synthesis, and thus, gluconeogenesis from amino acids and urea synthesis may be considered as a single pathway (*46*). We can distinguish gluconeogenesis from dietary or endogenous amino acids by evaluating the relationships between protein intake, urea concentration, and δ^15^N values (see table S1) (*55*). The breakdown of dietary protein will increase urea excretion, but at times of low-calorie intake, this relationship may be altered by endogenous protein breakdown. The breakdown of endogenous amino acids will increase urinary δ^15^N, indicating increased proteolysis (i.e., tissue catabolism).

We used the nutritional geometry framework (*14*) to examine how the pattern of orangutan macronutrient intake corresponds with ecological variability in fruit availability at Tuanan. We hypothesized that orangutans conform to a model of protein prioritization previously observed in other frugivorous primates (H1; Table 1) and tested two predictions. First, we predicted that fruit availability (i.e., the percentage of fruiting trees) dictates the variability in dietary non-protein energy (NPe) intake by individuals [i.e., the sum of lipid, fiber, and nonstructural carbohydrates (kcal)], as fruits tend to be the food source highest in NPe [P1]. Second, because protein intake varies less than NPe intake in this population (*30*), we predicted that fruit availability influences the ratio of daily NPe to protein consumed [P2].

**Table 1.** Summary of the hypotheses, predictions, and results related to orangutan macronutrient intakes and energy condition (also see table S1).

Hypothesis	Prediction	Result summary
1) The availability of fruit drives macronutrient intake (g/day)	1) Non-protein energy (NPe) will vary with habitat fruit availability (FAI), but protein (P) intake will remain relatively constant. The power function (*L*) of total daily caloric intake and percentage of protein would be equal to −1 in the case of complete protein prioritization.	NPe intake varied with FAI, but protein intake did not.
		Protein prioritization was strong but not complete.
	2) FAI will influence the ratio of non-protein energy to Protein (NPe:P).	NPe:P increased with FAI, until FAI reached the average FAI for the habitat.
2) Orangutans use flexible metabolic responses via the gluconeogenic pathway to buffer energetic shortfalls	3) If orangutans primarily use lipid reserves for gluconeogenesis to offset energy deficits when fruit is scarce, then we predict fruit scarcity and consequent reduced energy intake will be associated with a) greater urinary ketone body presence and b) lower urinary C-peptide of insulin.	FAI was not related to ketone body presence or C-peptide of insulin.
		Total caloric, NPe, and carbohydrate intakes were not related to ketone body presence or C-peptide of insulin.
	4) If orangutans primarily use dietary (exogenous) protein as the source of gluconeogenic amino acids, then we predict that a) increased urea concentration will be associated with low FAI, b) urea concentration will increase with increased protein intake, and c) urea concentration will not vary with total caloric intake and carbohydrate intake.	Urea concentration increased when fruit was scarce.
		Urea concentration was not related to protein intake.
		Increased urea was associated with lower intakes of total calories, NPe, lipid, and carbohydrates.
	5) If orangutans primarily use endogenous protein as the source of gluconeogenic amino acids, then we predict that a) low-fruit periods will be associated with increased urea concentration and δ^15^N values, b) increased protein intake will not be associated with increased urea concentration but could be associated with lower δ^15^N if NPe intake is high, and c) both urea concentration and δ^15^N will increase when NPe, carbohydrate, lipid, and/or caloric intake are low.	Urea concentration increased with decreasing fruit availability but did not vary with protein intake.
		δ^15^N was not related to fruit availability.
		Increased urea and δ^15^N were associated with lower total caloric and carbohydrate intakes.

We then tested the hypothesis (H2; Table 1) that as dietary generalists, wild orangutans buffer the consequences of nutritional imbalance using flexible metabolic responses to modulate how macronutrients are processed, interconverted, and used. On the basis of laboratory experiments (*58*) and known metabolic consequences of variable energy and macronutrient supply and demand in humans (*3*), we predicted that orangutans exhibit two types of physiological responses. First, we predicted that at times of fruit scarcity, when total caloric and/or non-protein energy intake is low (*30*), individuals enter a more negative energy balance. They begin to mobilize body fat reserves to release free fatty acids as fuel for most cells and provide glycerol from triglyceride for gluconeogenesis [P3]. This should result in increased ketone body presence and lower urinary C-peptide levels (Table 1 and table S1). Second, we predicted [P4 to P5] that when total caloric and/or carbohydrate intake is low, the need for gluconeogenesis will result in the catabolism of both dietary amino acids from proteinaceous foods (e.g., young leaves, insects) and potentially endogenous body protein (*59*), both of which would be indicated by increased urinary urea concentration (Table 1 and table S1). When diet is the primary source of gluconeogenic amino acids, we expect a positive correlation between urea concentration and protein intake, but no relationship between urinary nitrogen isotopes (δ^15^N) and protein intake [P4]. If, on the other hand, orangutans rely more on endogenous amino acids for glucose production (i.e., for energy and to fuel the brain) when caloric intake (and more specifically carbohydrate intake) is low (sensu *35, 55*) and absolute protein intake remains relatively constant or decreases, then we expect both urea concentration and δ^15^N values to increase [P5] (*55*, *56*).

## RESULTS

### Food availability and orangutan nutritional intake

**Fig. 1.**
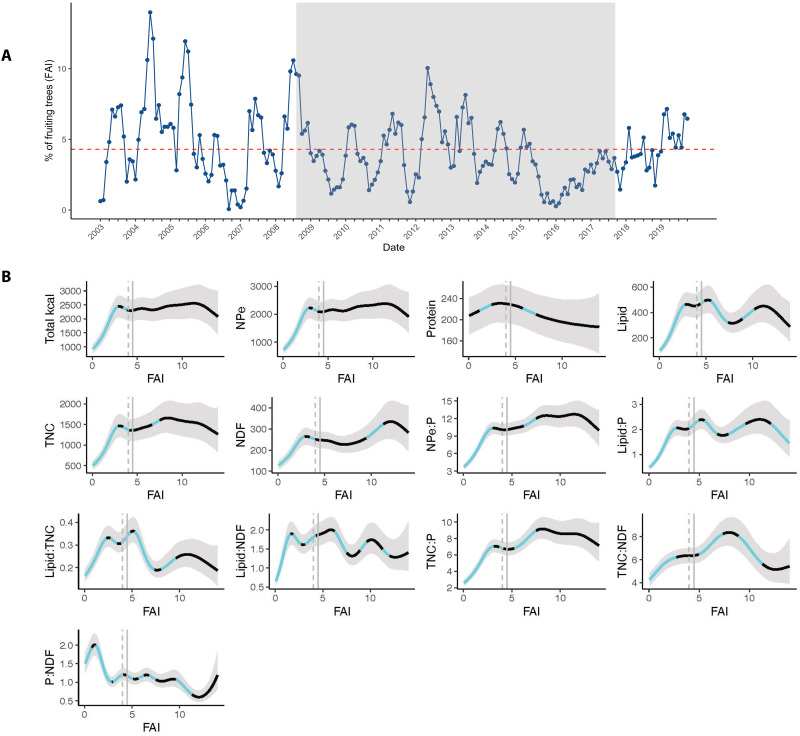
Fruit availability and macronutrient intakes (kcal/day). (**A**) Monthly percentage of fruiting trees (FAI) in the phenology plots in the Tuanan orangutan research area. The dotted red line indicates the 50% quartile used to define low- and high-fruit periods. The shaded gray area represents the majority of the urine data collection period for this study, with the exception of ketone bodies, which took place over a longer time period (see fig. S8). (**B**) Relationship between FAI and macronutrient and macronutrient ratio intakes. All macronutrient intakes represent % of dry matter in kcal. The dashed gray line represents the mode, and the solid gray line represents the mean for FAI. The light blue sections of the curve represent the periods along the fitted splines where the rate of change in the response (slope) was different from 0 (not flat) with 95% credibility. Total kcal, total daily caloric intake; NPe, non-protein energy; TNC, total nonstructural carbohydrates; NDF, neutral detergent fiber; P, protein. Created in BioRender [Erin R. Vogel (2025); https://BioRender.com/vbmy3c3].

**Fig. 2.**
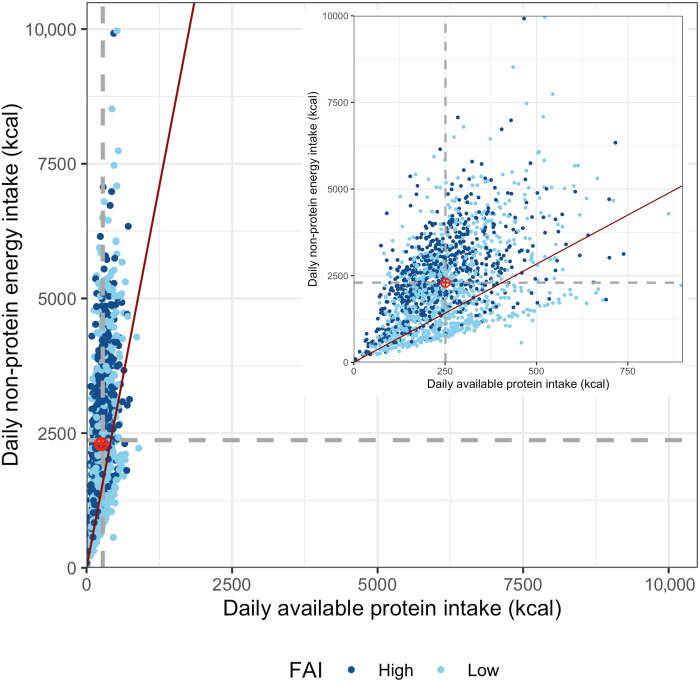
Average available protein (kcal) and non-protein energy (kcal) intakes. Averages for each continuous follow-set period (*n* = 1636) for individuals using the geometric framework in which *x* and *y* axes are equal. The dashed lines represent the overall mean intakes. The red line is the recommended NPe:P for human intake (*117*). The estimated target intake ratio (where the means intersect) for orangutans is represented by the red target. Insert is a zoomed-in version of the *x* axis to view the dispersion of high and low fruit periods. Created in BioRender [Erin R. Vogel (2025); https://BioRender.com/h84o222].

To examine the macronutrient intake strategy of Tuanan orangutans in response to ecological variability, we analyzed the relationships between fruit availability and protein and non-protein energy intake during full-day follows of individual orangutans over a 15-year period. We observed considerable variation in fruit availability (Fig. 1A and table S2). The percentage of fruiting trees per month [fruit availability index (FAI)] ranged from 0.5 to 14% during the study period (table S2), with an average of 2.44% in the low fruit periods and 6.68% in the high fruit periods. Consistent with previous research over a shorter period, which demonstrated a positive relationship between total caloric intake and FAI (*30*), intake of NPe (carbohydrates and lipids) increased with FAI (Fig. 1B). Total caloric intake also increased with the FAI (Fig. 1B and table S3), with a 19.2% increase in calories (kcal/day) between low and high fruit periods (table S2). In support of [P1], we found that the variation in total caloric intake was driven largely by variation in NPe intake, which increased by 21.7% between low and high fruit periods. In contrast, average protein (P) intake decreased only 3.9% between low and high fruit periods, on average, accounting for 10.1% of total calories consumed and not varying predictably with FAI (Fig. 1B and tables S2 and S3). Orangutan diets resulted in an average ratio of NPe to protein (NPe:P) of 9.7:1.0 (Fig. 2 and table S2). However, this ratio varied from 8.5:1 to 11.6:1 (i.e., an increase of 30.9%) between low and high fruit periods, respectively. At lower-than-average FAI, there was a strong positive response in NPe:P intake with FAI, while at higher-than-average FAI, dietary NPe:P did not vary with FAI with any certainty, providing partial support for [P2] (Fig. 1B and tables S2 and S3).

Using the relationship between total daily energy intake and percent dietary energy from protein [sensu (*60*)], we found that protein prioritization was strong but not complete. The power function of dietary percentage protein had an exponent of *L* = −0.73 (*r*^2^ = 0.39 (0.37 to 0.40) (fig. S1 and table S4) compared with an exponent of −1 for complete protein prioritization (*60*). Thus, absolute protein intake varied to some extent, but much less than absolute NPe and total energy intake varied with dietary NPe:P (*60*, *61*).

We also examined the relationships between other intakes of macronutrients [i.e., lipids, total nonstructural carbohydrates (TNC; hereafter referred to as “carbohydrates”], neutral detergent fiber (NDF; hereafter referred to as “fiber”), their ratios, and FAI. Overall, we found that absolute and relative macronutrient intakes were positively related to FAI up to the average FAI and then plateaued. This indicates that when fruit availability is low, nutritional intake is overall reduced, while at above-average FAI, the dietary strategy does not vary predictably with FAI (Fig. 1B and table S3). For example, both lipids and carbohydrates followed a similar pattern to total caloric and NPe intakes (Fig. 1B), respectively, increasing by 20.6 and 22.6% between the low and high fruit periods (table S2). However, proportional lipid and fiber intakes, which were highly nonlinear, did not vary predictably with FAI (Fig. 1B).

### Integration of physiology, fruit availability, and macronutrient intake

**Fig. 3.**
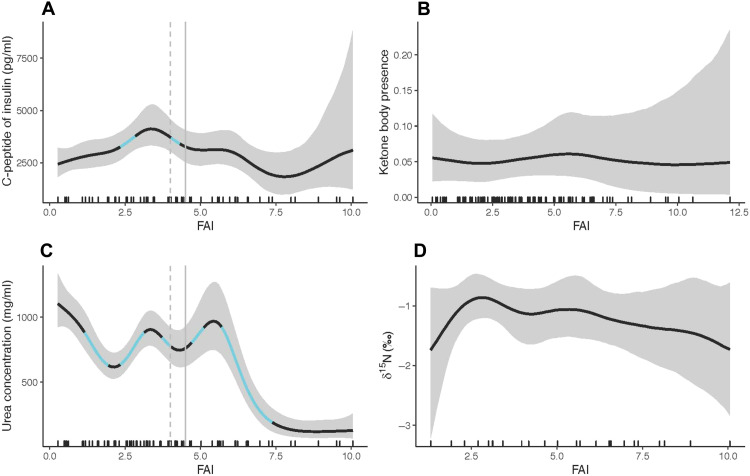
Relationship between FAI and urinary biomarkers of energetic condition. (**A**) C-peptide of insulin (pg/ml); (**B**) ketone body presence; (**C**) urea (mg/ml); (**D**) δ^15^N. For graphs with slopes different from 0 (with 95% credibility), the dashed gray lines represent the mode, and the solid gray lines represent the mean for FAI (only indicated for panels with an effect). The light blue sections of these curves represent the periods along the fitted splines where the rate of change in the response (slope) was different from 0 (i.e., not flat) with 95% credibility (see Materials and Methods). The rug plot represents the actual data distribution. Created in BioRender [Erin R. Vogel (2025); https://BioRender.com/i82p84y].

Contrary to our predictions [P3], we did not find consistent relationships between fruit availability (FAI) and most biomarkers of energetic condition (at 95% certainty) (Fig. 3 and table S5). We detected a response of C-peptide levels at a few ranges of FAI values, but the direction of the relationship was not consistent (Fig. 3A). While there were three times as many urine samples that tested positive for ketone bodies during low fruit periods compared to high fruit periods (6.6% versus 2.6%, respectively), we found no statistical evidence for a response between FAI and ketone body presence (at 95% certainty) (Fig. 3B). Thus, there was no evidence for either a positive relationship between C-peptide levels and FAI or a negative relationship between urine ketone body presence and FAI (table S5). We found a sharp decrease in urea concentration above the average FAI, but below average FAI urea concentration remained higher (Fig. 3C and table S5). This suggests that ureagenesis, and thus gluconeogenesis, declined when FAI was above average in the habitat. However, we did not find support for a relationship between FAI and δ^15^N values (Fig. 3D).

Intake of macronutrients and the ratios of macronutrients had mixed relationships with biomarkers of energetic condition. Contrary to [P3], we did not find strong support for a relationship between ketone body presence and caloric intake, macronutrient intakes, or the macronutrient intake ratios (fig. S2 and table S6). There was a suggestion of a negative relationship between the ratio of lipid to carbohydrate intake with ketone body presence, which occurred when the ratio fell above the intake mode (0.18; tables S2 and S6). However, the likelihood of observing this increase in ketone body presence was very low (ranging between 0.03 and 0.05 on a total scale of 0 to 1; fig. S2). We also observed an exceedingly small negative response in C-peptide levels for total caloric and NPe intakes, which fell above the intake means and modes (fig. S3 A and F, and tables S2 and S6). For the ratios NPe:P and TNC:P, a negative response in C-peptide levels coincided with the mean intake for these ratios; there was no response in C-peptide across the rest of the range of intakes (fig. S3, G and K, and table S6).

**Fig. 4.**
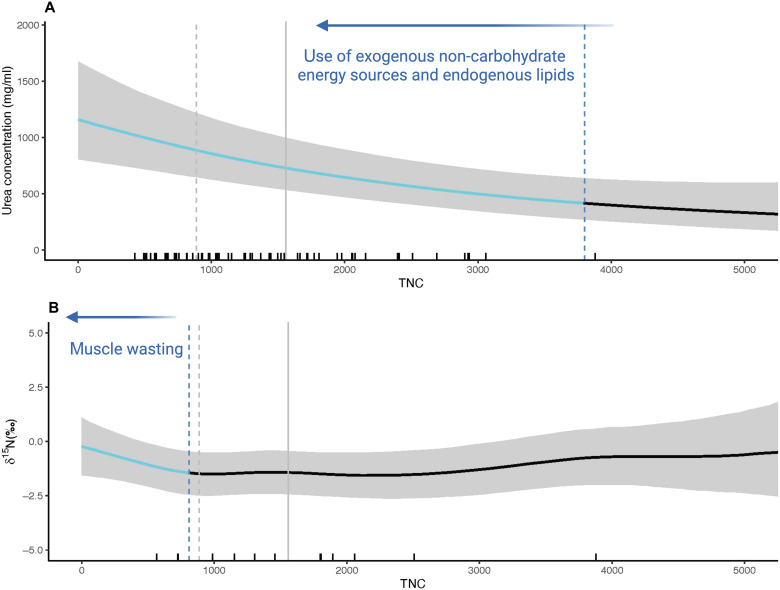
The relationship between total nonstructural carbohydrate intake and biomarkers of body protein catabolism. Relationship between total nonstructural carbohydrate intake (TNC, kcal) and (**A**) urea concentration (mg/ml) and (**B**) urinary δ^15^N values. The dashed gray line represents the mode, and the solid gray line represents the mean for TNC. Mean and mode are only indicated for panels with a response with 95% credibility. The light blue sections of the curve represent the periods along the fitted splines where the rate of change in the response (slope) was different from 0 (i.e., not flat) with 95% credibility. The blue dashed line represents the upper cutoff for the significant region of the curve. The rug plot represents the actual data distribution. Created with BioRender.com [Erin R. Vogel (2025); https://BioRender.com/cxj0y7t].

We did, however, find strong evidence that orangutans have metabolic flexibility and rely on amino acid and glycerol gluconeogenesis to compensate for low carbohydrate intake (figs. S4 and S5 and tables S6 and S5). Urinary urea concentration decreased as NPe:P increased (fig. S4G). There was no detectable relationship between urea concentration and protein intake (fig S4B), but there was a negative relationship with some NPe components (e.g., lipids, carbohydrates) and total caloric intake (Fig. 4A and fig. S4, A, C, and D). Urea concentration did not vary individually with absolute fiber, but we observed a positive relationship with the ratio of protein to fiber intake (fig. S4, B, E, and M, and table S6). We also found that δ^15^N values increased when total caloric intake, carbohydrate, and NPe intakes were far below average intakes, but δ^15^N values were not related to protein intake (fig. S5, A, B, D, and F, and tables S2 and S5). Thus, contrary to P4, the observed increase in urea concentration with lower caloric, NPe, and NPe:P intake was not due to an increase in dietary protein intake. Instead, and supporting [P5], our results suggest that increased urinary urea is linked more to the use of body amino acids for gluconeogenesis, showing that orangutans catabolize endogenous body protein when total nonstructural carbohydrate intake is low. Overall, variation in both urea and δ^15^N was largely driven by carbohydrate intake (Fig. 4, A and B). These data provide strong evidence that wild orangutans regularly enter a state of tissue catabolism for fuel when nonstructural carbohydrate intake is greatly reduced.

## DISCUSSION

We examined how multidimensional macronutrient regulation interacts with metabolic responses to ecologically imposed dietary variation in an ape that is adapted to stochastic periods of energetic and nutritional stress. We provide the first evidence for a clear link between ecological variability, daily macronutrient intake, and energetic status in wild Bornean orangutans living in a peat swamp habitat. We found that when the availability of fruit was below average for the habitat, macronutrient intake markedly declined and was associated with a reliance on endogenous lipids and amino acids. The reliance on body amino acids and glycerol for gluconeogenesis when non-protein energy, specifically carbohydrate, intake is low demonstrates that the wild orangutans in this study exhibit metabolic flexibility, adaptively using endogenous lipids for most tissues and endogenous amino acids to provide glucose for the brain, similar to other mammals, including humans (*45*).

As we predicted [P1], orangutans maintained a more constant intake of absolute protein compared to non-protein energy intake, the same strategy of protein prioritization shown by other more frugivorous non-human primates (*19*–*22*) and humans (*60*, *62*). The ratio of non-protein energy to protein intake (NPe:P) for Tuanan orangutans in this study (9.7:1) is similar to the intake ratio observed in other frugivorous primates (*16*, *63*) and at the lower end of human diets (*64*). Previous research suggests that Tuanan orangutans actively target this ratio, since over 18 months, the observed ratio of non-protein energy to protein intake was significantly smaller than expected based on habitat nutrient availability (*65*). DiGiorgio and colleagues (*66*) also found that orangutans in a masting forest do not use a simple energy maximization strategy and leave fruit trees to consume leaves from species without fruit, even when edible fruits remain. Active macronutrient balancing was also shown in an experimental study of humans, who targeted 14.7% of energy from protein (*67*). Thus, several studies in different ecological contexts have shown that protein prioritization is a salient feature of more frugivorous primates and humans.

However, there were also several unexpected results in our analysis of metabolic responses to daily nutrient intake. Contrary to [P2], we did not observe an increase in urinary ketone bodies or a decrease in C-peptide levels with decreased caloric, carbohydrate, or non-protein energy intake at Tuanan (table S6). Although 9.2% of the urine samples in our study were positive for ketone bodies, we found that ketosis in Tuanan orangutans occurs throughout both low- and high-fruit periods. The percentage of positive ketone samples was 6.6% in the low-fruit period compared to 2.6% in the high-fruit period, although this difference did not reach statistical significance. There are several possible explanations for the lack of predicted results between ketosis and daily macronutrient intakes. First, the presence of ketone bodies during high-fruit periods when caloric intake was higher may be a result of the confounding effect of social interactions during the high-fruit period (*43*). Tuanan female orangutans socialize more with males during high-fruit periods. However, prolonged social interactions are costly and are associated with decreased feeding, increased traveling, and increased fecal glucocorticoids, indicating energetic stress (*44*). Second, even during the high-fruit period, there were 165 days (of 2047) when caloric intake was below 1000 kcal/day. Third, while the Chemstrips we used to measure ketone body presence have been validated for use with orangutans (*68*), they only detect acetoacetate and not β-hydroxybutyrate, the predominant ketone body (*69*). In our previous validation, a negative reading was not distinguished from small quantities of ketones; thus, mild or moderate levels of ketosis may not be detected (*68*). Similarly, a recent study on humans found that urine dipsticks have low sensitivity and were unable to detect mild instances of ketosis based on blood β-hydroxybutyrate levels (*70*). In humans on low-carbohydrate ketogenic diets (<5 to 10% of kcal consisting of carbohydrates), it takes at least 3 to 4 days of consistently low carbohydrate intake for an individual to enter a ketotic state detectable by urine detection strips. We used nutritional intakes from the day before the sample was collected. For all but 1 day, the Tuanan orangutans had greater than 20% of their caloric intake from nonstructural carbohydrates, and even during the low fruit period, the average nonstructural carbohydrate intake was about 59% of daily calories. Thus, perhaps a carbohydrate-rich diet (fig. S6 and table S7), combined with consistent protein and low-fat intake, kept the orangutans from having detectable levels of ketosis, even during low-fruit periods. In support of this, we found that individuals go in and out of detectable ketosis daily across individual sampling periods with three or more consecutive follow days with matching urine data (fig. S7). Although most urine samples were first morning voids (after an overnight fast), levels of ketosis may have been too low to be detectable using these Chemstrips. Future studies would benefit from using a more quantitative assay when examining ketosis and nutrition.

A lack of relationship between urinary C-peptide levels and fruit availability in this population may be explained by peatland ecology and our estimates of monthly fruit availability. Sherry and Ellison (*47*) showed that urinary C-peptide levels were higher in a small number of wild orangutans (*n* = 3) when fruit availability was high, and Emery Thompson and Knott (*34*) found significant positive relationships between average monthly urinary C-peptide levels and both monthly ripe fruit availability and average monthly energy intake at the same site in West Kalimantan. Both studies took place in a masting forest with one very high-fruit period followed by an extended period of fruit scarcity. In contrast, our study examined the integration of biomarkers of energetic condition with matched daily macronutrient intake over a 7-year period characterized by six cycles of high- and low-fruit periods in a peatland forest that does not experience extreme masting (Fig. 1A) (*30*, *33*). In addition, our index of fruit availability {i.e., the monthly percentage of fruiting trees in the phenology plots; [sensu (*71*)]} does not consider the biomass of available fruit in each tree. Future studies should compare the use of alternative measures for estimating habitat fruit and/or energy availability.

Other studies have also had mixed results in evaluating relationships between urinary C-peptide levels and estimated nutritional intakes. One study on mountain gorillas (*Gorilla beringei beringei*) found an increase in C-peptide levels during periods of heightened bamboo consumption (*72*), a food that has high protein relative to fats and carbohydrates (*73*). Similarly, an increase in C-peptide was observed for a wild population of provisioned chacma baboons (*Papio ursinus*) (*74*). However, other studies on wild primates have failed to find the positive relationship between C-peptide levels and energy intake that one might expect (*53*). The lack of a strong relationship between C-peptide levels and energy intake, as in our study, might be because C-peptides reflect energy balance, not just energy intake (*48*, *49*). Compensatory behavioral strategies to reduce energy expenditure can affect C-peptide levels. For example, Tuanan orangutans spend less time feeding and moving, have shorter active periods, socialize less, and have smaller day ranges when fruit is scarce (*30*, *44*, *75*). Thus, they can reduce energy expenditure to compensate for energetic shortfalls up to a point. For orangutans in low-fruit periods and mountain gorillas feeding on high-protein bamboo, it is also possible that urinary C-peptide levels do not decrease with reduced non-protein energy intake because of an insulinotropic effect (i.e., stimulating insulin secretion) of the high dietary protein and/or muscle catabolism, as is observed in humans (*76*).

We found evidence of declining energetic condition and muscle catabolism during low-fruit periods, although protein intake was maintained (255 kcal protein/day on average). This was demonstrated by an increase in urinary urea concentration as carbohydrate, total caloric intake, NPe, and NPe:P intakes all decreased. Most notable was that a decrease in carbohydrate intake was associated with an increase in both urea concentration (below ~3800 kcal/day) and urinary δ^15^N levels (below ~800 kcal/day) (Fig. 4, A and B). This supports our prediction [P5] that when caloric and carbohydrate intakes are low, orangutans catabolize endogenous amino acids via gluconeogenesis (*55*, *56*, *59*, *77*). It is possible that the observed increase in urinary δ^15^N as carbohydrate intake declined was due to isotopic differences between fallback foods (primarily leaves and bark) and fruit (*30*). However, we think that this is unlikely, as it would require that leaves and bark have higher δ^15^N values than fruit. We do not have δ^15^N values for the plants consumed at Tuanan, but in other settings, leaves and bark are not more ^15^N-enriched than fruit (*78*–*82*). Instead, we interpret the increase in urinary δ^15^N as the result of orangutans catabolizing endogenous amino acids for gluconeogenesis, which is consistent with a recent finding that Tuanan orangutans lose lean body mass during low fruit periods (*35*). Despite the lack of detectable urinary ketones, these data collectively suggest that orangutans draw on endogenous fats and endogenous amino acids and glycerol for glucose production when carbohydrate intake is low, specifically during the low fruit periods (*45*).

**Fig. 5.**
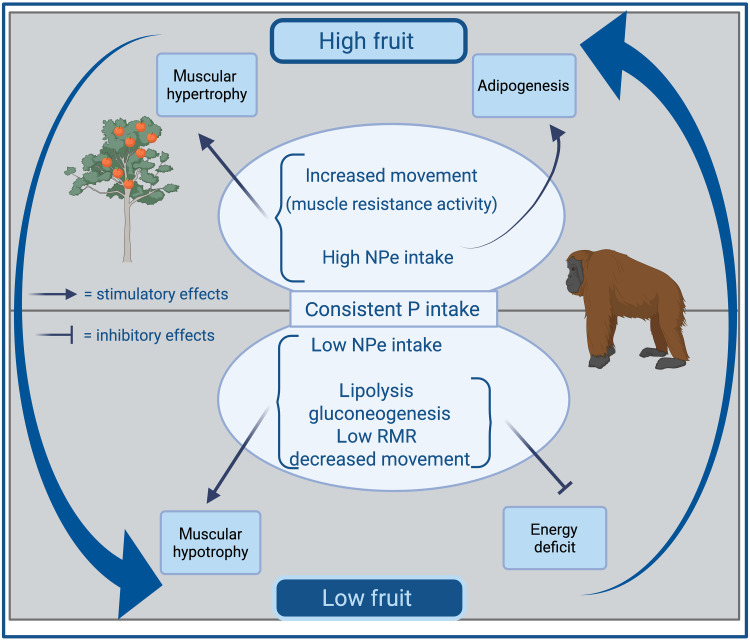
A model for understanding the behavioral and physiological adaptations of wild orangutans to an unpredictable resource base in wild orangutans. NPe, non-protein energy; RMR, resting metabolic rate. Created in BioRender [Erin R. Vogel (2025); https://BioRender.com/r5r244o].

Our results highlight the importance of the unpredictable “Feast-and-Famine” ecology experienced by orangutans in their natural habitats (*83*–*85*) and how wild orangutans have adapted to resource variation through the integration of flexible feeding behavior and metabolic activity (Fig. 5). Ecologically driven fluctuations in the availability of energy-rich fruits are associated with highly variable intakes of carbohydrate, fat, and total energy. Yet, daily protein intakes are maintained relatively constant by consuming proteinaceous foods in their diets (e.g., pith, young and mature leaves; fig. S6 and table S7), even when fruit is abundant (*30*, *55*). Fluctuations in nutrient availability affect energetic status but are partly compensated by specific adaptations for efficient energy acquisition and reduced expenditure demonstrated in previous studies (*37*, *38*). When fruit availability is low and carbohydrate intake is reduced, orangutans draw on fat reserves for energy and use endogenous protein for gluconeogenesis to support the brain. Consequently, both muscle and fat need to be rebuilt during high-fruit periods, when total energy intake is high. This may explain why we observe relatively consistent protein intake. Yet, during high-fruit periods, we do not see evidence of excess protein intake in the form of higher urea concentration, potentially because orangutans continue to use dietary amino acids to rebuild lost muscle mass. Orangutans also exhibit behavioral flexibility during the low-fruit periods when they experience extended periods of reduced caloric intake by reducing physical activity (*30*, *75*). Another peculiarity of orangutan nutritional physiology might explain how this challenge of episodes of restricted nutritional intake is met. Great apes have more pepsinogen-A genes that code for protein-digesting enzymes than other mammalian taxa, which has been hypothesized to be advantageous for the efficient digestion of protein from herbaceous foods in their diet (*38*). Orangutans have the greatest number, resulting in the highest levels of pepsinogens in their stomachs (*38*). This is possibly related to orangutans’ need to efficiently digest protein to meet the requirements for muscular hypertrophy during periods of high-fruit intake.

An important outstanding question is what are the ecological circumstances that drive the evolution of this metabolic flexibility? Southeast Asian forests and their fruiting patterns, driven by unpredictable El Niño-Southern Oscillation (ENSO) events (*86*), have shaped the unique adaptations observed in orangutans. Even in peatland habitats such as Tuanan that do not experience fruit masting, ENSO events influence the timing and magnitude of fruiting and flowering (*87*). Similar ENSO patterns have been hypothesized to have influenced population structure, migration patterns, resource availability, and consequent dietary adaptations in hominins over the past 620,000 years across Africa (*88*). It has been suggested that hominin diets have changed markedly over evolutionary time (*89*), and certain common metabolic disorders in humans, such as type 2 diabetes, are likely a consequence of the much older history of natural selection for metabolic flexibility in the face of limited and unpredictable food availability (*90*). Even over the past 12,000 years with the rise of agriculture and the industrial period, there has been a major shift in human diets, resulting in a reduction in dietary diversity, and a decrease in dietary protein relative to carbohydrates and fat (*91*, *92*). Concomitantly, there have been changes in human metabolism that further increase exposure of human physiological systems to carbohydrate and fat (*91*, *92*). For example, humans are enriched compared to monkeys and apes in genes that select for carbohydrate and lipid metabolism (*93*). A specific instance of this is the high copy numbers of starch-digesting AMY1 genes in humans (*94*), also found in domesticated species such as dogs, mice, and pigs (*95*), which are associated with metabolic state and susceptibility to obesity (*96*, *97*). Similar to what we observed in orangutans, humans in starvation have also been shown to reduce physical activity and experience overall loss of lean and fat tissue (*98*).

In the context of human health, wild orangutans, similar to humans, show the protein prioritization pattern of macronutrient regulation. Through measuring physiological responses to fluctuations in macronutrient and energy intakes, our results have provided a model suggesting how this pattern of macronutrient regulation integrates with ecological variation on the one hand and flexible metabolic responses on the other (Fig. 5). A key point of this integration is that excess energy consumption is beneficial in the context of alternating periods of fruit scarcity and abundance. Because orangutans exceed energy expenditure by consuming energy-dense, protein-poor fruits during the high-fruit periods (*30*), this potentially provides a direct analog for the current global pandemic of obesity and metabolic disease in humans, which has arisen in parallel with transitions in food environments toward chronic exposure to low-cost, energy-dense, protein-dilute, ultra-processed foods (*99*). These products have been shown to interact with the protein prioritization pattern to drive energy overconsumption and have been widely associated with obesity and metabolic disease in humans in both nonlaboratory settings (*67*, *100*) and laboratory randomized control trials (*101*, *102*). Together, these associations might suggest that the current pandemic of metabolic disease has arisen not only because contemporary humans are chronically exposed to a high-energy environment but also because physiological systems evolved in a context of ecological variation and do not perform optimally when maintained in a chronic steady state (*2*). The same might explain the vulnerability of captive orangutans and other primate species to obesity and metabolic disease (*103*, *104*).

## MATERIALS AND METHODS

Behavioral observations (2003–2018) and urine sampling (2004–2017) of wild orangutans (*P. pygmaeus wurmbii*) were collected in the Tuanan Research Area located in the Mawas Conservation Area, Central Kalimantan, Indonesia [see (*30*, *33*) for a detailed site description]. Tuanan is a peat swamp forest with a peat depth of 1 to 3 m in most areas (*65*). Annual rainfall is between 1309.8 and 4176.0 mm with an average of 2602.4 mm, and minimum and maximum temperatures range between 20.5° and 32.0°C (average 23° to 28.5°C). For this study, we observed a total of 26 adult females, 48 adult flanged males, 25 adult unflanged males, 20 independent immatures, and 15 weaned immatures who still traveled with their mothers (table S8). All field research was approved by the Institutional Animal Care and Use Committee of Rutgers, the State University of New Jersey.

We collected behavioral data for the nutritional analyses using 2-min instantaneous scan samples following (*30*, *43*). Only data collected during full-day nest-to-nest focal follows were included in the analyses (*n* = 4873 follows and 52,289 hours; table S7). Each orangutan was followed by two observers for 2 to 10 consecutive days. Details of feeding rates, plant sample collection methods, laboratory nutritional analyses, and caloric and macronutrient intake data calculations are detailed in (*30*, *33*) and in the Supplementary Materials and Methods. We measured available protein (referred to as “protein”), total nonstructural carbohydrates (referred to as “carbohydrates”), lipids, and neutral detergent fiber (referred to as “fiber”). All data are reported in kcal from percent dry matter. To calculate the daily ratio of macronutrients, we took the sum of kcal consumed from one macronutrient and divided this sum by kcal consumed of the other macronutrients for a given day. For non-protein energy, we summed nonstructural carbohydrates, neutral detergent fiber, and lipids.

The FAI was determined from monthly monitored phenology plots that were spread across the study area, comprising between 1522 and 3103 tagged trees (see Supplementary Materials Methods). We calculated FAI as the percentage of trees in the plots with fruit each month (*30*, *33*). High- and low-fruit period categories were determined by calculating the overall median from 2003 to 2018, which was used as the cutoff point to assign low- and high-fruit periods to each month.

We collected urine samples in a plastic bag on a stick during the first-morning void, representing a 10- to 12-hour fast, typically when an orangutan emerged from its night sleep nest. For ketone bodies, urine was immediately pipetted from the plastic bag onto the ketone test pad until it was saturated, following the instructions of the Roche Diagnostics Chemstrip 10 UA, and the categorical level of ketone bodies was recorded after 60 s (*n* = 1115; table S9). The presence of ketone bodies followed the classification resulting from a validation on orangutans (*68*). The remaining urine sample was pipetted into a 15-ml collection vial and stored in an insulated thermos with ice until the end of each day, when samples were aliquoted into 1.8-ml microcentrifuge tubes and frozen at −18°C in a solar freezer in camp. Samples were transferred to −20°C in Jakarta until they were shipped on dry ice to Rutgers University, where they were stored at −80°C. In addition, two to five 200-μl aliquots of urine were pipetted onto Whatman 903 Proteinsaver filter cards, which were dried with silica gel and stored at room temperature on silica gel before shipping.

Following kit protocols, we analyzed urea concentration (*n* = 924) from frozen samples at Rutgers University using the QuantiChrom Urea Assay Kit (DIUR-500; linear detection range 0.08 to 100 mg/dl). All samples were run in duplicate and diluted at 1:10 or 1:20 with assay buffer. Intra- and interassay coefficient of variation were 2.68 and 11.62 (*n* = 30 plates; 929 samples; table S9), respectively. C-peptide of insulin was assayed from frozen samples with a commercially available competitive radioimmunoassay kit [Millipore Human C-peptide (HCP-20HK)], which has been previously validated for orangutans (*34*). All samples were run in duplicate and diluted at 1:2 or 1:4 with assay buffer. Intraassay coefficients of variations (CVs) were 5.35 and 4.78% for low and high controls, respectively, and interassay CVs were 7.88 and 6.41% for low and high controls (*n* = 10 kits; 580 samples; table S9). The sensitivity of the assay was 65 pg/ml. For both urea and C-peptide assays, samples with a CV > 15% were rerun or not included in the analyses. To control for the dilution of analytes by water, urea and C-peptide results were corrected with specific gravity (SG) following (*105*), where 1.023 is the mean SG value for the Tuanan population. SG was assessed in the field prior to freezing the sample using a hand-held refractometer that was calibrated daily (Atago PAL-10S). Because overly dilute samples yield exceedingly high adjusted results, we omitted samples with SG less than 1.002 (*n* = 12).

For isotopic analysis, urine was eluted from filter papers and prepared following (*n* = 195; table S9) (*55*, *106*). Nitrogen isotopes (δ^15^N) were analyzed in the Stable Isotope Biogeochemistry Facility at the University of Cincinnati by B.E.C. on a Costech Elemental Analyzer connected to a Thermo Scientific Delta V IRMS (Bremen, Germany) via a Costech Conflo IV interface (Valencia California, USA). We corrected data following (*107*). We normalized data for linearity and drift using powdered caffeine and corrected for scale using caffeine and USGS 41 glutamic acid. Accuracy (based on independent references powdered glycine and soy flour) was 0.08‰. Precision (based on all four reference materials) was 0.13‰.

### Statistical analyses

A breakdown of the distribution of sampling for all four urine analyses across age-sex class and across each sampling period is provided in fig. S8 and table S8. Full-day nest-to-nest daily nutritional intake data were matched with urine samples collected the following day (table S9). We excluded any urine samples that did not have a full day of nutritional intake from the prior day, which resulted in the following samples sizes: ketone presence, *n* = 782; C-peptide, *n* = 422; urea, *n* = 632; δ^15^N, *n* = 131. All statistical analyses were done in R (R Core Team 2023, version 4.2.2). Generalized additive mixed models (GAMMs) using Bayesian regression modeling with Stan were implemented using the “brms” package version 2.18.8 (*108*). We examined the correlation between macronutrient intakes with a hierarchical extension of Bayesian robust correlation as proposed by (*109*) and (*110*). The correlation between nutritional variables was modeled using an intercept-only random effects model with random intercepts α for each individual ID. Nutritional variables were modeled following a multivariate Student’s *t* distribution, with covariance matrix **S** and correlation matrix **R** (eqs. S1 and S2 and fig. S9A). We used the same method to examine correlations among biomarkers of energetic condition (fig. S9B).

To test for protein prioritization, we fit a hierarchical power function to model the relationship between total daily energy intake and percent energy from dietary protein (see eq. S3) (*60*, *61*). To understand orangutan physiological responses to variation in macronutrient intake and FAI, we fit multilevel distributional GAMMs (see the Supplementary Materials and tables S2 and S4). All nutritional variables were included in separate models to avoid concurvity as per the results of the Bayesian robust correlation analysis. To build intuitions about the distributions of the C-peptide, δ^15^N, and urea data and to select appropriate likelihood functions, the “fitdistrplus” package was used to estimate the best-fit distributions and their parameters.

Nitrogen isotope values followed a Gaussian distribution. Therefore, all models examining how nutritional intake predicts δ^15^N followed the form in eq. S4. The random effect structure was fully maximal (*111*) with random intercepts for each individual ID and random slopes on the nutritional variable, sex, and FAI. Priors were set to remain reasonably vague and to help with regularization. C-peptide and urea followed log-normal and gamma distributions, respectively (gamma distributions fit using method of moments using fitdistrplus). All models examining the effect of nutritional intake on C-peptide were log-normal models, and all models examining the effect of nutritional intake on urea were gamma models. All models were fit with vague priors. As with the δ^15^N models, the random effect structure was fully maximal, with penalized thin plate splines on macronutrient intake variables. All models followed the form in eqs. S5 and S6. As ketosis was measured with colorimetric Chemstrips, we modeled the effect of macronutrient intake on the presence/absence of ketone bodies, rather than modeling the probability of each colorimetric category. We modeled the effect of nutritional variables, sex, and FAI using a Bernoulli distribution. All ketone models followed the form in eq. S7.

Models were run for 7000 iterations over four Markov chain Monte Carlo (MCMC) chains, with a warm-up period of 3500 iterations per chain. Initial values for all gamma models were set to 0, with all other models allowing for random initial values between −2 and 2. Visual inspection of diagnostic plots showed stationarity and convergence, all Rhat values were below 1.01, and there were no divergent transitions after warm-up. Graphical posterior predictive checks indicated that model structures recovered the behavior of our data well. For all models reported, loo, Pareto *k*, and Pareto smoothed importance sampling (PSIS) values showed no evidence of overfitting.

To facilitate the interpretation of the fitted smooth functions, we identified the portions along the fitted splines where the rate of change in the response (slope), was different from 0 (not flat) with 95% credibility. We identified sections along each spline where nutritional intakes and urinary biomarkers were increasing or decreasing with very little posterior uncertainty (i.e., at 95% credibility). To do this, we estimated the first derivatives of the fitted splines using the method of finite differences (*112*, *113*) but used a fully Bayesian implementation of this approach (*114*, *115*). Posterior predictions of smooth terms were computed using the “posteriorsmooths” function in brms. The data were then shifted in by a very small amount, and poster predictions were computed again from the shifted data. We estimated the first derivative by computing the differences between the two sets of posterior predictions, divided by the magnitude of the shift. We considered areas where the posterior uncertainty interval on first derivatives excludes zero to indicate statistically nonzero rates of change. All data needed to evaluate the conclusions in the paper are present in the paper and/or the Supplementary Materials. All code for analyses is hosted on Zenodo (https://zenodo.org/records/10451962) (*116*), and all code for estimating the first derivative of splines is hosted on Zenodo (https://doi.org/10.5281/zenodo.7457199) (*115*). The data file is available online (https://doi.org/10.5061/dryad.c59zw3rjx).

## Supplementary Materials

### This PDF file includes:

Supplementary Materials and Methods Figs. S1 to S9 Tables S1 to S9 Data S1 References
