# Supplementary material for: Integrated behavioral and metabolically flexible responses of wild orangutans to ecologically driven dietary variation
Source: Sci Adv. 2025 Aug 27;11(35):eadv7613. doi: 10.1126/sciadv.adv7613 (PMC12383276; doi:10.1126/sciadv.adv7613)
Supplement: Supplementary file 1 — Supplementary Materials and Methods Figs. S1 to S9 Tables S1 to S9 Data S1 References [file sciadv.adv7613_sm.pdf]

Science Advances
AAAS

Supplementary Materials for

Integrated behavioral and metabolically flexible responses of wild orangutans to ecologically driven dietary variation

Erin R. Vogel *et al.*

Corresponding author: Erin R. Vogel, erin.vogel@rutgers.edu

Sci. Adv. **11**, eadv7613 (2025)
DOI: 10.1126/sciadv.adv7613

This PDF file includes:

Supplementary Materials and Methods
Figs. S1 to S9
Tables S1 to S9
Data S1
References

Supplementary Text

Methods

Plant sampling for nutritional analyses:

Food samples for this study were collected between June 2004 and September 2016 by selecting food items from trees where the orangutans had been observed to feed (see *1 for a description of sample collection methods*). Following standard techniques, all nutritional analyses were conducted in the Laboratory of Nutrition Testing in the Research Center for Biology at the Indonesian Institute of Sciences (LIPI) (*33, 31*). We followed the nutritional methods and energetic calculations reported by Vogel and colleagues *(30, 33*), except for estimating available protein. We could not conduct protein analyses for this study because this analysis was not available in Indonesia, and export permits could not be obtained. Thus, we estimated available protein from crude protein values by using conversion coefficients for similar food items from Indonesia (*118*). We realize this is not ideal, but since similar foods are sampled, our available protein estimates may only slightly over- or underestimate actual available protein. All nutritional results are reported as 100% dry matter (DM) (*119*).

Fruit Availability Index (FAI)

Surveys for estimating the number of fruiting trees in the phenology plots started on the 10th of each month and typically took between 2-6 days to complete. Data were collected by the same field staff for each month. From July 2003 through October 2015, phenology data were collected from a 2.3-ha strip plot along three different transects across the study area. In November 2015, an additional three transects were added to the phenology plots and monitored monthly, resulting in a total of 3.2-ha strip plots from November 2015 through December 2018. The minimum stem size criteria to include trees in the phenology plots was 10 cm diameter at breast height and new trees were evaluated for addition to the plots approximately each year. The total number of trees in the phenology plots was on average 2124 trees (standard deviation = 724) and ranged from 1,522 to 3,103 trees across the study period.

Energy intake estimates: limitations

Energy intake per feeding bout was calculated following (*30*, *31*). Feeding bouts were summed across each follow day to obtain total daily caloric intake (Kcal). If nutritional data and/or feeding rates were not available for a given plant species, we used 1) values from a different species within the same Genus with similar fruit size/character or, if this was not available, 2) the average value for that item (fruit, leaves, inner-bark, flowers, vegetation) and stage of ripeness across all food species When available, we used sex-specific feeding rates for each species. However, when these data were unavailable for a given sex, average feeding rates obtained for the other sex were used, as Vogel and colleagues (*33*) found that feeding rates do not differ between sexes.

One limitation of our study that could affect our estimates of energy assimilated from fiber (neutral detergent fiber) and protein is the digestibility and availability estimates that we used.

We assumed a low energy fiber digestibility coefficient for orangutans (*30, 120*), and thus energy from fiber may be underestimated in our study. While much is known about human digestibility of fiber, including the role the microbiome plays (*121*), little is known about how much energy wild orangutans acquire from both the gut microbes and fiber and we are reliant on estimates of fiber derived energy from captive experiments (*120*). There is a possibility of an increase in propionate production through the fermentation of soluble fiber, since propionate is a gluconeogenic precursor (*121, 122*). Future research on the gut microbiome and the role of propionate producing microbes in maintaining energy balance in orangutans will provide valuable information on the link between dietary fiber and energetics. Likewise, our estimate of available protein does not consider the protein-binding effects of tannins and other plant secondary metabolites (PSM) and may lead to overestimations of energy from protein (*123, 124*). To gain a more accurate picture of orangutan nutritional ecology and physiology, future research should focus on how ecological variation interacts with 1) the gut microbiome and the role of propionate producing microbes in maintaining energy balance in orangutans and 2) PSM and their protein-binding effects in orangutan foods.

Statistical Analyses

Protein Prioritization

To test for protein prioritization, we fit a hierarchical power function to model the relationship between total daily energy intake and percent energy from dietary protein (*60, 125*). This test follows from the fact that to maintain protein energy, P, more constant than non-protein energy on diets varying in the proportion of energy from protein, p, Pp^L calories must be eaten, where $-1 < L < 0$. The case where $L = -1$ indicates complete protein prioritization (i.e., protein intake constant). L increases as protein regulation becomes weaker, with values greater than 0 indicating NPE prioritization (i.e., NPE intake is maintained more constant than protein).

Physiological Responses to Variation in Macronutrient Intake and Fruit Availability Index (FAI)

For all models, we fit multilevel distributional generalized additive models (GAM) (see Supplementary Materials, Statistical Analyses, and table S2, S4). Generalized additive models (GAM) are semi-parametric extensions of generalized linear models that allow the estimation of non-linear patterns in data without any prior knowledge of the shape of the expected relationship. The linear predictor is a sum of smooth functions of the predictor variables, smooth terms are represented using penalized regression splines, and inference is based on these smooth functions (*125, 126*).

The equations used in the models described under Statistical Analyses are outlined below.

SI Methods Statistical Analyses Equation 1.

$$\mathbf{Y}_{ijkl} = \begin{bmatrix} Y_{ijkl}^{\text{Protein}} \\ Y_{ijkl}^{\text{TNC}} \\ Y_{ijkl}^{\text{Lipid}} \\ Y_{ijkl}^{\text{NDF}} \end{bmatrix} = \begin{bmatrix} \alpha_1 + u_{1j[ik]} \\ \alpha_2 + u_{2j[ik]} \\ \alpha_3 + u_{3j[ik]} \\ \alpha_4 + u_{4j[ik]} \end{bmatrix} + \begin{bmatrix} \epsilon_{ijkl}^{\text{Protein}} \\ \epsilon_{ijkl}^{\text{TNC}} \\ \epsilon_{ijkl}^{\text{Lipid}} \\ \epsilon_{ijkl}^{\text{NDF}} \end{bmatrix}$$

The random effects structure is:

$$\mathbf{u}_j = \begin{bmatrix} u_{1j} \\ u_{2j} \\ u_{3j} \\ u_{4j} \end{bmatrix} \sim \text{Normal}\left(\mathbf{0}, \mathbf{L}_{\text{RE}}\mathbf{D}_{\text{RE}}\mathbf{L}_{\text{RE}}^{\top}\right)$$

Priors for the fixed effects and random effects parameters are:

$$\begin{aligned} \alpha_k &\sim \text{Student-t}\,(3, 0, 100) \\ \mathbf{L}_{\text{RE}} &\sim \text{LKJCorr}\,(1) \\ \text{diag}(\mathbf{D}_{\text{RE}}) &\sim \text{Student-t}\,(3, 0, 100) \end{aligned}$$

The residual covariance matrix is modeled as:

$$\mathbf{R} = \mathbf{L}_{\text{Res}}\mathbf{D}_{\text{Res}}\mathbf{L}_{\text{Res}}^{\top}$$

All variables share the same ν parameter with ν restricted to small values to better handle multivariate outliers (*110*). The residual correlation ρ is the parameter of interest, indicating the correlation among variables after accounting for unobserved heterogeneity due to individual identity.

SI Methods Statistical Analyses Equation 2.

Response Variables:

$Y_{\log(\text{Urea})} = (y_{1,\log(\text{Urea})}, \ldots, y_{N_{\log(\text{Urea})},\log(\text{Urea})})'$

$Y_{\log(\text{UCP})} = (y_{1,\log(\text{UCP})}, \ldots, y_{N_{\log(\text{UCP})},\log(\text{UCP})})'$

$Y_{\delta^{15}N} = (y_{1,\delta^{15}N}, \ldots, y_{N_{\delta^{15}N},\delta^{15}N})'$

$Y = \left[Y_{\log(\text{Urea})}\ Y_{\text{logucpsgcor}}\ Y_{\delta^{15}N}\right]$

Fixed Effects:

$\mu_{\log(\text{Urea})} = \alpha_{\log(\text{Urea})} + \mathbf{Z}1, \log(\text{Urea})\mathbf{r}1, \log(\text{Urea})$

$\mu_{\log(\text{UCP})} = \alpha_{\log(\text{UCP})} + \mathbf{Z}2, \log(\text{UCP})\mathbf{r}2, \log(\text{UCP})$

$\mu_{\delta^{15}N} = \alpha_{\delta^{15}N} + \mathbf{Z}3, \delta^{15}N\mathbf{r}3, \delta^{15}N$

Random Effects:

$\mathbf{r}1, \log(\text{Urea}) \sim \mathcal{N}(0, \mathbf{D}1)$

$\mathbf{r}2, \log(\text{UCP}) \sim \mathcal{N}(0, \mathbf{D}2)$

$\mathbf{r}3, \delta^{15}N \sim \mathcal{N}(0, \mathbf{D}3)$

Residual Correlation:

$\Sigma = \mathbf{L}\text{rescor}\mathbf{D}\text{diag}\mathbf{L}\text{rescor}'$

Multivariate Model:

$Y \sim \text{Multi-Student-t}(\nu, \boldsymbol{\mu}, \Sigma)$

Prior Distributions:

$\alpha\log(\text{Urea}) \sim \text{Student-t}(3, 0, 10000)$

$\sigma_{\log(\text{Urea})} \sim \text{Half-Student-t}(3, 0, 2.5)$

$\alpha_{\log(\text{UCP})} \sim \text{Student-t}(3, 0, 10000)$

$\sigma_{\log(\text{UCP})} \sim \text{Half-Student-t}(3, 0, 2.5)$

$\alpha_{\delta^{15}N} \sim \text{Student-t}(3, 0, 10000)$

$\sigma_{\delta^{15}N} \sim \text{Half-Student-t}(3, 0, 2.5)$

Random Effect Priors:

$\mathbf{D}1 \sim \text{Wishart}(\mathbf{S}1, \nu_1)$

$\mathbf{D}2 \sim \text{Wishart}(\mathbf{S}2, \nu_2)$

$\mathbf{D}3 \sim \text{Wishart}(\mathbf{S}3, \nu_3)$

Residual Correlation Prior:

$\mathbf{L}\text{rescor} \sim \text{LKJ}(\eta)$

Degrees of Freedom Prior:

$\nu \sim \text{Gamma}(a\nu, b_\nu)$

SI Methods Statistical Analyses Equation 3.

Let Y_i represent total caloric intake

$$
\begin{aligned}
y_i &\sim \text{Gamma}(\text{shape}, \mu_i) \\
\mu_i &= \frac{\text{shape}}{b_1^{(i)}(\text{Percent Protein}_i)^{a^{(i)}}} \\
\\
b_1^{(i)} &= \beta_{b_1} + u_{b_1}^{(\text{Orangutan}_i)} \\
a^{(i)} &= \beta_a + u_a^{(\text{Orangutan}_i)} \\
\\
u_{b_1}^{(k)} &\sim \mathcal{N}(0, \sigma_{b_1}^2) \\
u_a^{(k)} &\sim \mathcal{N}(0, \sigma_a^2) \\
\\
\beta_{b_1} &\sim \mathcal{N}(0, 99999^2) \\
\beta_a &\sim \mathcal{N}(0, 99999^2) \\
\sigma_{b_1} &\sim \text{HalfStudentT}(3, 0, 1449.1) \\
\sigma_a &\sim \text{HalfStudentT}(3, 0, 1449.1) \\
\text{shape} &\sim \text{Gamma}(0.01, 0.01)
\end{aligned}
$$

SI Methods Statistical Analyses Equation 4.

Let Y_i represent $\delta^{15}N$

$$\mathrm{Y}_i \sim \mathcal{N}(\mu_i, \sigma^2),\ i = 1, \ldots, N$$

$$\mu_i = \beta_0 + X_i\beta + f_1(\mathrm{FAI}_i) + f_2(\mathrm{Nutrient}_i) + f_3(\text{time-collected}_i) + u_{0,\mathrm{Orangutan}_i} + u_{1,\mathrm{Orangutan}_i} \times \mathrm{FAI}_i + u_{2,\mathrm{Orangutan}_i} \times \mathrm{Nutrient}_i + u_{3,\mathrm{Orangutan}_i} \times \text{time-collected}_i$$

Fixed Effects:

$\beta = (\beta_{\text{Age-class}}, \beta_{\text{Fai}}, \beta_{\text{Nutrient}}, \beta_{\text{Collection time}})$

$\beta_0, \beta \sim \mathcal{N}(0, 10^2)$

$\mathrm{bs} \sim \mathcal{N}(0, 10^2)$

$\mathrm{sds}_1, \mathrm{sds}_2, \mathrm{sds}_3 \sim \text{Student-t}(3, 0, 2.5)$

$\sigma \sim \mathcal{N}(0, 10^2)$

$\mathrm{sd}_1 \sim \mathcal{N}(0, 10^2)$

$L_1 \sim \mathrm{LKJ}(1)$

$f_1(\mathrm{FAI}_i) = Z_{\mathrm{s}_1,i} s_1$

$f_2(\mathrm{Nutrient}_i) = Z_{\mathrm{s}_2,i} s_2$

$f_3(\text{time-collected}_i) = Z_{\mathrm{s}_3,i} s_3 u_{j,\mathrm{Orangutan}_i} \sim \mathcal{N}(0, (\mathrm{sd}_1)_j^2),\ j = 0, 1, 2, 3$

$\mathrm{Cor}_1 = L_1 L_1$

SI Methods Statistical Analyses Equation 5.

Let Y_i represent Urinary C-Peptide

$$Y_i \sim \text{LogNormal}(\mu_i, \sigma)$$
$$\mu_i = \alpha + X_{c_i}\beta + f_1(FAI_i) + f_2(Nutrient_i) + f_3(Collection\ time_i) + \boldsymbol{u}(Orang_i)$$
$$\alpha = \alpha_0 - \bar{\boldsymbol{X}} \cdot \beta$$
$$\boldsymbol{u}(Orang_i) = r_1(Orang_i) \cdot Z_{1_{1_i}} + r_2(Orang_i) \cdot Z_{1_{2_i}} + r_3(Orang_i) \cdot Z_{1_{3_i}} + r_4(Orang_i) \cdot Z_{1_{4_i}}$$
$$\boldsymbol{r}(Orang_i) \sim \text{MultivariateNormal}(\boldsymbol{0}, \boldsymbol{\Sigma})$$
$$\boldsymbol{\Sigma} = \text{diag}(\boldsymbol{\sigma}_u) \cdot \boldsymbol{R} \cdot \text{diag}(\boldsymbol{\sigma}_u)$$
$$\boldsymbol{R} = \boldsymbol{L} \cdot \boldsymbol{L}^T$$

Fixed Effects:

$$\beta = (\beta_{\text{Age-class}}, \beta_{\text{Fai}}, \beta_{\text{Nutrient}}, \beta_{\text{Collection time}})$$

Splines:
$$f_1(FAI_i) = Z_{s_{1i}} \boldsymbol{s}_1$$
$$f_2(Nutrient_i) = Z_{s_{2i}} \boldsymbol{s}_2$$
$$f_3(Collection\ time_i) = Z_{s_{3i}} \boldsymbol{s}_3$$

Priors:
$$\alpha_0 \sim \text{Normal}(0, 10)$$
$$\beta \sim \text{Normal}(0, 10)$$
$$\boldsymbol{s}_1 \sim \text{Normal}(0, \sigma_{s_1})$$
$$\boldsymbol{s}_2 \sim \text{Normal}(0, \sigma_{s_2})$$
$$\boldsymbol{s}_3 \sim \text{Normal}(0, \sigma_{s_3})$$
$$\sigma \sim \text{Normal}^+(0, 10)$$
$$\boldsymbol{\sigma}_u \sim \text{Normal}^+(0, 10)$$
$$\boldsymbol{L} \sim \text{LKJCorr}(1)$$
$$\sigma_{s_1} \sim \text{Student-t}^+(3, 0, 2.5)$$
$$\sigma_{s_2} \sim \text{Student-t}^+(3, 0, 2.5)$$
$$\sigma_{s_3} \sim \text{Student-t}^+(3, 0, 2.5)$$

Random Effects:
$$\boldsymbol{u}(\text{Orang}_i) = \boldsymbol{Z}_{u_i} \boldsymbol{r}(Orang_i)$$

Design Matrices for the Random Effects:
$$\boldsymbol{Z}_{u_i} = (Z_{1_{1_i}}, Z_{1_{2_i}}, Z_{1_{3_i}}, Z_{1_{4_i}})$$

Priors for Random Effects:
$$\boldsymbol{r}(Orang_i) \sim \text{MultivariateNormal}(\boldsymbol{0}, \boldsymbol{\Sigma})$$
$$\boldsymbol{\Sigma} = \text{diag}(\boldsymbol{\sigma}_u) \cdot \boldsymbol{R} \cdot \text{diag}(\boldsymbol{\sigma}_u)$$
$$\boldsymbol{R} = \boldsymbol{L} \cdot \boldsymbol{L}^T$$
$$\boldsymbol{\sigma}_u \sim \text{Normal}^+(0, 10)$$
$$\boldsymbol{L} \sim \text{LKJCorr}(1)$$

SI Methods Statistical Analyses Equation 6.

Let y_i represent Urea

$$
\begin{aligned}
y_i &\sim \text{Gamma}(\alpha, \mu_i)\\
\mu_i &= \alpha \exp(-\eta_i)\\
\eta_i &= \beta_0 + \mathbf{X}_{c_i}\boldsymbol{\beta} + \mathbf{X}_{s_i}\boldsymbol{\beta}_s + f_1(\text{FAI}_i) + f_2(\text{Nutrient}_i)\\
&\quad + f_3(\text{Collection time}_i) + (\mathbf{Z}_{1_i}\boldsymbol{u}_{1_{Orangutan_i}}) + (\mathbf{Z}_{2_i}\boldsymbol{u}_{2_{Orangutan_i}}) + (\mathbf{Z}_{3_i}\boldsymbol{u}_{3_{Orangutan_i}}) + (\mathbf{Z}_{4_i}\boldsymbol{u}_{4_{Orangutan_i}})\\
\boldsymbol{\beta} &= (\beta_{\text{Age-class}}, \beta_{\text{Fai}}, \beta_{\text{Nutrient}}, \beta_{\text{Collection time}})\\
\boldsymbol{\beta} &\sim \mathcal{N}(0, 10^2\mathbf{I})\\
\boldsymbol{\beta}_s &\sim \mathcal{N}(0, 10^2\mathbf{I})\\
f_j(x) &= \mathbf{Z}_{s_j}(x)\mathbf{s}_j, \quad j = 1, 2, 3\\
\mathbf{s}_j &\sim \mathcal{N}(0, (\text{sd}_j)^2\mathbf{I})\\
\text{sd}_j &\sim \text{Student-t}(3, 0, 2.5)\\
\alpha &\sim \text{Gamma}(0.01, 0.01)\\
\boldsymbol{u}_k &\sim \mathcal{N}(0, \mathbf{L}_k\text{diag}(\text{sd}_k)^2\mathbf{L}_k^T), \quad k = 1, 2, 3, 4\\
\text{sd}_k &\sim \mathcal{N}(0, 10^2), \quad k = 1, 2, 3, 4\\
\mathbf{L}_k &\sim \text{LKJ}(1)
\end{aligned}
$$

SI Methods Statistical Analyses Equation 7.

Let y_i represent Ketone presence/absence:

$$
\begin{aligned}
y_i &\sim \text{Bernoulli}(p_i) \\
\text{logit}(p_i) &= \beta_0 + f_1(\text{FAI}_i) + \beta_{\text{class}}[\text{Age-Class}_i] + f_2(\text{Nutrient}_i) \\
&\quad + \gamma_{0j[i]} + \gamma_{1j[i]} \cdot \text{FAI}_i + \gamma_{2j[i]} \cdot \text{Nutrient}_i \\
f_k(x) &= \sum_{m=1}^{M_k} \theta_{km} B_{km}(x) \quad k = 1, 2 \\
\beta_0 &\sim \text{Student-t}(4, 0, 1.5) \\
\beta_{\text{class}} &\sim \text{Student-t}(4, 0, 1.5) \\
\theta &\sim \text{Student-t}(4, 0, 1.5) \\
\gamma_{0j}, \gamma_{1j}, \gamma_{2j} &\sim \text{MVN}(0, \Sigma) \\
\Sigma &= \text{diag}(\sigma_1^2, \sigma_2^2, \sigma_3^2) \cdot \text{Corr} \cdot \text{diag}(\sigma_1^2, \sigma_2^2, \sigma_3^2) \\
\sigma_k &\sim \text{Student-t}(4, 0, 1.5) \quad k = 1, 2, 3 \\
\text{Corr} &\sim \text{LKJ}(1)
\end{aligned}
$$

where γ_{0j}, γ_{1j}, and γ_{2j} are the random effects for group j

(corresponding to the individual orangutans)

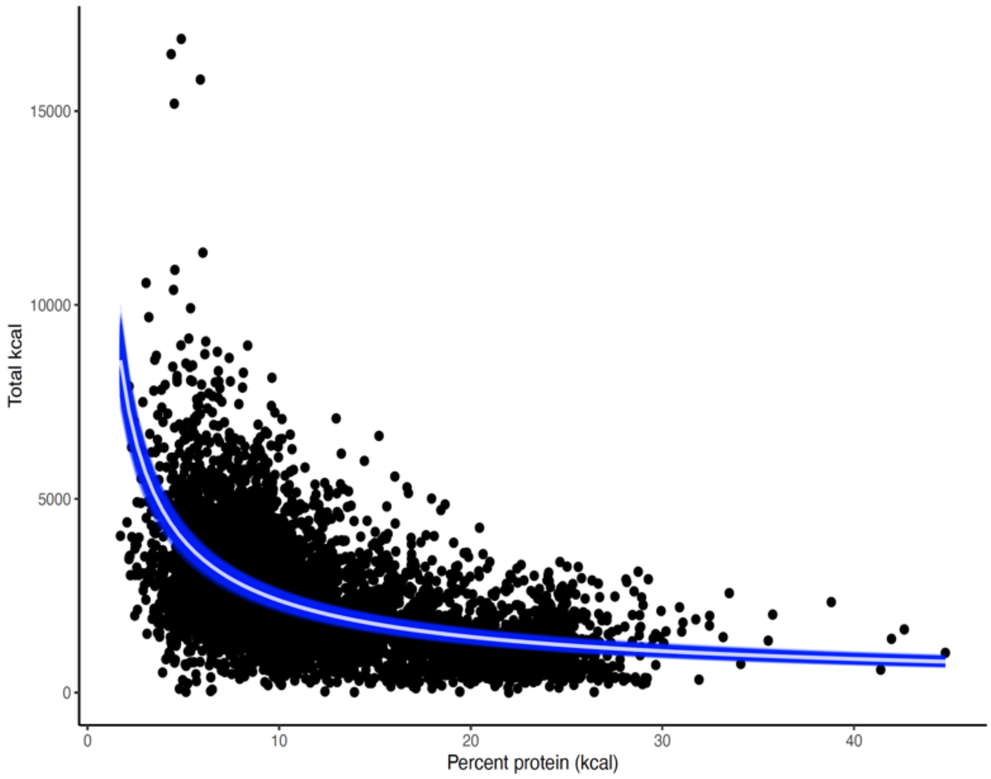

fig. S1. Posterior predictive plot of the power model predicting the relationship between total daily caloric intake and percent of protein intake.

$$Total\ caloric\ intake = \boldsymbol{\beta_1} * Percent\ Protein^{\alpha}$$

Data represent 4873 full-day focal animal follows and both $\boldsymbol{\beta_1}$ and $\boldsymbol{\alpha}$ were modeled with Orangutan ID as a random effect. $\boldsymbol{\beta_1}$ is the slope and $\boldsymbol{\alpha}$ = intercept. $r^2 = 0.39$. Results of the analysis can be found in table S1. The model details are found in the Supplementary Materials, Statistical Analysis Equation 3.

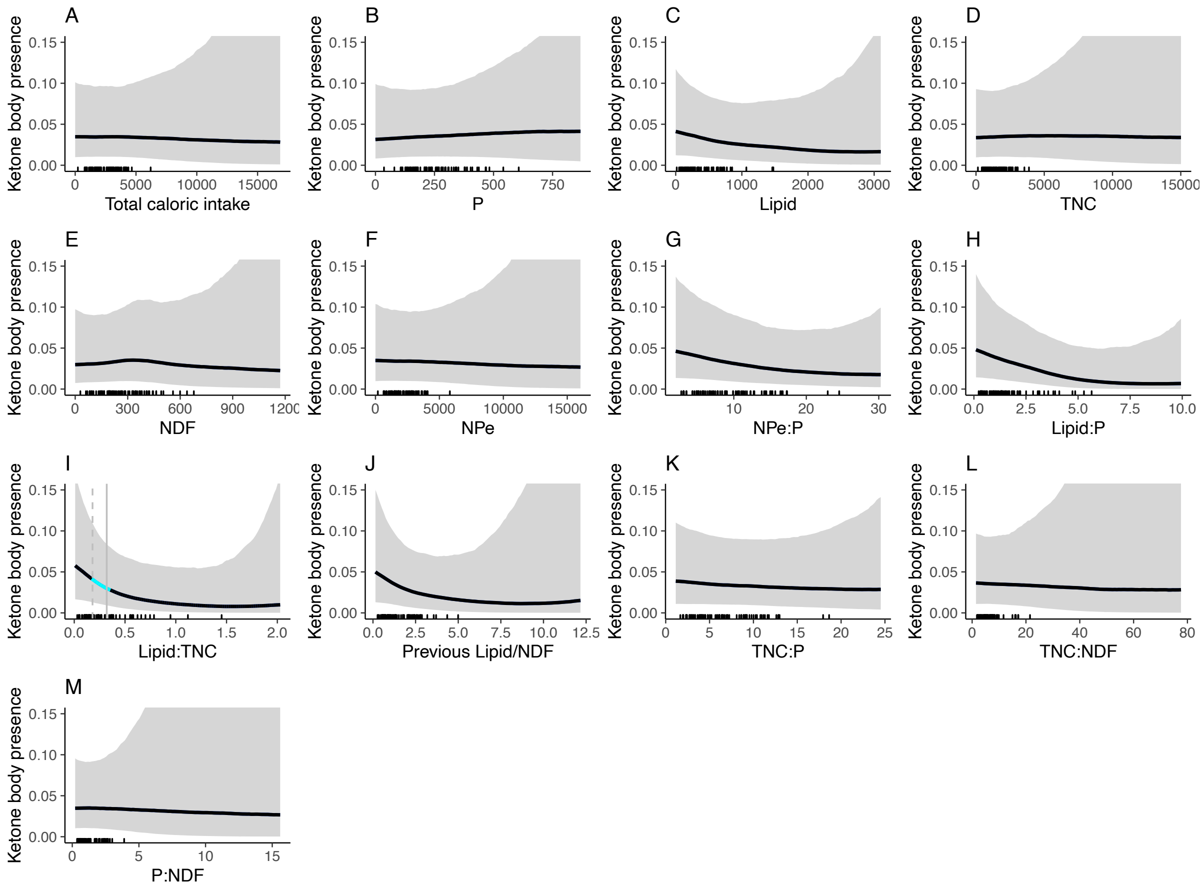

fig. S2. The presence/absence of urinary ketone bodies in relation to macronutrient intake during the study period. For ketone-body presence the scale is from 0 to 1, however the y-axis is zoomed in on to better illustrate any relationships. The dashed gray line represents the mode and the solid gray line represents the mean for the intake. Mean and mode are only indicated for panels with a real response. The light blue sections of the curve represent the periods along the fitted splines where the rate of change in the response (slope), was different from 0 (not flat) with 95% credibility. The rug plot represents the actual data distribution. The model details are found in SI Statistical Analysis Equation 7. Total kcal = total daily caloric intake; NPe = non-protein energy; TNC = total non-structural carbohydrates; NDF = neutral detergent fiber; P = available protein**.**

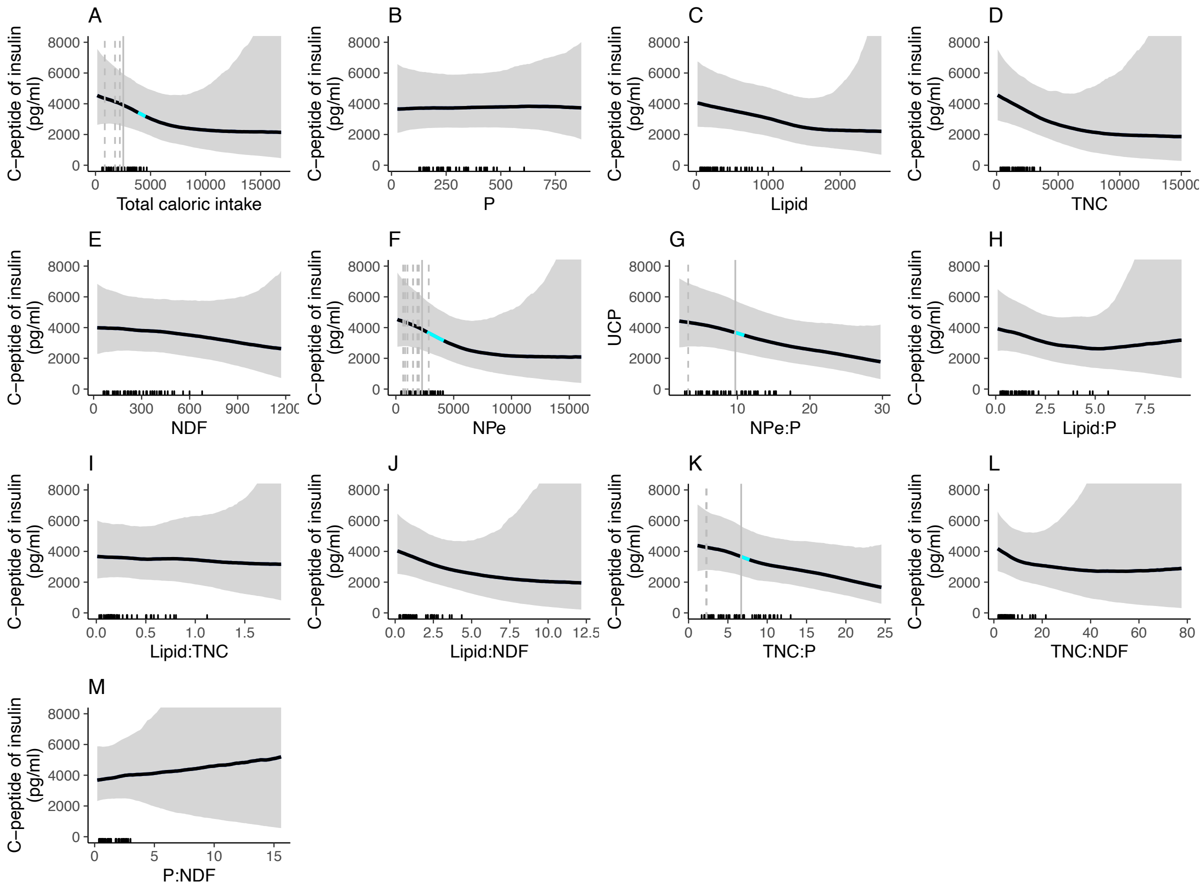

fig. S3. Urinary C-peptide of insulin (pg/ml) in relationship to macronutrient intake during the study period. The dashed gray line represents the mode and the solid gray line represents the mean for the intake. Mean and mode are only indicated for panels with a real response. The light blue sections of the curve represent the periods along the fitted splines where the rate of change in the response (slope), was different from 0 (not flat) with 95% credibility. The rug plot represents the actual data distribution. The model details are found in SI Statistical Analysis Equation 5. Total kcal = total daily caloric intake; NPe = non-protein energy; TNC = total non-structural carbohydrates; NDF = neutral detergent fiber; P = available protein.

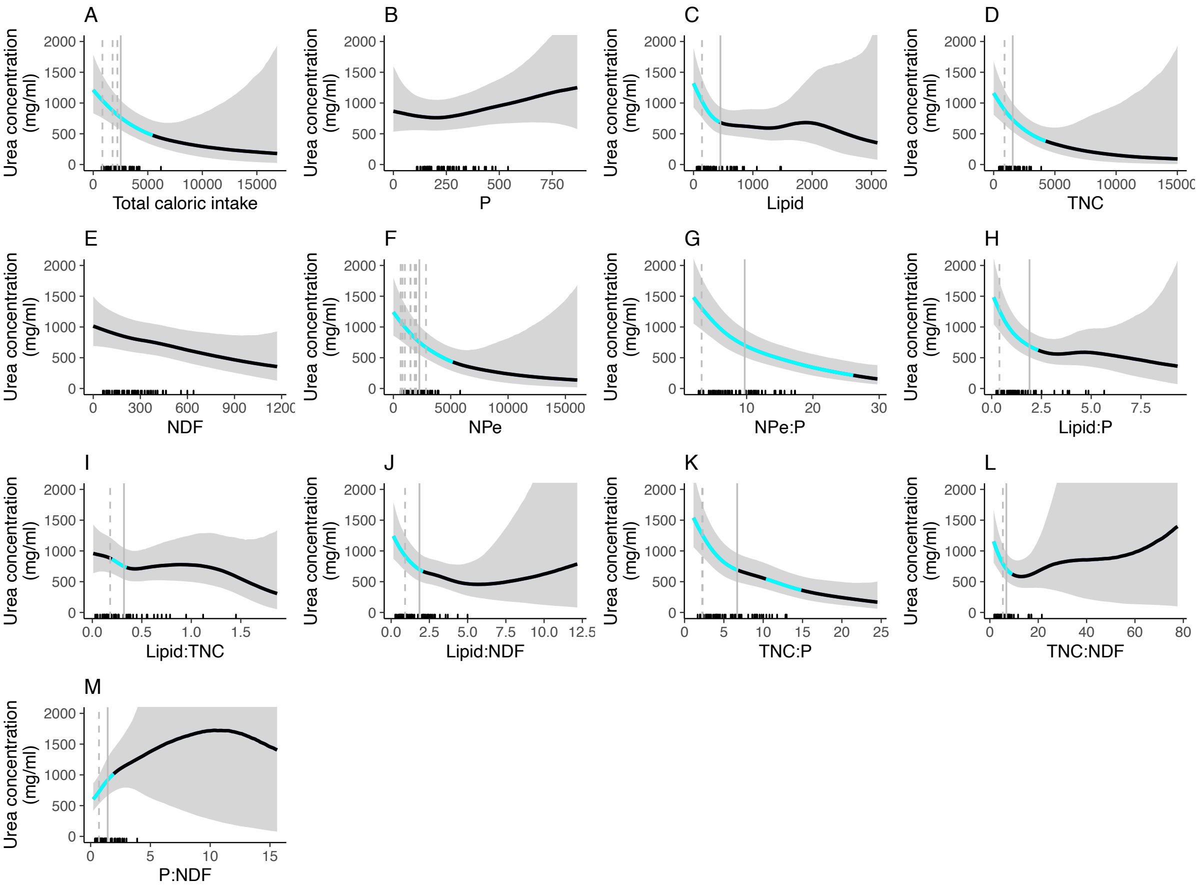

fig. S4. Urinary urea concentration (mg/ml) in relationship to macronutrient intake during the study period. The dashed gray line represents the mode and the solid gray line represents the mean for the intake. Mean and mode are only indicated for panels with a real response. The light blue sections of the curve represent the periods along the fitted splines where the rate of change in the response (slope), was different from 0 (not flat) with 95% credibility. The rug plot represents the actual data distribution. The model details are found in SI Statistical Analysis Equation 6. Total kcal = total daily caloric intake; NPe = non-protein energy; TNC = total non-structural carbohydrates; NDF = neutral detergent fiber; P = available protein.

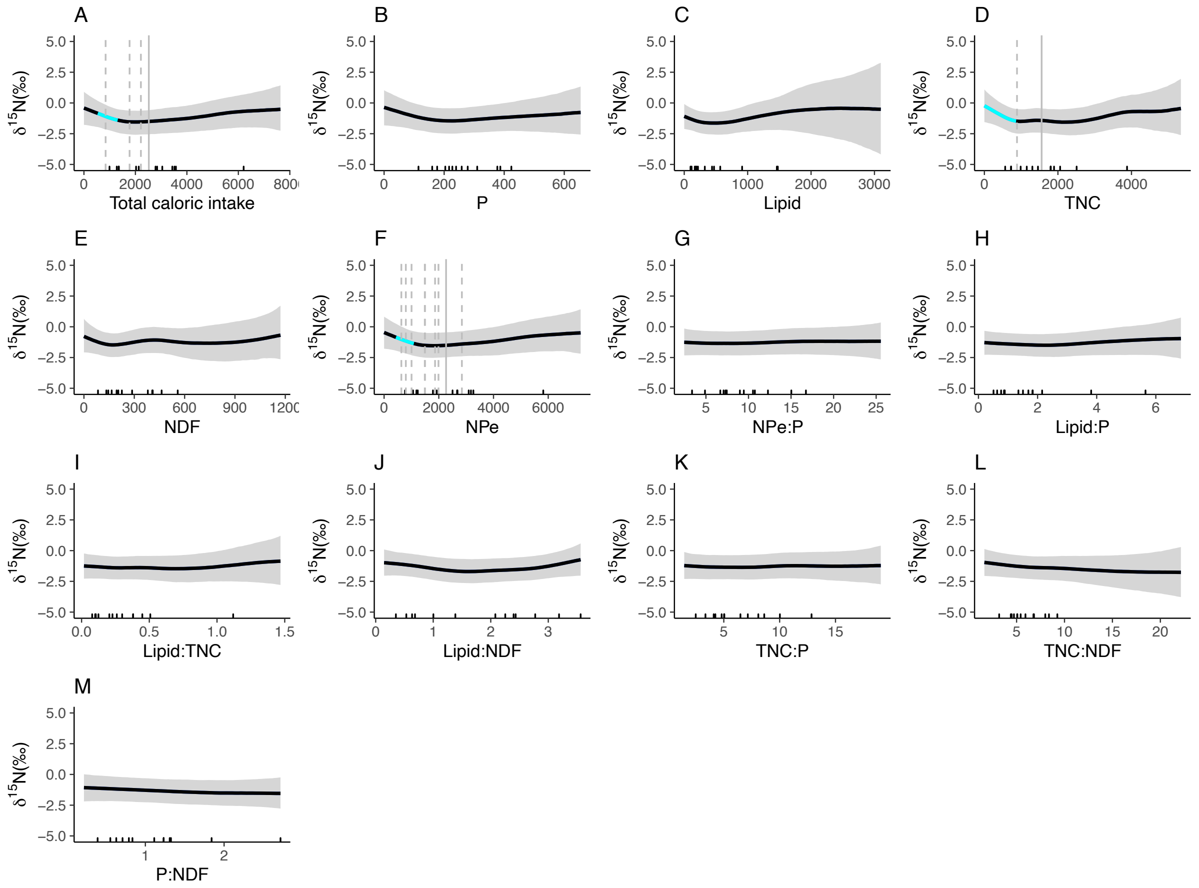

fig. S5. Urinary $\delta^{15}N$ in relationship to macronutrient intake during the study period. The dashed gray line represents the mode and the solid gray line represents the mean for the intake. Mean and mode are only indicated for panels with a real response. The light blue sections of the curve represent the periods along the fitted splines where the rate of change in the response (slope), was different from 0 (not flat) with 95% credibility. The rug plot represents the actual data distribution. The model details are found in SI Statistical Analysis Equation 4. Total kcal = total daily caloric intake; NPe = non-protein energy; TNC = total non-structural carbohydrates; NDF = neutral detergent fiber; P = available protein.

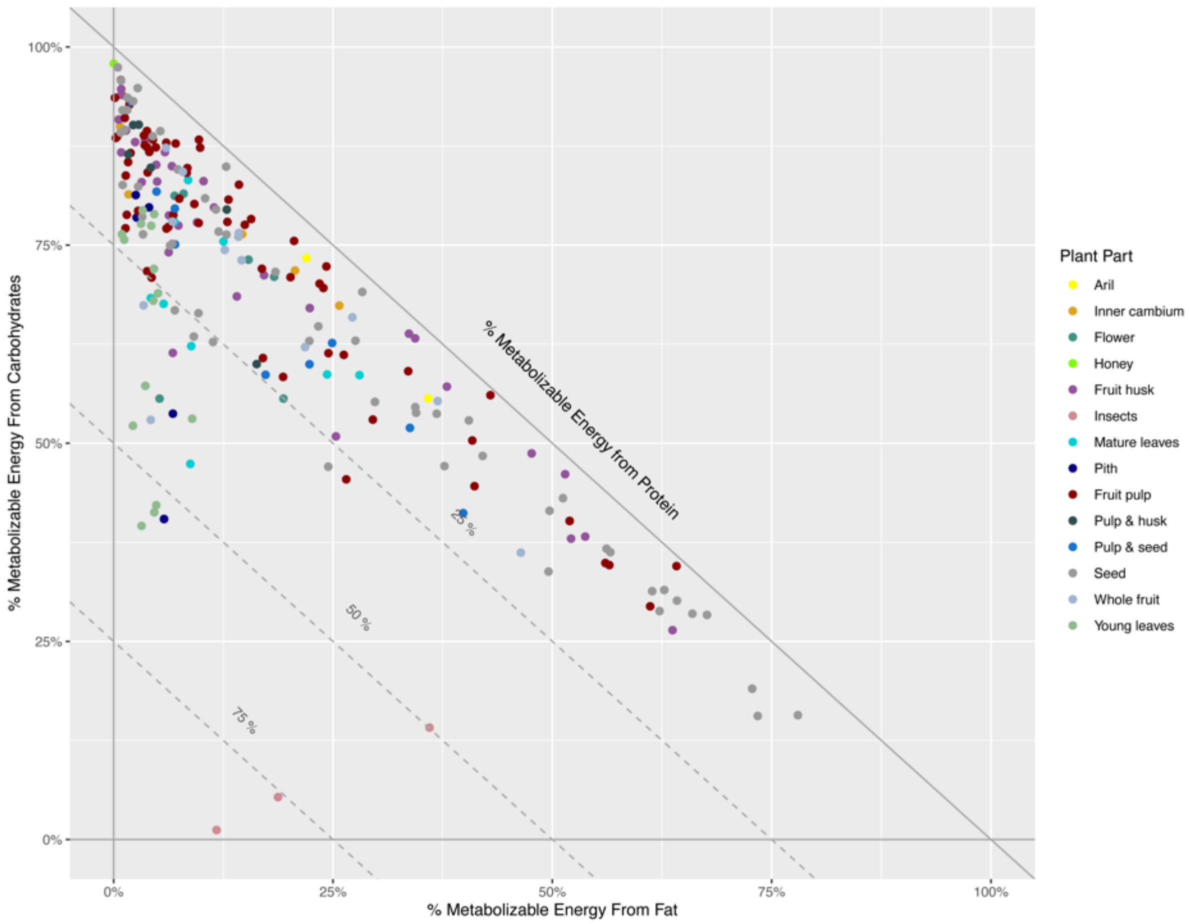

fig. S6. Right-angle mixture model of food items with macronutrients in the diet of Tuanan orangutans. Each circle represents a different food species (n=225 species item combinations consumed by orangutans).

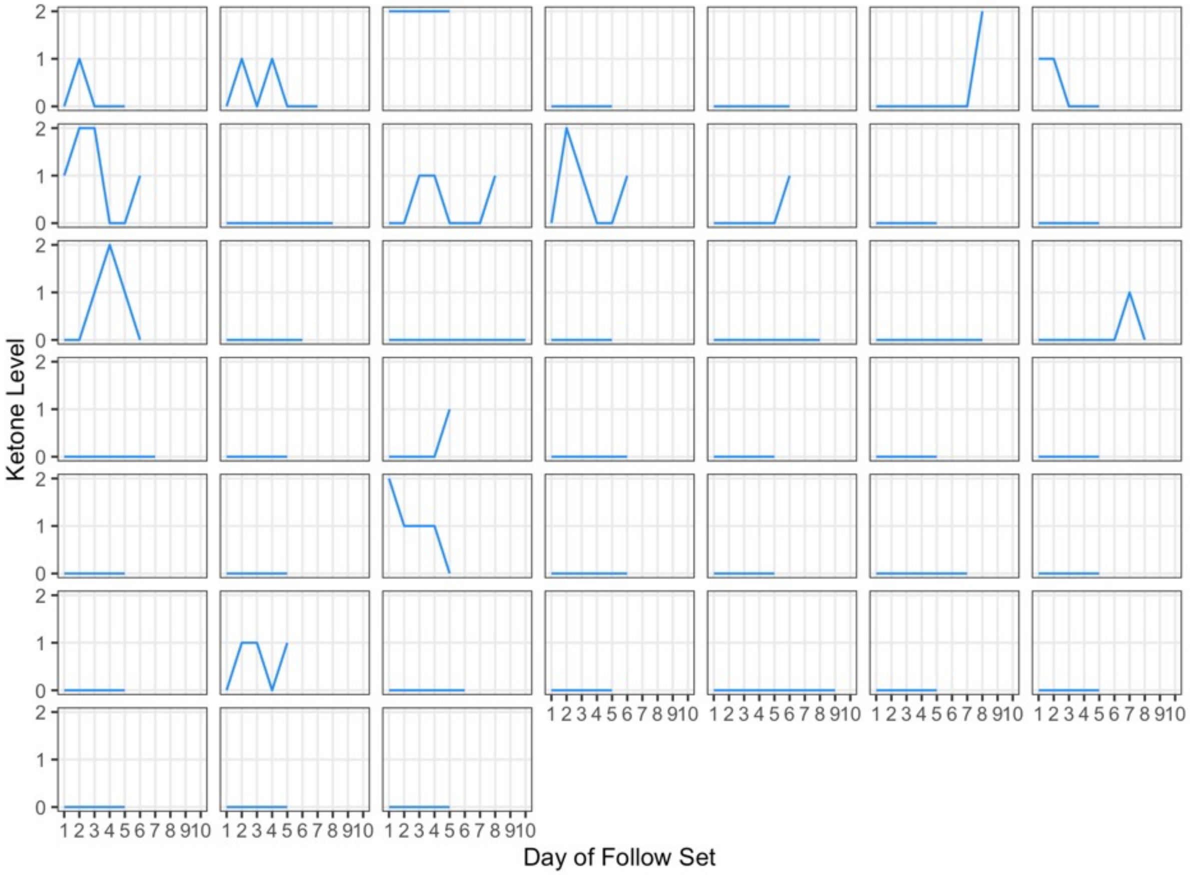

fig. S7. Graphical representation of the variation in ketone body presence measured with qualitative Chemstrips over different follow periods across different individuals. Each follow set is for a different individual and ketone body levels are defined as in (*68*).

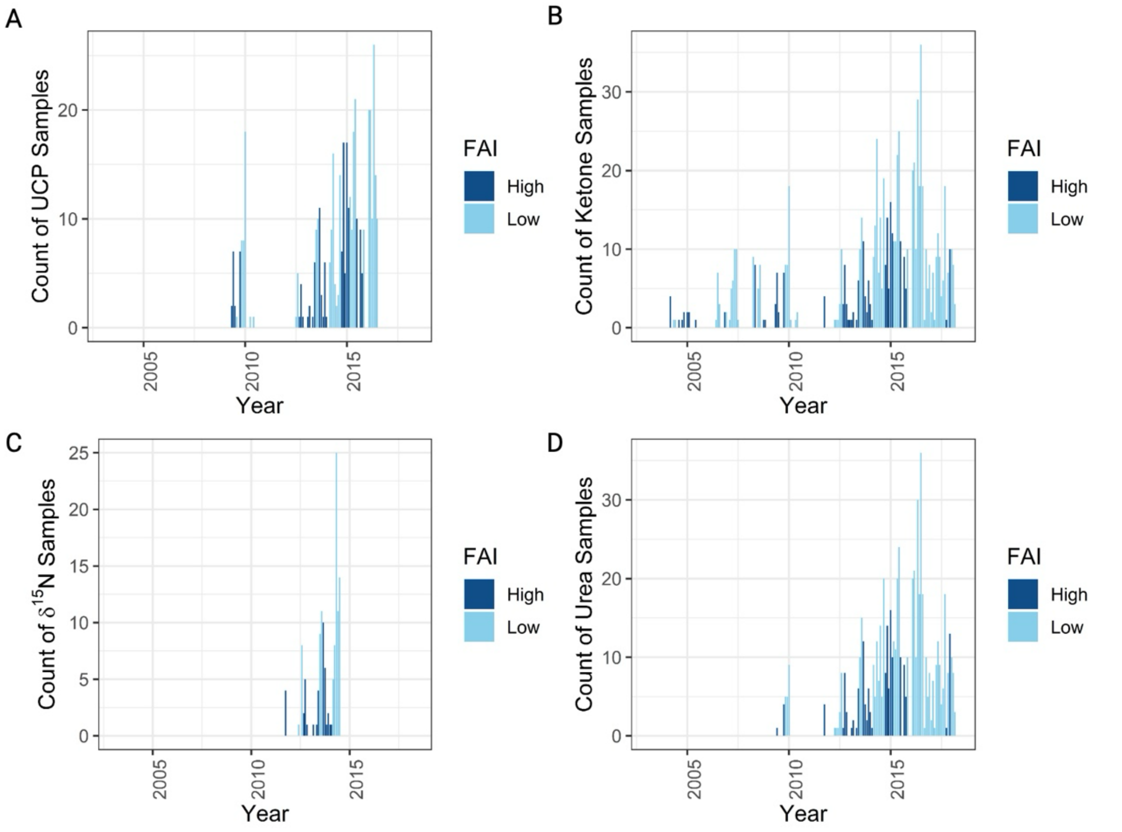

fig. S8. Distribution and count of urine samples used in each of the four analyses that examined the relationship between macronutrient intake and urinary biomarkers of energetic stress. A) ketone body presence, B) C-peptide of insulin (pg/ml), C) urea concentration (mg/ml), and D) $\delta^{15}N$ (‰). Sampling across period of varying fruit availability (FAI) is also shown with high fruit availability periods in drak blue and low in light blue. FAI categorical periods were determined using 50% statistical quartiles as in (*121*). Created in BioRender. Vogel, E. (2025) https://BioRender.com/l6h2iut.

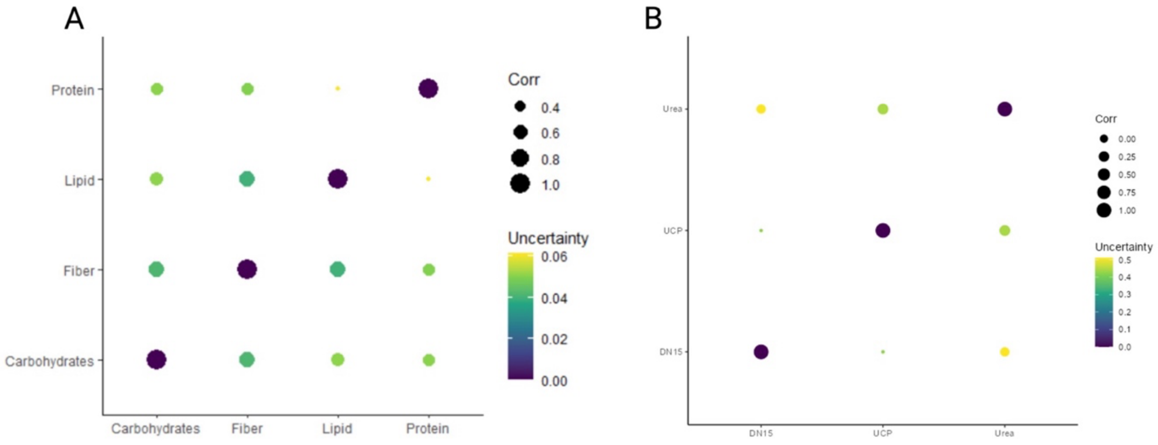

fig. S9. Correlation matrix for daily macronutrient intakes. A) and among urinary biomarkers B) Correlation coefficients were calculated using the BRMS package. The correlation coefficients between biomarkers were as follows: Urea-UCP (r = 0.299); Urea- $\delta^{15}N$ = (r = 0.116); Urinary C-peptide (UCP) and $\delta^{15}N$ (r= -0.202). See SI analysis statistical for details. Carbohydrates = total non-structural carbohydrates; Fiber = neutral detergent fiber.

	**High protein intake**	**Constant or average protein intake**	**Low protein intake**
**High NPe intake**	• Ketone: very low/undetectable<br>• UCP: High<br>• Urea: High – (average/mid if building muscle or growing)<br>• δN^{15}: low	• Ketone: very low; undetectable<br>• UCP: high<br>• Urea: average<br>• δN^{15}: low	• Ketone: very low/undetectable<br>• UCP: high<br>• Urea: low<br>• δN^{15}: low
**Average NPe Intake**	• Ketone: low<br>• UCP: average/mid<br>• Urea: high (average/mid if building muscle or growing)<br>• δN^{15}: low	• Ketone: low<br>• UCP: average/mid<br>• Urea: average<br>• δN^{15}: average/mid	• Ketone: low<br>• UCP: average/mid<br>• Urea: low<br>• δN^{15}: high
**Low NPe intake**	• Ketone: higher<br>• UCP: low<br>• Urea: high (if using body or dietary protein for energy)<br>• δN^{15}: average/mid	• Ketone: higher<br>• UCP: low<br>• Urea: mid (if using dietary protein for energy) or high (if using body protein for energy)<br>• δN^{15}: higher if using body protein for energy	• Ketone: higher<br>• UCP: low<br>• Urea: high (starvation - using body muscle because protein intake is low)<br>• δN^{15}: high (using body muscle)

table S1. The predicted outcomes of the four biomarkers of energetic condition based on variation in protein and non-protein energy intakes (NPe). UCP = urinary C-peptide.

Fruiting Period Category

	Overall	Low	High	% difference*	median	mode
**P (Kcal/day)**	255.55 ± 137.98	259.47 ± 139.14	249.67 ± 136.01	3.85	232.22	136, 192
Lipid (Kcal/day)	453.09 ± 474.67	414.33 ± 478.65	509.61 ± 461.39	20.62	289.13	143
TNC (Kcal/day)	1556.79 ± 1108.79	1412.4 ± 1157.83	1773.28 ± 990.96	22.66	1304.65	887
NDF (Kcal/day)	255.78 ± 160.42	236.87 ± 157.61	284.04 ± 160.41	18.11	221.91	135, 145
NPe (Kcal/day)	2265.66 ± 1480.37	2063.6 ± 1529.25	2566.93 ± 1345.58	21.74	2015.87	631, 795, 1004, 1488, 1494, 1863, 1988, 2844
Total caloric intake (Kcal/day)	2521.21 ± 1554.21	2323.07 ± 1605.59	2816.61 ± 1420.28	19.21	2259.85	842, 1773, 2213
NPe:P (Kcal/day)	9.71 ± 5.74	8.47 ± 5.4	11.56 ± 5.75	30.85	8.85	3.21
Lipid:P (Kcal/day)	1.9 ± 1.71	1.66 ± 1.62	2.24 ± 1.76	29.74	1.35	0.39
Lipid:TNC (Kcal/day)	0.32 ± 0.3	0.32 ± 0.31	0.33 ± 0.29	3.08	0.21	0.18
Lipid:NDF(Kcal/day)	1.85 ± 10.48	1.87 ± 13.5	1.81 ± 1.18	3.26	1.36	0.91
TNC:P (Kcal/day)	6.69 ± 4.37	5.8 ± 4.1	8.03 ± 4.43	32.25	5.72	2.24, 2.3
TNC:NDF (Kcal/day)	6.82 ± 6.07	6.62 ± 7.1	7.12 ± 4.05	7.28	5.76	5.38
P:NDF (Kcal/day)	1.43 ± 11.88	1.69 ± 15.32	1.05 ± 0.66	46.72	1.02	0.7
FAI (% of fruiting trees)	4.5 ± 2.62	2.44 ± 1.08	6.58 ± 1.99	4.14	3.98	4

table S2. Variation in macronutrients and their ratios consumed during full-day focal follows high (n=2047) and low (n=3081) fruit periods. Data represent the mean +/- Standard Deviation. TNC = Total non-structural carbohydrates; P= Available Protein; NDF = Neutral detergent fiber; NPe = Non-protein energy. * % difference = $=|V1-V2|/[(V1+V2)/2]\times 100$.

Model_ID	Covariate	Estimate	Est.Error	l.95..CI	u.95..CI	Rhat	Bulk_ESS	Tail_ESS
**Total kcal ~ FAI**	Intercept	7.62564798	0.0807928	7.45587865	7.7760012	1.0009167	877.357977	1502.50874
Total kcal ~ FAI	class_focaladolescent_male	0.15383786	0.20152652	-0.228887	0.55291687	0.99986663	2021.23283	2214.44459
Total kcal ~ FAI	class_focaladult_female	-0.3517704	0.31676416	-1.0458509	0.25488639	1.00437772	1299.17859	1484.76238
Total kcal ~ FAI	class_focaladult_female_with_dependent	-0.0186232	0.0988291	-0.2116327	0.17755539	1.00475342	1441.05568	1838.29876
Total kcal ~ FAI	class_focaladult_flanged_male	0.06793162	0.09305741	-0.106607	0.26356021	0.99996019	1132.2239	1906.68748
Total kcal ~ FAI	class_focaladult_unflanged_male	0.21865177	0.10521933	0.01374551	0.42727968	1.00219637	1325.91709	2104.56826
Total kcal ~ FAI	class_focalweaned_immature_female	0.08161475	0.13534613	-0.2704819	0.28072617	1.00094898	1761.69463	1337.54902
Total kcal ~ FAI	class_focalweaned_immature_male	-0.0923374	0.16083554	-0.3915948	0.23972581	1.000228	1495.99247	2445.93749
Total kcal ~ FAI	sfai_1	2.56960914	1.15091201	0.25140416	4.74748293	1.00106888	1670.50067	2378.18531
Total kcal ~ FAI	sds(sfai_1)	2.09978009	0.65866483	1.17384697	3.68460418	1.00079992	1159.36517	1734.334
Npe ~ FAI	Intercept	7.51298225	0.08425652	7.33841029	7.66720362	1.00069012	797.351151	1506.49272
Npe ~ FAI	class_focaladolescent_male	0.1620212	0.21172765	-0.2509985	0.58493495	1.00146418	1517.96379	1813.1431
Npe ~ FAI	class_focaladult_female	-0.3403647	0.37388273	-1.0320206	0.38096348	1.00499809	1101.80645	854.664457
Npe ~ FAI	class_focaladult_female_with_dependent	-0.0192792	0.09777903	-0.2176672	0.17730084	1.0021953	1160.95744	1599.80057
Npe ~ FAI	class_focaladult_flanged_male	0.04577582	0.09645587	-0.1361714	0.24421467	1.00093584	1108.94675	1653.31551
Npe ~ FAI	class_focaladult_unflanged_male	0.21870436	0.10744279	0.00988783	0.43222826	1.000612	1178.47978	2300.51573
Npe ~ FAI	class_focalweaned_immature_female	0.10037397	0.14830033	-0.23327	0.32274883	0.9999092	1464.10809	1173.17661
Npe ~ FAI	class_focalweaned_immature_male	-0.1314766	0.16308029	-0.4411505	0.19634424	1.00092595	1492.15383	2135.76093
Npe ~ FAI	sfai_1	3.07011807	1.17952977	0.72719799	5.36741991	1.00055893	2491.69084	2322.9848
Npe ~ FAI	sds(sfai_1)	2.35316313	0.7434083	1.30587409	4.22742796	1.00341146	1024.7083	1747.08577
Protein ~ FAI	Intercept	5.39516308	0.07907933	5.2291694	5.5398023	1.00435349	940.241066	1458.33
Protein ~ FAI	class_focaladolescent_male	0.11182941	0.16951614	-0.2116391	0.45811149	1.00044713	1639.62357	2531.08841
Protein ~ FAI	class_focaladult_female	-0.3153853	0.2712482	-0.8888538	0.22758269	1.00193933	2232.35313	1754.28336
Protein ~ FAI	class_focaladult_female_with_dependent	0.00568677	0.10474982	-0.1980967	0.21037463	1.00118488	1249.21561	1941.9498
Protein ~ FAI	class_focaladult_flanged_male	0.17590172	0.08832006	0.01007502	0.35593922	1.00417207	1026.51255	1774.34559
Protein ~ FAI	class_focaladult_unflanged_male	0.1710081	0.10918556	-0.0426104	0.38578486	1.00227661	1281.59271	1893.80336
Protein ~ FAI	class_focalweaned_immature_female	0.01928786	0.16407921	-0.3139565	0.28495344	1.00100746	1570.56045	1362.14188
Protein ~ FAI	class_focalweaned_immature_male	0.1230498	0.18562898	-0.2288886	0.50283117	1.0026544	1749.95064	2500.14325
Protein ~ FAI	sfai_1	0.31675237	0.55912213	-0.7679184	1.51357641	1.00017227	2837.44359	2797.05772
Protein ~ FAI	sds(sfai_1)	0.3701081	0.20015814	0.13261437	0.88423039	1.00094257	1801.58648	2890.81129
Lipid ~ FAI	Intercept	5.89300202	0.10021918	5.67681688	6.08447611	1.00052012	1516.5039	2122.1197
Lipid ~ FAI	class_focaladolescent_male	0.0430525	0.24593729	-0.4333589	0.52961631	1.00029623	2699.0362	2757.28073
Lipid ~ FAI	class_focaladult_female	-0.2159426	0.34181928	-0.9225987	0.47083959	1.00202812	1927.89293	1529.44095
Lipid ~ FAI	class_focaladult_female_with_dependent	0.00656042	0.13468001	-0.2500182	0.27796343	1.00011303	1725.50922	2004.16726

Lipid ~ FAI	class_focaladult_flanged_male	-0.1151521	0.1195722	-0.349374	0.12109354	1.00021582	1856.6659	2309.02304
Lipid ~ FAI	class_focaladult_unflanged_male	0.1292609	0.13100741	-0.1310549	0.3974134	1.00027088	1884.05303	2537.24787
Lipid ~ FAI	class_focalweaned_immature_female	0.22877118	0.1519541	-0.1041446	0.50279517	1.00042561	2816.15249	1922.08499
Lipid ~ FAI	class_focalweaned_immature_male	-0.270136	0.20831175	-0.6733341	0.15528108	1.00029444	2579.56352	2745.93229
Lipid ~ FAI	sfai_1	5.30956904	1.69611276	1.90252567	8.60649975	1.00120336	3355.41658	2651.73242
Lipid ~ FAI	sds(sfai_1)	3.40377509	0.93361118	2.02444296	5.64219048	1.00184969	1593.97224	2071.98444
TNC ~ FAI	Intercept	7.10894829	0.0951051	6.90804574	7.28864702	1.00191978	1800.01239	2375.8958
TNC ~ FAI	class_focaladolescent_male	0.23026425	0.24556009	-0.2154525	0.70164737	1.00063154	3260.74265	2646.16158
TNC ~ FAI	class_focaladult_female	-0.3769463	0.38121793	-1.1240011	0.34939028	1.00142806	3062.80104	2104.14473
TNC ~ FAI	class_focaladult_female_with_dependent	-0.0184861	0.10529377	-0.2293429	0.18713828	1.00136373	2713.14352	2521.80624
TNC ~ FAI	class_focaladult_flanged_male	0.08376286	0.10872204	-0.1231628	0.30922326	1.00139458	1985.35102	2709.95019
TNC ~ FAI	class_focaladult_unflanged_male	0.25641321	0.12910206	-0.0034867	0.51752072	1.00005585	2253.76094	2635.80492
TNC ~ FAI	class_focalweaned_immature_female	0.02757554	0.21083878	-0.5196033	0.33674419	1.0003796	2485.73132	1696.79279
TNC ~ FAI	class_focalweaned_immature_male	-0.1213563	0.18278002	-0.4476982	0.26337959	1.00131731	2121.46887	2502.51671
TNC ~ FAI	sfai_1	2.8309147	1.25715541	0.34077412	5.23634853	1.0002437	4534.0112	3018.37336
TNC ~ FAI	sds(sfai_1)	2.27634188	0.70813885	1.27874337	3.99013929	1.00166169	2067.76783	2301.45183
NDF ~ FAI	Intercept	5.4225699	0.07527833	5.26541316	5.55968619	1.00129127	1180.30249	1606.30039
NDF ~ FAI	class_focaladolescent_male	0.13385549	0.19716103	-0.253843	0.54358395	1.00197001	2052.42933	2368.73735
NDF ~ FAI	class_focaladult_female	-0.357857	0.3456482	-1.1240568	0.31128371	1.00046895	1731.61937	1610.31216
NDF ~ FAI	class_focaladult_female_with_dependent	-0.0908705	0.10099791	-0.2924679	0.11043453	1.0009994	1476.04494	2335.89209
NDF ~ FAI	class_focaladult_flanged_male	0.00601512	0.08314387	-0.1518294	0.17786415	1.00071773	1403.04486	1715.59015
NDF ~ FAI	class_focaladult_unflanged_male	0.09156228	0.09601861	-0.0960939	0.28558805	1.0007683	1508.17578	2052.61944
NDF ~ FAI	class_focalweaned_immature_female	-0.0243923	0.26528078	-0.580757	0.48162217	1.00034779	2605.3071	2200.04629
NDF ~ FAI	class_focalweaned_immature_male	-0.1003311	0.1506989	-0.3813569	0.21858445	1.00007762	1769.26716	2372.99311
NDF ~ FAI	sfai_1	1.23666289	1.20493837	-1.1743704	3.46244339	1.00029316	3011.81486	3142.83376
NDF ~ FAI	sds(sfai_1)	1.8799428	0.61126778	1.00102606	3.38632378	1.00114256	1454.81075	2184.58531
Npe:P ~ FAI	Intercept	2.19339134	0.04820551	2.09508397	2.28579344	1.00301615	943.944983	1514.73899
Npe:P ~ FAI	class_focaladolescent_male	0.02938309	0.14978545	-0.2633444	0.30495884	1.00112199	1829.46263	1906.13708
Npe:P ~ FAI	class_focaladult_female	-0.058915	0.14986827	-0.3590736	0.24530384	1.0026286	1574.26082	1603.00209
Npe:P ~ FAI	class_focaladult_female_with_dependent	0.00553663	0.05038048	-0.0905335	0.11039029	1.00133942	1355.74768	1155.86915
Npe:P ~ FAI	class_focaladult_flanged_male	-0.1292363	0.06007509	-0.2467074	-0.0092949	1.00108804	1140.50769	1834.35597
Npe:P ~ FAI	class_focaladult_unflanged_male	0.00848261	0.06769971	-0.121191	0.14412346	1.00159017	1375.2435	1914.08896
Npe:P ~ FAI	class_focalweaned_immature_female	0.08706107	0.10072715	-0.1271969	0.28482419	1.00279955	2074.8103	1907.53125
Npe:P ~ FAI	class_focalweaned_immature_male	-0.2290733	0.09830422	-0.4278331	-0.0348724	1.00138461	1462.91103	2222.77511
Npe:P ~ FAI	sfai_1	2.73784202	1.01430681	0.72280786	4.66541787	0.99971551	2035.10826	2416.87248
Npe:P ~ FAI	sds(sfai_1)	1.94637198	0.56685206	1.14458011	3.29195136	1.00020875	1023.59234	1874.7431
Lipid:P ~ FAI	Intercept	0.53592657	0.07206785	0.38535143	0.67314037	1.00032211	774.073337	1014.27032
Lipid:P ~ FAI	class_focaladolescent_male	-0.0440888	0.19691085	-0.4387758	0.33277754	1.00019788	1596.28764	1966.19504
Lipid:P ~ FAI	class_focaladult_female	0.05916763	0.41848902	-0.7860349	0.88714825	1.0018035	1402.01736	1247.46381

Lipid:P ~ FAI	class_focaladult_female_with_dependent	0.00869777	0.07586779	-0.1304798	0.17797158	1.00045533	895.076807	1030.2764
Lipid:P ~ FAI	class_focaladult_flanged_male	-0.2409298	0.09477243	-0.4319788	-0.0491663	1.00155725	854.528293	1288.78381
Lipid:P ~ FAI	class_focaladult_unflanged_male	-0.0105538	0.11053719	-0.2348694	0.20481935	1.00091945	1106.89513	2114.83488
Lipid:P ~ FAI	class_focalweaned_immature_female	0.20762753	0.1420621	-0.0983681	0.46047078	1.00096938	1515.56516	1499.84117
Lipid:P ~ FAI	class_focalweaned_immature_male	-0.3566121	0.14523881	-0.6525456	-0.0816717	1.00078737	1153.11594	2097.03621
Lipid:P ~ FAI	sfai_1	4.73471565	1.64854412	1.34946547	7.94033508	1.00031348	2216.92363	2165.94385
Lipid:P ~ FAI	sds(sfai_1)	3.44414638	0.94163263	2.03136684	5.69139138	1.0011063	958.095767	1763.68889
Lipid:TNC ~ FAI	Intercept	-1.2695089	0.06827884	-1.4006953	-1.1266202	1.00247256	1183.07083	1238.21019
Lipid:TNC ~ FAI	class_focaladolescent_male	-0.0953878	0.21175792	-0.5214588	0.32322279	1.00196472	2569.41685	2378.5512
Lipid:TNC ~ FAI	class_focaladult_female	0.30730277	0.28388719	-0.265009	0.87751159	1.0007934	1435.19153	1301.32494
Lipid:TNC ~ FAI	class_focaladult_female_with_dependent	0.12020148	0.09031147	-0.0497837	0.30133747	1.00114384	1534.44519	2414.91194
Lipid:TNC ~ FAI	class_focaladult_flanged_male	-0.0734065	0.08982582	-0.256269	0.09712027	1.00142296	1424.65528	1546.80174
Lipid:TNC ~ FAI	class_focaladult_unflanged_male	0.0040275	0.11881963	-0.2411124	0.23263312	1.00066018	2129.64529	2240.79476
Lipid:TNC ~ FAI	class_focalweaned_immature_female	0.16766732	0.20789099	-0.2679949	0.57787915	1.00036792	2308.70435	2131.28556
Lipid:TNC ~ FAI	class_focalweaned_immature_male	0.00400988	0.13433114	-0.2857559	0.26054361	1.00391118	1278.78907	2045.06985
Lipid:TNC ~ FAI	sfai_1	2.06001973	1.54509367	-0.9130038	5.16082057	1.00161073	3363.13162	2864.09832
Lipid:TNC ~ FAI	sds(sfai_1)	3.49631864	0.98150961	2.08134687	5.86212815	1.00073573	1209.11769	1836.94847
Lipid:NDF ~ FAI	Intercept	0.49560689	0.07534659	0.3464596	0.63858015	1.00115088	1068.4909	1876.33713
Lipid:NDF ~ FAI	class_focaladolescent_male	-0.0595864	0.18877554	-0.4283917	0.31417011	1.00096087	2353.16254	2177.81044
Lipid:NDF ~ FAI	class_focaladult_female	0.00378334	0.25251827	-0.4869362	0.42401171	1.00054482	1560.10249	1157.12502
Lipid:NDF ~ FAI	class_focaladult_female_with_dependent	0.06582096	0.08812204	-0.1005107	0.24948292	1.00029147	1249.06239	1841.73338
Lipid:NDF ~ FAI	class_focaladult_flanged_male	-0.1595165	0.08738807	-0.3288051	0.01496272	1.00156774	1157.42797	2241.28184
Lipid:NDF ~ FAI	class_focaladult_unflanged_male	0.02322904	0.09790369	-0.1639388	0.21412102	1.00176555	1268.08747	2413.32543
Lipid:NDF ~ FAI	class_focalweaned_immature_female	0.22463825	0.16837844	-0.1063354	0.54170639	1.00023214	2393.38361	2274.02572
Lipid:NDF ~ FAI	class_focalweaned_immature_male	-0.1093978	0.25689633	-0.6288518	0.41364374	0.999923	2231.54235	2561.89879
Lipid:NDF ~ FAI	sfai_1	11.6160491	1.51670407	8.65505297	14.6291677	1.00099495	3234.35729	2985.25905
Lipid:NDF ~ FAI	sds(sfai_1)	4.84872858	1.25875403	2.97069988	7.91466697	1.00014526	1153.53492	2142.93837
TNC:P ~ FAI	Intercept	1.81276668	0.05506903	1.70314171	1.91838422	1.00064294	1926.27223	1988.19963
TNC:P ~ FAI	class_focaladolescent_male	0.04131749	0.17620521	-0.2978529	0.40562356	0.99996829	3129.3608	2645.41071
TNC:P ~ FAI	class_focaladult_female	-0.1109344	0.15185358	-0.4201283	0.17134933	1.00033841	2566.95982	1983.26167
TNC:P ~ FAI	class_focaladult_female_with_dependent	5.59E-05	0.05717007	-0.113038	0.1153178	1.00038678	2346.66698	2453.61937
TNC:P ~ FAI	class_focaladult_flanged_male	-0.1077146	0.06700559	-0.2351122	0.02318137	1.00025374	2191.26058	2705.40965
TNC:P ~ FAI	class_focaladult_unflanged_male	0.01353966	0.07463994	-0.1336926	0.16348451	1.0001106	2342.49909	2542.34464
TNC:P ~ FAI	class_focalweaned_immature_female	0.03838092	0.15998036	-0.3270551	0.32039364	1.0005701	2781.24821	2107.15326
TNC:P ~ FAI	class_focalweaned_immature_male	-0.2240664	0.11301861	-0.4386438	0.0027471	1.00088709	2578.48743	2670.94641
TNC:P ~ FAI	sfai_1	2.89670987	1.07844957	0.80336529	5.0113205	1.00074884	3811.74657	2847.84617
TNC:P ~ FAI	sds(sfai_1)	1.96893734	0.59317112	1.13067749	3.40001812	1.00202936	1420.38867	2460.68655
TNC:NDF ~ FAI	Intercept	1.82896796	0.05037127	1.7232882	1.92553606	1.00047412	778.571126	1139.9706
TNC:NDF ~ FAI	class_focaladolescent_male	0.07880281	0.13998066	-0.1973308	0.36103545	1.00011454	1879.82131	2356.96178

TNC:NDF ~ FAI	class_focaladult_female	-0.058943	0.28183969	-0.6265876	0.52317316	1.0018441	1443.01839	1699.14481
TNC:NDF ~ FAI	class_focaladult_female_with_dependent	0.07693138	0.05208706	-0.0255943	0.17992427	0.99996744	1328.5875	1686.88698
TNC:NDF ~ FAI	class_focaladult_flanged_male	0.00708231	0.069099	-0.1244568	0.14806469	1.00103721	940.143272	1436.44701
TNC:NDF ~ FAI	class_focaladult_unflanged_male	0.12748157	0.09397901	-0.0600446	0.31685215	1.00000645	1342.49458	2091.58176
TNC:NDF ~ FAI	class_focalweaned_immature_female	0.05088787	0.21483412	-0.3651052	0.49109775	1.00200691	1147.96136	1354.49627
TNC:NDF ~ FAI	class_focalweaned_immature_male	-0.0151268	0.15439557	-0.32913	0.27111876	1.00045094	1199.9905	2033.98573
TNC:NDF ~ FAI	sfai_1	2.94550768	1.11202851	0.89488254	5.19623918	1.00067191	1560.46851	2357.25812
TNC:NDF ~ FAI	sds(sfai_1)	1.35006156	0.48536234	0.71215864	2.52803833	1.00016474	1176.53551	2048.42699
P:NDF ~ FAI	Intercept	0.21196596	0.06770038	0.07620885	0.34030646	1.00176667	947.567085	1619.20911
P:NDF ~ FAI	class_focaladolescent_male	-0.0198993	0.16882195	-0.3511118	0.31716574	1.00091994	1880.67952	1841.81083
P:NDF ~ FAI	class_focaladult_female	-0.0664261	0.29979019	-0.6889625	0.5582868	1.00017497	1676.33897	1624.02492
P:NDF ~ FAI	class_focaladult_female_with_dependent	0.06602271	0.09050609	-0.1098376	0.24935339	1.00082586	1245.32768	2014.57347
P:NDF ~ FAI	class_focaladult_flanged_male	0.06859228	0.07636997	-0.0819706	0.21537067	1.00122967	1050.26702	1940.00583
P:NDF ~ FAI	class_focaladult_unflanged_male	0.01627701	0.09235779	-0.1639142	0.20387699	1.00284272	1141.4037	1745.96511
P:NDF ~ FAI	class_focalweaned_immature_female	-0.0015433	0.17406958	-0.3162208	0.38722482	1.00249107	1490.64356	1009.74569
P:NDF ~ FAI	class_focalweaned_immature_male	0.19686895	0.28152967	-0.359837	0.75873333	1.00020569	1742.29581	2185.47378
P:NDF ~ FAI	sfai_1	9.98035827	1.44842368	7.16495395	12.8918849	1.00060596	2938.00411	2779.02715
P:NDF ~ FAI	sds(sfai_1)	5.76580773	1.45918445	3.58716423	9.25910689	1.00320927	882.026048	1446.47132

table S3. Results of all models examining nutritional intake variables and the fruit availability index (FAI). All nutritional intakes are in kcal/day and FAI is the percentage of fruiting trees. All models include individual orangutan ID as a random effect in the model.

Group-Level Effects:							
~name_focal (Number of levels: 112)							
	Estimate	Est.Error	l-95% CI	u-95% CI	Rhat	Bulk_ESS	Tail_ESS
sd(b1_Intercept)	2696.84	583.05	1652.52	3845.48	1	415	1267
sd(a_Intercept)	0.1	0.02	0.07	0.13	1	1207	2086

Population-Level Effects:							
	Estimate	Est.Error	l-95% CI	u-95% CI	Rhat	Bulk_ESS	Tail_ESS
b1_Intercept	12715.08	639.07	11516.3	13993.53	1	2145	2240
a_Intercept	-0.73	0.02	-0.77	-0.69	1	1854	2676

Family Specific Parameters:							
	Estimate	Est.Error	l-95% CI	u-95% CI	Rhat	Bulk_ESS	Tail_ESS
shape	3.98	0.08	3.83	4.13	1	4540	2331

Whole Model Summary				
	Estimate	Est.Error	Q2.5	Q97.5
R^2	0.38502	0.00988	0.36543	0.40406

table S4. Bayesian analysis testing for the strength of protein leverage following (*60*). The relationship between total daily caloric intake and percent of protein intake was modeled as a power function. Draws were sampled using sampling(NUTS). For each parameter, Bulk_ESS and Tail_ESS are effective sample size measures, and Rhat is the potential scale reduction factor on split chains (at convergence, Rhat = 1). The model details are found in Supplementary Materials Statistical Analysis Equation 3 and fig. S1.

Model_ID	Covariate	Estimate	Est.Error	l.95..CI	u.95..CI	Rhat	Bulk_ESS	Tail_ESS
**C-peptide ~ FAI**	Intercept	7.64	0.06	7.53	7.75	1.00	3650.11	3102.70
C-peptide ~ FAI	sfai_1	2.50	3.14	-3.60	9.26	1.00	2165.83	2594.27
C-peptide ~ FAI	sucp_time_collected_1	-1.61	1.80	-5.44	2.13	1.00	1893.54	1755.92
C-peptide ~ FAI	sds(sfai_1)	2.89	1.82	0.51	7.22	1.00	737.88	1216.08
C-peptide ~ FAI	sds(sucp_time_collected_1)	0.69	0.66	0.03	2.41	1.00	1576.48	2271.30
d15N ~ FAI	Intercept	-1.12	0.12	-1.36	-0.88	1.00	3424.50	3097.79
d15N ~ FAI	sfai_1	3.20	3.96	-2.40	12.57	1.00	1079.99	2207.18
d15N ~ FAI	sisotope_time_collected_1	2.15	2.90	-4.08	7.72	1.00	2568.93	2270.08
d15N ~ FAI	sds(sfai_1)	2.33	1.70	0.09	6.27	1.01	694.47	1027.49
d15N ~ FAI	sds(sisotope_time_collected_1)	1.26	1.02	0.06	3.84	1.00	2162.04	1993.14
Ketone presence~ FAI	Intercept	-2.92	0.28	-3.53	-2.42	1.00	4137.71	3921.63
Ketone presence~ FAI	sfai_1	-0.28	1.47	-3.41	2.44	1.00	8408.91	4450.90
Ketone presence~ FAI	sds(sfai_1)	1.45	1.51	0.04	5.42	1.00	2207.46	3363.02
Urea ~ FAI	Intercept	6.65	0.05	6.55	6.76	1.00	8213.17	10948.16
Urea ~ FAI	sfai_1	-0.73	3.35	-7.16	6.00	1.00	7945.23	9273.96
Urea ~ FAI	sds(sfai_1)	5.30	1.68	2.77	9.15	1.00	4405.40	6982.63

table S5. Results of models examining relationships between urinary metabolites and the fruit availability index (FAI). All models included age-sex class as a fixed effect and orangutan ID as a random effect (n=4873 full day follows). All models conformed to a gamma distribution. (Ketone presence n=1115; C-peptide n=580; urea n=924; δN^{15} n=195).

Model_ID	Covariate	Estimate	Est.Error	l.95..CI	u.95..CI	Rhat	Bulk_ESS	Tail_ESS
**Ketone presence~ Total kcal**	Intercept	-3.33	0.58	-4.53	-2.24	1.00	4158.26	3965.13
Ketone presence~ Total kcal	class_focaladolescent_male	-0.54	1.64	-4.24	2.30	1.00	8408.40	3120.22
Ketone presence~ Total kcal	class_focaladult_female	0.78	1.17	-1.65	3.03	1.00	10459.70	3802.61
Ketone presence~ Total kcal	class_focaladult_female_with_dependent	0.74	0.59	-0.41	1.94	1.00	5074.72	4376.83
Ketone presence~ Total kcal	class_focaladult_flanged_male	0.36	0.67	-0.96	1.68	1.00	4027.59	4483.08
Ketone presence~ Total kcal	class_focaladult_unflanged_male	1.07	0.87	-0.60	2.84	1.00	4825.34	4326.92
Ketone presence~ Total kcal	class_focalweaned_immature_female	-0.93	1.62	-4.97	1.59	1.00	6967.35	3248.88
Ketone presence~ Total kcal	class_focalweaned_immature_male	0.58	1.00	-1.43	2.55	1.00	6342.20	4801.69
Ketone presence~ Total kcal	sfai_1	-0.28	1.42	-3.28	2.41	1.00	7165.26	3749.83
Ketone presence~ Total kcal	sprev_day_total_kcal_using_ap_low_fermentation_1	-0.16	1.63	-3.55	2.99	1.00	8396.71	4014.27
Ketone presence~ Total kcal	sds(sfai_1)	0.86	0.85	0.03	3.14	1.00	3594.26	3774.36
Ketone presence~ Total kcal	sds(sprev_day_total_kcal_using_ap_low_fermentation_1)	0.97	0.88	0.03	3.17	1.00	5355.74	3436.76
Ketone presence~ Protein	Intercept	-3.30	0.57	-4.49	-2.25	1.00	4927.29	4183.72
Ketone presence~ Protein	class_focaladolescent_male	-0.58	1.74	-4.53	2.36	1.00	7408.32	3242.77
Ketone presence~ Protein	class_focaladult_female	0.80	1.14	-1.49	2.94	1.00	10303.53	4154.00
Ketone presence~ Protein	class_focaladult_female_with_dependent	0.74	0.58	-0.37	1.94	1.00	5905.72	4300.49
Ketone presence~ Protein	class_focaladult_flanged_male	0.22	0.69	-1.13	1.58	1.00	4400.21	4078.12
Ketone presence~ Protein	class_focaladult_unflanged_male	1.02	0.87	-0.65	2.82	1.00	5769.14	4827.80
Ketone presence~ Protein	class_focalweaned_immature_female	-0.94	1.63	-4.65	1.56	1.00	7577.42	3076.22
Ketone presence~ Protein	class_focalweaned_immature_male	0.60	1.01	-1.36	2.60	1.00	7217.78	4410.13
Ketone presence~ Protein	sfai_1	-0.35	1.38	-3.22	2.28	1.00	8561.24	4281.53
Ketone presence~ Protein	sprev_day_ap_kcal_1	0.35	1.48	-2.63	3.25	1.00	5541.37	3875.91
Ketone presence~ Protein	sds(sfai_1)	0.88	0.86	0.03	3.01	1.00	3374.63	3711.67
Ketone presence~ Protein	sds(sprev_day_ap_kcal_1)	0.76	0.67	0.03	2.46	1.00	4541.50	3556.52
Ketone presence~ Lipid	Intercept	-3.37	0.57	-4.56	-2.32	1.00	3675.72	3723.39
Ketone presence~ Lipid	class_focaladolescent_male	-0.56	1.75	-4.32	2.33	1.00	5700.30	2604.61
Ketone presence~ Lipid	class_focaladult_female	0.81	1.17	-1.52	3.08	1.00	8646.58	3985.90
Ketone presence~ Lipid	class_focaladult_female_with_dependent	0.77	0.59	-0.35	1.97	1.00	4066.24	4215.86
Ketone presence~ Lipid	class_focaladult_flanged_male	0.33	0.68	-1.00	1.65	1.00	3463.60	3897.80
Ketone presence~ Lipid	class_focaladult_unflanged_male	1.10	0.88	-0.59	2.82	1.00	4016.09	4351.37
Ketone presence~ Lipid	class_focalweaned_immature_female	-0.99	1.78	-5.10	1.61	1.00	4011.51	1844.00
Ketone presence~ Lipid	class_focalweaned_immature_male	0.57	1.03	-1.48	2.59	1.00	5306.46	3426.51
Ketone presence~ Lipid	sfai_1	-0.21	1.41	-3.16	2.58	1.00	6012.14	3942.84
Ketone presence~ Lipid	sprev_day_lipid_kcal_1	-1.06	1.81	-5.07	2.15	1.00	6467.49	3353.76
Ketone presence~ Lipid	sds(sfai_1)	0.83	0.80	0.03	2.97	1.00	3898.44	3701.40
Ketone presence~ Lipid	sds(sprev_day_lipid_kcal_1)	1.09	0.98	0.03	3.65	1.00	4004.94	2777.23
Ketone presence~ TNC	Intercept	-3.33	0.57	-4.52	-2.28	1.00	5272.33	4460.24
Ketone presence~ TNC	class_focaladolescent_male	-0.54	1.72	-4.55	2.36	1.00	8076.29	2849.93

Ketone presence~ TNC	class_focaladult_female	0.80	1.14	-1.45	3.02	1.00	11214.21	4356.13
Ketone presence~ TNC	class_focaladult_female_with_dependent	0.72	0.59	-0.41	1.91	1.00	5901.21	4527.51
Ketone presence~ TNC	class_focaladult_flanged_male	0.38	0.69	-0.97	1.74	1.00	5747.50	5226.04
Ketone presence~ TNC	class_focaladult_unflanged_male	1.05	0.87	-0.65	2.73	1.00	7299.90	4679.66
Ketone presence~ TNC	class_focalweaned_immature_female	-0.92	1.69	-4.88	1.76	1.00	7678.87	2764.46
Ketone presence~ TNC	class_focalweaned_immature_male	0.61	0.99	-1.36	2.52	1.00	8562.37	5512.79
Ketone presence~ TNC	sfai_1	-0.32	1.45	-3.41	2.45	1.00	8503.32	3766.00
Ketone presence~ TNC	sprev_day_tnc_kcal_1	0.05	1.86	-3.75	3.73	1.00	10069.86	3669.31
Ketone presence~ TNC	sds(sfai_1)	0.90	0.91	0.03	3.24	1.00	3802.57	3434.01
Ketone presence~ TNC	sds(sprev_day_tnc_kcal_1)	1.13	1.01	0.04	3.64	1.00	5361.87	2889.04
Ketone presence~ NDF	Intercept	-3.40	0.60	-4.64	-2.27	1.00	4867.45	4181.13
Ketone presence~ NDF	class_focaladolescent_male	-0.56	1.71	-4.47	2.42	1.00	9186.46	3120.66
Ketone presence~ NDF	class_focaladult_female	0.84	1.16	-1.49	3.12	1.00	10057.34	4592.26
Ketone presence~ NDF	class_focaladult_female_with_dependent	0.84	0.62	-0.35	2.07	1.00	5632.24	4470.25
Ketone presence~ NDF	class_focaladult_flanged_male	0.15	0.74	-1.36	1.59	1.00	4261.63	4646.83
Ketone presence~ NDF	class_focaladult_unflanged_male	1.00	0.92	-0.76	2.87	1.00	6103.75	4650.21
Ketone presence~ NDF	class_focalweaned_immature_female	-1.03	2.12	-5.48	1.74	1.00	4806.35	2171.86
Ketone presence~ NDF	class_focalweaned_immature_male	0.55	1.04	-1.52	2.59	1.00	6504.69	4708.56
Ketone presence~ NDF	sfai_1	-0.26	1.44	-3.17	2.48	1.00	7775.59	3921.69
Ketone presence~ NDF	sprev_day_ndf_kcal_low_1	-0.13	1.63	-3.46	2.99	1.00	8692.35	3681.11
Ketone presence~ NDF	sds(sfai_1)	0.87	0.87	0.03	3.18	1.00	3883.90	3960.50
Ketone presence~ NDF	sds(sprev_day_ndf_kcal_low_1)	1.34	1.30	0.03	4.79	1.00	3141.71	2977.83
Ketone presence~ Npe	Intercept	-3.35	0.59	-4.56	-2.24	1.00	5446.44	4438.96
Ketone presence~ Npe	class_focaladolescent_male	-0.54	1.72	-4.41	2.39	1.00	9171.24	3061.27
Ketone presence~ Npe	class_focaladult_female	0.80	1.16	-1.49	3.05	1.00	11424.90	4433.10
Ketone presence~ Npe	class_focaladult_female_with_dependent	0.74	0.61	-0.43	2.01	1.00	6559.47	4452.81
Ketone presence~ Npe	class_focaladult_flanged_male	0.38	0.68	-0.95	1.75	1.00	5434.50	4553.84
Ketone presence~ Npe	class_focaladult_unflanged_male	1.08	0.87	-0.56	2.83	1.00	6059.20	4796.07
Ketone presence~ Npe	class_focalweaned_immature_female	-0.91	1.61	-4.63	1.62	1.00	7523.89	3545.61
Ketone presence~ Npe	class_focalweaned_immature_male	0.61	1.00	-1.30	2.55	1.00	7297.57	4576.68
Ketone presence~ Npe	sfai_1	-0.25	1.41	-3.21	2.49	1.00	8207.54	3887.06
Ketone presence~ Npe	sprev_day_total_kcal_npe_low_fermentation_1	-0.25	1.67	-3.78	2.99	1.00	10138.41	3478.31
Ketone presence~ Npe	sds(sfai_1)	0.88	0.88	0.03	3.21	1.00	3584.54	3729.27
Ketone presence~ Npe	sds(sprev_day_total_kcal_npe_low_fermentation_1)	0.97	0.88	0.03	3.12	1.00	4238.84	3109.63
Ketone presence~ Npe:P	Intercept	-3.35	0.59	-4.53	-2.24	1.00	3807.13	3897.67
Ketone presence~ Npe:P	class_focaladolescent_male	-0.54	1.72	-4.41	2.39	1.00	9181.93	2945.00
Ketone presence~ Npe:P	class_focaladult_female	0.78	1.17	-1.62	3.01	1.00	10327.36	4097.18
Ketone presence~ Npe:P	class_focaladult_female_with_dependent	0.71	0.59	-0.43	1.91	1.00	4343.22	3971.24
Ketone presence~ Npe:P	class_focaladult_flanged_male	0.32	0.68	-1.02	1.67	1.00	3519.09	3543.82

Ketone presence~ Npe:P	class_focaladult_unflanged_male	1.08	0.88	-0.69	2.81	1.00	4634.75	3967.35
Ketone presence~ Npe:P	class_focalweaned_immature_female	-0.97	1.69	-4.88	1.59	1.00	6411.68	2487.62
Ketone presence~ Npe:P	class_focalweaned_immature_male	0.54	1.01	-1.50	2.52	1.00	6188.27	4595.95
Ketone presence~ Npe:P	sfai_1	-0.01	1.34	-2.75	2.53	1.00	8504.32	4384.84
Ketone presence~ Npe:P	sprev_day_npe_ab_1	-1.30	1.60	-4.80	1.59	1.00	7087.31	4199.72
Ketone presence~ Npe:P	sds(sfai_1)	0.84	0.84	0.02	2.92	1.00	4174.61	3618.22
Ketone presence~ Npe:P	sds(sprev_day_npe_ab_1)	0.87	0.74	0.03	2.78	1.00	3731.08	3456.69
Ketone presence~ Lipid:P	Intercept	-3.39	0.57	-4.56	-2.32	1.00	4623.07	4070.52
Ketone presence~ Lipid:P	class_focaladolescent_male	-0.55	1.73	-4.37	2.51	1.00	6681.18	2737.93
Ketone presence~ Lipid:P	class_focaladult_female	0.76	1.17	-1.54	3.05	1.00	10089.49	4623.20
Ketone presence~ Lipid:P	class_focaladult_female_with_dependent	0.73	0.59	-0.39	1.90	1.00	4709.67	4062.74
Ketone presence~ Lipid:P	class_focaladult_flanged_male	0.32	0.68	-1.02	1.64	1.00	4753.30	4960.59
Ketone presence~ Lipid:P	class_focaladult_unflanged_male	1.07	0.86	-0.65	2.76	1.00	5332.48	4468.56
Ketone presence~ Lipid:P	class_focalweaned_immature_female	-1.01	1.70	-5.13	1.68	1.00	6007.86	2252.91
Ketone presence~ Lipid:P	class_focalweaned_immature_male	0.54	1.00	-1.40	2.49	1.00	6102.45	4508.99
Ketone presence~ Lipid:P	sfai_1	-0.03	1.39	-2.85	2.67	1.00	7364.55	3902.97
Ketone presence~ Lipid:P	sPREV_lipid_ap_1	-1.52	2.30	-6.95	2.18	1.00	4531.22	2557.98
Ketone presence~ Lipid:P	sds(sfai_1)	0.82	0.80	0.03	2.87	1.00	3379.22	3103.58
Ketone presence~ Lipid:P	sds(sPREV_lipid_ap_1)	1.69	1.41	0.07	5.21	1.00	3413.34	2894.38
Ketone presence~ Lipid:TNC	Intercept	-3.38	0.57	-4.54	-2.31	1.00	3696.21	4090.10
Ketone presence~ Lipid:TNC	class_focaladolescent_male	-0.54	1.73	-4.47	2.35	1.00	7192.15	2889.58
Ketone presence~ Lipid:TNC	class_focaladult_female	0.84	1.16	-1.40	3.07	1.00	8562.88	4870.11
Ketone presence~ Lipid:TNC	class_focaladult_female_with_dependent	0.81	0.59	-0.31	2.00	1.00	4046.05	3643.24
Ketone presence~ Lipid:TNC	class_focaladult_flanged_male	0.33	0.68	-0.98	1.68	1.00	3998.09	3892.18
Ketone presence~ Lipid:TNC	class_focaladult_unflanged_male	1.06	0.87	-0.65	2.80	1.00	4230.00	4423.55
Ketone presence~ Lipid:TNC	class_focalweaned_immature_female	-1.04	1.94	-5.48	1.60	1.00	4474.23	1833.38
Ketone presence~ Lipid:TNC	class_focalweaned_immature_male	0.59	1.01	-1.45	2.62	1.00	5093.46	4348.94
Ketone presence~ Lipid:TNC	sfai_1	-0.28	1.39	-3.07	2.30	1.00	5220.61	2949.99
Ketone presence~ Lipid:TNC	sPREV_lipid_tnc_1	-1.60	2.42	-7.26	2.25	1.00	3985.75	2330.75
Ketone presence~ Lipid:TNC	sds(sfai_1)	0.83	0.81	0.03	2.92	1.00	3148.32	3180.97
Ketone presence~ Lipid:TNC	sds(sPREV_lipid_tnc_1)	1.74	1.27	0.09	4.82	1.00	2476.21	1945.24
Ketone presence~ Lipid:NDF	Intercept	-3.39	0.60	-4.64	-2.30	1.00	4751.54	3927.84
Ketone presence~ Lipid:NDF	class_focaladolescent_male	-0.57	1.86	-4.65	2.49	1.00	7813.32	2342.60
Ketone presence~ Lipid:NDF	class_focaladult_female	0.85	1.21	-1.62	3.25	1.00	10200.44	3868.34
Ketone presence~ Lipid:NDF	class_focaladult_female_with_dependent	0.72	0.60	-0.43	1.93	1.00	5341.26	4389.76
Ketone presence~ Lipid:NDF	class_focaladult_flanged_male	0.34	0.69	-0.99	1.72	1.00	4966.76	4524.08
Ketone presence~ Lipid:NDF	class_focaladult_unflanged_male	1.06	0.89	-0.60	2.88	1.00	5757.90	4815.73
Ketone presence~ Lipid:NDF	class_focalweaned_immature_female	-1.01	1.75	-5.37	1.64	1.00	6373.84	2590.62
Ketone presence~ Lipid:NDF	class_focalweaned_immature_male	0.54	1.02	-1.42	2.54	1.00	6968.22	4769.27

Ketone presence~ Lipid:NDF	sfai_1	-0.20	1.38	-3.13	2.39	1.00	7789.83	3652.71
Ketone presence~ Lipid:NDF	sPREV_lipid_ndf_1	-1.31	2.43	-7.03	2.47	1.00	5823.97	2572.09
Ketone presence~ Lipid:NDF	sds(sfai_1)	0.90	0.89	0.03	3.30	1.00	3632.34	3431.90
Ketone presence~ Lipid:NDF	sds(sPREV_lipid_ndf_1)	1.86	1.45	0.08	5.30	1.00	4011.73	2816.22
Ketone presence~ TNC:P	Intercept	-3.30	0.57	-4.49	-2.24	1.00	3408.87	3257.41
Ketone presence~ TNC:P	class_focaladolescent_male	-0.55	1.76	-4.50	2.46	1.00	6733.85	2691.18
Ketone presence~ TNC:P	class_focaladult_female	0.79	1.17	-1.57	3.05	1.00	7562.13	4279.73
Ketone presence~ TNC:P	class_focaladult_female_with_dependent	0.68	0.58	-0.44	1.85	1.00	3863.52	3505.91
Ketone presence~ TNC:P	class_focaladult_flanged_male	0.34	0.67	-0.97	1.66	1.00	3515.40	3565.16
Ketone presence~ TNC:P	class_focaladult_unflanged_male	1.01	0.86	-0.64	2.72	1.00	4510.52	3639.48
Ketone presence~ TNC:P	class_focalweaned_immature_female	-0.93	1.61	-4.67	1.61	1.00	5151.62	2891.66
Ketone presence~ TNC:P	class_focalweaned_immature_male	0.58	1.01	-1.45	2.58	1.00	5079.61	3437.44
Ketone presence~ TNC:P	sfai_1	-0.13	1.40	-3.02	2.54	1.00	5084.62	3885.24
Ketone presence~ TNC:P	sPREV_tnc_ap_1	-0.54	1.44	-3.56	2.05	1.00	7190.94	4191.82
Ketone presence~ TNC:P	sds(sfai_1)	0.90	0.94	0.03	3.21	1.00	3171.36	3082.15
Ketone presence~ TNC:P	sds(sPREV_tnc_ap_1)	0.80	0.74	0.03	2.72	1.00	3982.74	2616.64
Ketone presence~ TNC:NDF	Intercept	-3.30	0.57	-4.47	-2.27	1.00	4342.80	4217.65
Ketone presence~ TNC:NDF	class_focaladolescent_male	-0.55	1.77	-4.54	2.35	1.00	6251.12	2123.67
Ketone presence~ TNC:NDF	class_focaladult_female	0.82	1.15	-1.44	3.07	1.00	10133.42	4429.71
Ketone presence~ TNC:NDF	class_focaladult_female_with_dependent	0.69	0.58	-0.44	1.86	1.00	5277.49	4442.89
Ketone presence~ TNC:NDF	class_focaladult_flanged_male	0.37	0.69	-0.97	1.75	1.00	4480.93	3958.21
Ketone presence~ TNC:NDF	class_focaladult_unflanged_male	1.05	0.86	-0.60	2.76	1.00	4762.18	4520.71
Ketone presence~ TNC:NDF	class_focalweaned_immature_female	-1.03	1.86	-5.44	1.61	1.00	4205.44	1745.95
Ketone presence~ TNC:NDF	class_focalweaned_immature_male	0.58	1.02	-1.45	2.58	1.00	6335.92	4519.66
Ketone presence~ TNC:NDF	sfai_1	-0.30	1.39	-3.22	2.36	1.00	6943.50	3732.62
Ketone presence~ TNC:NDF	sPREV_tnc_ndf_1	-0.22	1.70	-3.88	3.01	1.00	8953.00	3547.52
Ketone presence~ TNC:NDF	sds(sfai_1)	0.91	0.93	0.03	3.36	1.00	2772.63	2785.32
Ketone presence~ TNC:NDF	sds(sPREV_tnc_ndf_1)	1.01	0.91	0.03	3.34	1.00	4823.11	3325.23
Ketone presence~ P:NDF	Intercept	-3.34	0.57	-4.55	-2.30	1.00	4003.08	4136.65
Ketone presence~ P:NDF	class_focaladolescent_male	-0.56	1.79	-4.62	2.42	1.00	7226.51	2409.74
Ketone presence~ P:NDF	class_focaladult_female	0.82	1.20	-1.56	3.16	1.00	8734.43	4376.41
Ketone presence~ P:NDF	class_focaladult_female_with_dependent	0.63	0.58	-0.44	1.82	1.00	4768.00	3892.86
Ketone presence~ P:NDF	class_focaladult_flanged_male	0.39	0.68	-0.94	1.71	1.00	4306.74	4580.88
Ketone presence~ P:NDF	class_focaladult_unflanged_male	1.09	0.87	-0.57	2.88	1.00	5498.41	4715.74
Ketone presence~ P:NDF	class_focalweaned_immature_female	-0.96	1.64	-4.86	1.65	1.00	6609.76	2788.45
Ketone presence~ P:NDF	class_focalweaned_immature_male	0.63	1.00	-1.33	2.66	1.00	6083.51	4703.06
Ketone presence~ P:NDF	sfai_1	-0.18	1.35	-2.98	2.45	1.00	7249.74	4278.14
Ketone presence~ P:NDF	sPREV_ap_ndf_1	-0.22	1.97	-4.34	3.59	1.00	6820.73	2900.38
Ketone presence~ P:NDF	sds(sfai_1)	0.81	0.83	0.03	2.99	1.00	4048.12	3219.73

Ketone presence~ P:NDF	sds(sPREV_ap_ndf_1)	1.24	1.13	0.04	4.15	1.00	4697.89	3358.15
C-peptide ~ Total kcal	Intercept	7.54	0.19	7.16	7.90	1.00	1797.63	2460.98
C-peptide ~ Total kcal	class_focaladult_female	0.18	0.61	-1.02	1.41	1.00	4445.89	2586.69
C-peptide ~ Total kcal	class_focaladult_female_with_dependent	0.13	0.20	-0.26	0.53	1.00	2267.58	2622.21
C-peptide ~ Total kcal	class_focaladult_flanged_male	0.05	0.22	-0.37	0.46	1.00	2103.70	2715.96
C-peptide ~ Total kcal	class_focaladult_unflanged_male	0.06	0.47	-0.86	0.97	1.00	2872.48	2570.72
C-peptide ~ Total kcal	class_focalweaned_immature_female	0.74	0.43	-0.10	1.59	1.00	3568.66	3011.99
C-peptide ~ Total kcal	class_focalweaned_immature_male	-0.21	0.42	-1.01	0.61	1.00	2933.20	2855.06
C-peptide ~ Total kcal	sfai_1	1.65	3.27	-3.81	9.09	1.00	1823.59	1846.36
C-peptide ~ Total kcal	sprev_day_total_kcal_using_ap_low_fermentation_1	-0.72	2.69	-5.34	5.52	1.00	2139.68	2009.06
C-peptide ~ Total kcal	sucp_time_collected_1	-0.58	2.23	-4.66	4.70	1.00	2120.65	2047.79
C-peptide ~ Total kcal	sds(sfai_1)	2.35	1.44	0.60	5.88	1.00	1090.37	2228.35
C-peptide ~ Total kcal	sds(sprev_day_total_kcal_using_ap_low_fermentation_1)	1.14	0.96	0.04	3.50	1.00	1631.63	1963.08
C-peptide ~ Total kcal	sds(sucp_time_collected_1)	0.88	0.79	0.03	2.92	1.00	1713.80	1704.76
C-peptide ~ Protein	Intercept	7.53	0.19	7.17	7.91	1.00	3084.70	3078.95
C-peptide ~ Protein	class_focaladult_female	0.10	0.60	-1.11	1.26	1.00	7550.10	3002.61
C-peptide ~ Protein	class_focaladult_female_with_dependent	0.13	0.21	-0.26	0.53	1.00	3673.48	3330.12
C-peptide ~ Protein	class_focaladult_flanged_male	0.06	0.22	-0.36	0.49	1.00	2909.81	3356.07
C-peptide ~ Protein	class_focaladult_unflanged_male	0.01	0.47	-0.92	0.92	1.00	4839.41	3429.62
C-peptide ~ Protein	class_focalweaned_immature_female	0.77	0.43	-0.10	1.64	1.00	5502.92	3393.49
C-peptide ~ Protein	class_focalweaned_immature_male	-0.17	0.44	-1.03	0.69	1.00	4462.94	3224.55
C-peptide ~ Protein	sfai_1	1.87	3.27	-3.42	9.32	1.00	2107.87	2672.89
C-peptide ~ Protein	sprev_day_ap_kcal_1	-0.12	1.51	-3.53	2.82	1.00	3186.92	2222.68
C-peptide ~ Protein	sucp_time_collected_1	-0.69	2.12	-4.58	4.26	1.00	3682.34	2887.02
C-peptide ~ Protein	sds(sfai_1)	2.29	1.49	0.50	5.94	1.00	1151.47	2544.11
C-peptide ~ Protein	sds(sprev_day_ap_kcal_1)	0.57	0.58	0.02	2.10	1.00	2083.30	2192.50
C-peptide ~ Protein	sds(sucp_time_collected_1)	0.87	0.80	0.03	3.01	1.00	2314.56	2450.08
C-peptide ~ Lipid	Intercept	7.50	0.20	7.11	7.88	1.00	2295.30	2780.08
C-peptide ~ Lipid	class_focaladult_female	0.09	0.61	-1.09	1.27	1.00	5628.76	2752.47
C-peptide ~ Lipid	class_focaladult_female_with_dependent	0.19	0.21	-0.21	0.61	1.00	2058.64	2957.00
C-peptide ~ Lipid	class_focaladult_flanged_male	0.09	0.23	-0.35	0.54	1.00	2301.87	3034.43
C-peptide ~ Lipid	class_focaladult_unflanged_male	0.06	0.47	-0.84	0.96	1.00	3863.05	2992.42
C-peptide ~ Lipid	class_focalweaned_immature_female	0.81	0.42	0.00	1.63	1.00	4092.16	3028.31
C-peptide ~ Lipid	class_focalweaned_immature_male	-0.11	0.43	-0.94	0.74	1.00	3611.45	3196.50
C-peptide ~ Lipid	sfai_1	1.48	2.93	-3.39	8.11	1.00	2023.99	2316.80
C-peptide ~ Lipid	sprev_day_lipid_kcal_1	-0.33	2.10	-3.57	4.91	1.00	1729.94	1141.02
C-peptide ~ Lipid	sucp_time_collected_1	-0.60	2.18	-4.49	4.65	1.00	2315.40	1829.70
C-peptide ~ Lipid	sds(sfai_1)	2.03	1.23	0.58	5.05	1.01	1166.08	2429.80
C-peptide ~ Lipid	sds(sprev_day_lipid_kcal_1)	0.79	0.77	0.02	2.80	1.00	1560.02	2049.49

C-peptide ~ Lipid	sds(sucp_time_collected_1)	0.84	0.77	0.03	2.93	1.00	1667.26	1947.33
C-peptide ~ TNC	Intercept	7.56	0.19	7.19	7.92	1.00	2816.70	2635.33
C-peptide ~ TNC	class_focaladult_female	0.18	0.60	-1.01	1.33	1.00	6020.82	3168.10
C-peptide ~ TNC	class_focaladult_female_with_dependent	0.11	0.20	-0.29	0.49	1.00	3231.02	3159.83
C-peptide ~ TNC	class_focaladult_flanged_male	0.02	0.22	-0.41	0.45	1.00	2648.92	2876.34
C-peptide ~ TNC	class_focaladult_unflanged_male	0.05	0.47	-0.85	0.94	1.00	4370.52	3238.21
C-peptide ~ TNC	class_focalweaned_immature_female	0.74	0.42	-0.08	1.59	1.00	3909.43	2788.32
C-peptide ~ TNC	class_focalweaned_immature_male	-0.23	0.41	-1.06	0.58	1.00	3517.94	2948.55
C-peptide ~ TNC	sfai_1	1.59	3.06	-3.78	8.45	1.00	2101.86	2681.14
C-peptide ~ TNC	sprev_day_tnc_kcal_1	-1.69	3.09	-7.25	5.41	1.00	2636.23	2476.86
C-peptide ~ TNC	sucp_time_collected_1	-0.56	2.26	-4.79	4.87	1.00	2637.76	2463.24
C-peptide ~ TNC	sds(sfai_1)	2.36	1.46	0.56	5.90	1.00	1175.28	1847.75
C-peptide ~ TNC	sds(sprev_day_tnc_kcal_1)	1.25	1.06	0.05	3.94	1.00	1990.36	2001.71
C-peptide ~ TNC	sds(sucp_time_collected_1)	0.90	0.82	0.03	2.96	1.00	1822.10	2011.80
C-peptide ~ NDF	Intercept	7.53	0.19	7.15	7.91	1.00	2060.43	2641.40
C-peptide ~ NDF	class_focaladult_female	0.12	0.61	-1.05	1.31	1.00	5345.69	2664.90
C-peptide ~ NDF	class_focaladult_female_with_dependent	0.13	0.21	-0.27	0.54	1.00	2293.61	2566.51
C-peptide ~ NDF	class_focaladult_flanged_male	0.05	0.22	-0.39	0.48	1.00	2018.71	2559.26
C-peptide ~ NDF	class_focaladult_unflanged_male	0.01	0.48	-0.95	0.96	1.00	3841.79	2850.49
C-peptide ~ NDF	class_focalweaned_immature_female	0.78	0.44	-0.05	1.63	1.00	3693.77	2900.58
C-peptide ~ NDF	class_focalweaned_immature_male	-0.19	0.43	-1.02	0.68	1.00	3277.84	2964.84
C-peptide ~ NDF	sfai_1	1.92	3.31	-3.73	9.40	1.00	1840.19	2511.99
C-peptide ~ NDF	sprev_day_ndf_kcal_low_1	-0.56	1.76	-4.43	3.17	1.00	1974.54	1472.12
C-peptide ~ NDF	sucp_time_collected_1	-0.69	2.32	-5.06	4.76	1.00	2335.54	1901.37
C-peptide ~ NDF	sds(sfai_1)	2.41	1.54	0.56	6.14	1.00	1140.30	2284.71
C-peptide ~ NDF	sds(sprev_day_ndf_kcal_low_1)	0.67	0.67	0.02	2.51	1.00	1827.20	2403.12
C-peptide ~ NDF	sds(sucp_time_collected_1)	0.92	0.83	0.04	2.99	1.00	2094.73	2742.54
C-peptide ~ Npe	Intercept	7.54	0.19	7.18	7.91	1.00	2803.75	2944.67
C-peptide ~ Npe	class_focaladult_female	0.18	0.59	-0.94	1.33	1.00	6723.83	3504.96
C-peptide ~ Npe	class_focaladult_female_with_dependent	0.13	0.20	-0.28	0.52	1.00	3232.58	2905.91
C-peptide ~ Npe	class_focaladult_flanged_male	0.04	0.22	-0.39	0.48	1.00	2947.29	2833.53
C-peptide ~ Npe	class_focaladult_unflanged_male	0.07	0.47	-0.82	0.99	1.00	4190.08	3180.53
C-peptide ~ Npe	class_focalweaned_immature_female	0.75	0.42	-0.07	1.59	1.00	4269.78	3446.42
C-peptide ~ Npe	class_focalweaned_immature_male	-0.20	0.42	-1.03	0.63	1.00	4455.51	3543.28
C-peptide ~ Npe	sfai_1	1.54	3.07	-3.81	8.35	1.00	3053.53	2630.13
C-peptide ~ Npe	sprev_day_total_kcal_npe_low_fermentation_1	-0.71	2.38	-4.73	4.88	1.00	2896.39	2488.31
C-peptide ~ Npe	sucp_time_collected_1	-0.59	2.22	-4.76	4.70	1.00	2894.46	2016.87
C-peptide ~ Npe	sds(sfai_1)	2.23	1.37	0.56	5.71	1.01	1356.91	2615.85
C-peptide ~ Npe	sds(sprev_day_total_kcal_npe_low_fermentation_1)	1.03	0.85	0.04	3.11	1.00	2253.89	2389.73

C-peptide ~ Npe	sds(sucp_time_collected_1)	0.88	0.78	0.03	2.95	1.00	1784.17	1774.56
C-peptide ~ Npe:P	Intercept	7.53	0.19	7.17	7.91	1.00	1500.50	2460.33
C-peptide ~ Npe:P	class_focaladult_female	0.08	0.60	-1.12	1.24	1.00	4395.66	3118.88
C-peptide ~ Npe:P	class_focaladult_female_with_dependent	0.14	0.20	-0.26	0.53	1.00	1899.05	2841.78
C-peptide ~ Npe:P	class_focaladult_flanged_male	0.05	0.22	-0.38	0.48	1.00	1671.33	2501.39
C-peptide ~ Npe:P	class_focaladult_unflanged_male	0.01	0.46	-0.88	0.90	1.00	3069.07	3072.58
C-peptide ~ Npe:P	class_focalweaned_immature_female	0.74	0.43	-0.09	1.58	1.00	3163.35	2932.12
C-peptide ~ Npe:P	class_focalweaned_immature_male	-0.19	0.41	-0.98	0.62	1.00	2885.12	3131.25
C-peptide ~ Npe:P	sfai_1	1.68	2.95	-3.67	8.45	1.00	2032.56	1927.19
C-peptide ~ Npe:P	sprev_day_npe_ab_1	-1.67	1.73	-5.46	1.78	1.00	1971.18	1607.53
C-peptide ~ Npe:P	sucp_time_collected_1	-0.52	2.16	-4.54	4.43	1.00	2263.13	2005.47
C-peptide ~ Npe:P	sds(sfai_1)	1.96	1.18	0.55	5.07	1.00	1229.36	2038.27
C-peptide ~ Npe:P	sds(sprev_day_npe_ab_1)	0.73	0.73	0.02	2.74	1.00	1641.68	1878.75
C-peptide ~ Npe:P	sds(sucp_time_collected_1)	0.87	0.79	0.04	2.96	1.00	1847.99	2374.66
C-peptide ~ Lipid:P	Intercept	7.45	0.20	7.07	7.83	1.00	1946.78	2531.95
C-peptide ~ Lipid:P	class_focaladult_female	0.13	0.59	-1.02	1.33	1.00	4888.08	2711.99
C-peptide ~ Lipid:P	class_focaladult_female_with_dependent	0.23	0.21	-0.19	0.63	1.00	2287.10	2686.48
C-peptide ~ Lipid:P	class_focaladult_flanged_male	0.15	0.23	-0.31	0.59	1.00	2023.31	2812.15
C-peptide ~ Lipid:P	class_focaladult_unflanged_male	0.05	0.47	-0.87	0.96	1.00	3358.08	2988.91
C-peptide ~ Lipid:P	class_focalweaned_immature_female	0.87	0.42	0.04	1.68	1.00	4106.22	3115.77
C-peptide ~ Lipid:P	class_focalweaned_immature_male	-0.04	0.43	-0.87	0.80	1.00	2991.71	3268.80
C-peptide ~ Lipid:P	sfai_1	1.67	2.85	-3.24	8.01	1.00	2189.89	2898.16
C-peptide ~ Lipid:P	sPREV_lipid_ap_1	-0.01	1.77	-2.97	4.21	1.00	1738.99	2242.94
C-peptide ~ Lipid:P	sucp_time_collected_1	-0.60	2.17	-4.78	4.59	1.00	2047.94	1842.64
C-peptide ~ Lipid:P	sds(sfai_1)	1.91	1.17	0.53	4.87	1.00	1453.89	2163.78
C-peptide ~ Lipid:P	sds(sPREV_lipid_ap_1)	0.79	0.77	0.02	2.80	1.00	1589.22	1943.49
C-peptide ~ Lipid:P	sds(sucp_time_collected_1)	0.85	0.81	0.02	3.03	1.00	1825.20	2457.91
C-peptide ~ Lipid:TNC	Intercept	7.49	0.20	7.09	7.88	1.00	2318.95	2836.08
C-peptide ~ Lipid:TNC	class_focaladult_female	0.16	0.60	-0.99	1.36	1.00	4948.44	2559.33
C-peptide ~ Lipid:TNC	class_focaladult_female_with_dependent	0.18	0.21	-0.23	0.59	1.00	2673.27	2879.21
C-peptide ~ Lipid:TNC	class_focaladult_flanged_male	0.12	0.23	-0.34	0.57	1.00	2439.26	2967.58
C-peptide ~ Lipid:TNC	class_focaladult_unflanged_male	0.03	0.48	-0.92	0.92	1.00	3967.24	3244.82
C-peptide ~ Lipid:TNC	class_focalweaned_immature_female	0.85	0.42	0.03	1.66	1.00	4286.14	3473.44
C-peptide ~ Lipid:TNC	class_focalweaned_immature_male	-0.12	0.43	-0.97	0.74	1.00	3787.81	2877.76
C-peptide ~ Lipid:TNC	sfai_1	1.62	3.15	-3.68	8.77	1.00	1535.41	2997.18
C-peptide ~ Lipid:TNC	sPREV_lipid_tnc_1	-0.16	1.78	-3.71	3.85	1.00	2344.53	1938.10
C-peptide ~ Lipid:TNC	sucp_time_collected_1	-0.66	2.22	-4.89	4.77	1.00	2493.88	1991.13
C-peptide ~ Lipid:TNC	sds(sfai_1)	2.19	1.41	0.51	5.56	1.00	1158.96	2324.71
C-peptide ~ Lipid:TNC	sds(sPREV_lipid_tnc_1)	0.70	0.71	0.02	2.54	1.00	2007.00	2199.35

C-peptide ~ Lipid:TNC	sds(sucp_time_collected_1)	0.85	0.79	0.02	2.94	1.00	1485.86	1944.14
C-peptide ~ Lipid:NDF	Intercept	7.47	0.20	7.07	7.86	1.00	2341.47	2256.89
C-peptide ~ Lipid:NDF	class_focaladult_female	0.06	0.60	-1.08	1.24	1.00	5559.41	3169.24
C-peptide ~ Lipid:NDF	class_focaladult_female_with_dependent	0.22	0.21	-0.18	0.65	1.00	2673.88	2691.98
C-peptide ~ Lipid:NDF	class_focaladult_flanged_male	0.12	0.23	-0.34	0.57	1.00	2370.00	3203.09
C-peptide ~ Lipid:NDF	class_focaladult_unflanged_male	0.06	0.47	-0.84	0.97	1.00	4056.43	2985.06
C-peptide ~ Lipid:NDF	class_focalweaned_immature_female	0.82	0.42	-0.01	1.66	1.00	4558.27	2719.51
C-peptide ~ Lipid:NDF	class_focalweaned_immature_male	-0.03	0.45	-0.88	0.85	1.00	3465.88	2959.14
C-peptide ~ Lipid:NDF	sfai_1	1.66	3.15	-3.38	9.06	1.00	2382.76	2765.36
C-peptide ~ Lipid:NDF	sPREV_lipid_ndf_1	-1.30	2.86	-6.66	4.89	1.00	2537.95	2393.13
C-peptide ~ Lipid:NDF	sucp_time_collected_1	-0.69	2.26	-5.02	4.61	1.00	2286.88	1862.28
C-peptide ~ Lipid:NDF	sds(sfai_1)	2.14	1.39	0.54	5.75	1.00	1397.78	2435.56
C-peptide ~ Lipid:NDF	sds(sPREV_lipid_ndf_1)	1.10	0.94	0.04	3.66	1.00	3034.05	2413.64
C-peptide ~ Lipid:NDF	sds(sucp_time_collected_1)	0.87	0.81	0.03	3.05	1.00	2185.74	2084.51
C-peptide ~ TNC:P	Intercept	7.53	0.19	7.16	7.90	1.00	1846.76	2407.45
C-peptide ~ TNC:P	class_focaladult_female	0.13	0.61	-1.07	1.37	1.00	4541.91	2760.33
C-peptide ~ TNC:P	class_focaladult_female_with_dependent	0.14	0.20	-0.27	0.54	1.00	2151.64	2600.90
C-peptide ~ TNC:P	class_focaladult_flanged_male	0.06	0.22	-0.37	0.47	1.00	1809.69	2122.53
C-peptide ~ TNC:P	class_focaladult_unflanged_male	0.05	0.47	-0.87	0.95	1.00	3268.76	2826.89
C-peptide ~ TNC:P	class_focalweaned_immature_female	0.74	0.42	-0.06	1.58	1.00	3931.40	2757.13
C-peptide ~ TNC:P	class_focalweaned_immature_male	-0.18	0.42	-0.97	0.63	1.00	2827.34	3156.07
C-peptide ~ TNC:P	sfai_1	1.76	2.87	-3.04	8.18	1.00	1979.17	2376.71
C-peptide ~ TNC:P	sPREV_tnc_ap_1	-1.76	1.81	-5.75	2.05	1.00	1898.90	1775.11
C-peptide ~ TNC:P	sucp_time_collected_1	-0.37	2.33	-4.32	5.27	1.00	2148.32	1731.20
C-peptide ~ TNC:P	sds(sfai_1)	2.00	1.25	0.53	5.18	1.00	1234.26	2409.74
C-peptide ~ TNC:P	sds(sPREV_tnc_ap_1)	0.81	0.80	0.02	2.91	1.00	1406.49	2283.62
C-peptide ~ TNC:P	sds(sucp_time_collected_1)	0.90	0.81	0.03	2.97	1.00	1563.33	1414.74
C-peptide ~ TNC:NDF	Intercept	7.53	0.18	7.16	7.89	1.00	1888.41	2325.10
C-peptide ~ TNC:NDF	class_focaladult_female	0.13	0.59	-1.03	1.30	1.00	4697.07	2882.39
C-peptide ~ TNC:NDF	class_focaladult_female_with_dependent	0.14	0.20	-0.25	0.52	1.00	2246.66	2416.92
C-peptide ~ TNC:NDF	class_focaladult_flanged_male	0.05	0.21	-0.36	0.48	1.00	1899.35	2358.33
C-peptide ~ TNC:NDF	class_focaladult_unflanged_male	0.09	0.46	-0.83	0.99	1.00	3167.02	2841.91
C-peptide ~ TNC:NDF	class_focalweaned_immature_female	0.73	0.42	-0.08	1.57	1.00	3708.95	3061.84
C-peptide ~ TNC:NDF	class_focalweaned_immature_male	-0.17	0.41	-0.99	0.66	1.00	2912.71	3268.13
C-peptide ~ TNC:NDF	sfai_1	1.79	3.36	-3.64	9.75	1.00	1380.63	1869.53
C-peptide ~ TNC:NDF	sPREV_tnc_ndf_1	-1.64	3.30	-8.30	5.49	1.00	2243.72	2219.48
C-peptide ~ TNC:NDF	sucp_time_collected_1	-0.62	2.35	-5.13	4.95	1.00	1848.47	1894.27
C-peptide ~ TNC:NDF	sds(sfai_1)	2.36	1.65	0.49	6.43	1.00	817.43	1666.46
C-peptide ~ TNC:NDF	sds(sPREV_tnc_ndf_1)	1.56	1.28	0.06	4.72	1.00	1606.68	1763.07

C-peptide ~ TNC:NDF	sds(sucp_time_collected_1)	0.93	0.83	0.03	3.08	1.00	1832.11	1645.09
C-peptide ~ P:NDF	Intercept	7.54	0.19	7.15	7.92	1.00	2624.35	2361.92
C-peptide ~ P:NDF	class_focaladult_female	0.13	0.59	-1.02	1.30	1.00	6129.27	3150.45
C-peptide ~ P:NDF	class_focaladult_female_with_dependent	0.12	0.20	-0.27	0.51	1.00	3162.59	2930.99
C-peptide ~ P:NDF	class_focaladult_flanged_male	0.05	0.23	-0.40	0.48	1.00	2548.66	2748.14
C-peptide ~ P:NDF	class_focaladult_unflanged_male	0.00	0.46	-0.91	0.90	1.00	4172.40	2958.57
C-peptide ~ P:NDF	class_focalweaned_immature_female	0.79	0.43	-0.05	1.63	1.00	4512.13	3379.42
C-peptide ~ P:NDF	class_focalweaned_immature_male	-0.17	0.44	-1.03	0.69	1.00	3677.21	3167.09
C-peptide ~ P:NDF	sfai_1	1.84	3.32	-3.56	9.70	1.00	1899.08	2266.69
C-peptide ~ P:NDF	sPREV_ap_ndf_1	1.02	3.57	-6.13	8.48	1.00	2788.61	2431.19
C-peptide ~ P:NDF	sucp_time_collected_1	-0.73	2.21	-5.05	4.62	1.00	2542.50	2411.48
C-peptide ~ P:NDF	sds(sfai_1)	2.25	1.44	0.51	5.81	1.00	1001.57	2286.53
C-peptide ~ P:NDF	sds(sPREV_ap_ndf_1)	1.33	1.35	0.03	4.80	1.00	2101.75	1859.10
C-peptide ~ P:NDF	sds(sucp_time_collected_1)	0.88	0.80	0.03	2.97	1.00	1867.78	2272.53
d15N ~ Total kcal	Intercept	-1.28	0.45	-2.18	-0.39	1.00	2000.70	2575.35
d15N ~ Total kcal	class_focaladult_female	-1.43	1.02	-3.40	0.57	1.00	3829.34	2941.00
d15N ~ Total kcal	class_focaladult_female_with_dependent	0.26	0.47	-0.69	1.19	1.00	2357.17	3142.49
d15N ~ Total kcal	class_focaladult_flanged_male	0.11	0.52	-0.89	1.18	1.00	2126.03	2606.97
d15N ~ Total kcal	class_focaladult_unflanged_male	-0.31	0.75	-1.78	1.17	1.00	2791.62	3212.66
d15N ~ Total kcal	class_focalweaned_immature_female	-1.28	1.23	-3.71	1.16	1.00	3835.47	3145.98
d15N ~ Total kcal	class_focalweaned_immature_male	1.24	0.88	-0.49	2.94	1.00	3258.35	3082.82
d15N ~ Total kcal	sfai_1	2.64	3.68	-1.90	11.62	1.00	947.53	1757.39
d15N ~ Total kcal	sprev_day_total_kcal_using_ap_low_fermentation_1	-4.87	5.10	-16.01	4.13	1.00	2447.36	2767.51
d15N ~ Total kcal	sisotope_time_collected_1	0.78	2.05	-4.00	4.56	1.00	2800.39	2236.54
d15N ~ Total kcal	sds(sfai_1)	1.59	1.39	0.04	5.05	1.00	800.50	1793.13
d15N ~ Total kcal	sds(sprev_day_total_kcal_using_ap_low_fermentation_1)	2.81	1.48	0.71	6.39	1.00	2048.36	1922.26
d15N ~ Total kcal	sds(sisotope_time_collected_1)	0.91	0.83	0.03	3.08	1.00	1992.02	2277.17
d15N ~ Protein	Intercept	-1.27	0.48	-2.22	-0.31	1.00	2383.68	2535.65
d15N ~ Protein	class_focaladult_female	-1.55	1.09	-3.71	0.59	1.00	5195.44	3290.69
d15N ~ Protein	class_focaladult_female_with_dependent	0.24	0.50	-0.75	1.26	1.00	2689.36	2542.75
d15N ~ Protein	class_focaladult_flanged_male	0.16	0.54	-0.92	1.23	1.00	2462.00	2715.29
d15N ~ Protein	class_focaladult_unflanged_male	-0.32	0.78	-1.83	1.19	1.00	3393.24	3354.24
d15N ~ Protein	class_focalweaned_immature_female	-1.20	1.13	-3.40	1.06	1.00	4367.16	3216.17
d15N ~ Protein	class_focalweaned_immature_male	1.26	0.97	-0.69	3.17	1.00	3561.93	2887.71
d15N ~ Protein	sfai_1	2.12	3.35	-2.05	10.17	1.00	1380.45	2240.04
d15N ~ Protein	sprev_day_ap_kcal_1	-3.15	4.56	-12.79	5.51	1.00	2700.63	2889.33
d15N ~ Protein	sisotope_time_collected_1	0.49	2.24	-4.75	4.83	1.00	2875.55	2327.58
d15N ~ Protein	sds(sfai_1)	1.38	1.18	0.04	4.32	1.00	1067.95	1886.50
d15N ~ Protein	sds(sprev_day_ap_kcal_1)	2.35	1.35	0.31	5.63	1.00	2054.36	1142.60

d15N ~ Protein	sds(sisotope_time_collected_1)	0.96	0.89	0.04	3.29	1.00	2023.95	2359.58
d15N ~ Lipid	Intercept	-1.42	0.41	-2.23	-0.60	1.00	2128.95	2236.66
d15N ~ Lipid	class_focaladult_female	-1.46	1.00	-3.38	0.49	1.00	4580.86	2902.27
d15N ~ Lipid	class_focaladult_female_with_dependent	0.41	0.43	-0.43	1.26	1.00	2259.36	2531.73
d15N ~ Lipid	class_focaladult_flanged_male	0.18	0.47	-0.71	1.14	1.00	2211.06	2365.99
d15N ~ Lipid	class_focaladult_unflanged_male	-0.16	0.73	-1.66	1.23	1.00	2550.31	2888.38
d15N ~ Lipid	class_focalweaned_immature_female	-1.57	1.51	-4.56	1.52	1.00	3614.72	2983.10
d15N ~ Lipid	class_focalweaned_immature_male	1.34	0.83	-0.31	2.99	1.00	3135.61	2897.78
d15N ~ Lipid	sfai_1	3.44	3.63	-1.67	11.71	1.00	1324.98	2220.20
d15N ~ Lipid	sprev_day_lipid_kcal_1	-4.06	4.24	-13.69	2.96	1.00	2164.21	2258.34
d15N ~ Lipid	sisotope_time_collected_1	0.67	2.16	-4.16	4.67	1.00	2709.32	2349.78
d15N ~ Lipid	sds(sfai_1)	1.76	1.24	0.09	4.73	1.00	1054.24	1687.40
d15N ~ Lipid	sds(sprev_day_lipid_kcal_1)	2.45	1.56	0.27	6.43	1.00	1513.74	1520.28
d15N ~ Lipid	sds(sisotope_time_collected_1)	1.00	0.85	0.04	3.26	1.00	1790.09	1818.22
d15N ~ TNC	Intercept	-1.27	0.46	-2.16	-0.33	1.00	1440.54	1631.45
d15N ~ TNC	class_focaladult_female	-1.59	1.00	-3.55	0.42	1.00	3711.53	3017.51
d15N ~ TNC	class_focaladult_female_with_dependent	0.24	0.48	-0.68	1.19	1.00	1610.27	1961.14
d15N ~ TNC	class_focaladult_flanged_male	0.13	0.53	-0.91	1.20	1.00	1571.43	1986.39
d15N ~ TNC	class_focaladult_unflanged_male	-0.33	0.78	-1.87	1.21	1.00	1928.16	2605.27
d15N ~ TNC	class_focalweaned_immature_female	-1.14	1.15	-3.38	1.13	1.00	2963.09	2847.38
d15N ~ TNC	class_focalweaned_immature_male	1.15	0.93	-0.61	2.99	1.00	2525.79	2693.49
d15N ~ TNC	sfai_1	2.28	3.47	-2.12	10.60	1.01	1230.34	2023.90
d15N ~ TNC	sprev_day_tnc_kcal_1	-4.62	4.92	-14.57	4.67	1.00	1792.08	2552.82
d15N ~ TNC	sisotope_time_collected_1	1.21	2.04	-3.21	5.56	1.00	1969.90	1879.77
d15N ~ TNC	sds(sfai_1)	1.49	1.30	0.05	4.72	1.01	1044.76	2141.09
d15N ~ TNC	sds(sprev_day_tnc_kcal_1)	3.56	2.14	0.76	8.86	1.00	1302.83	1860.83
d15N ~ TNC	sds(sisotope_time_collected_1)	0.87	0.78	0.03	2.90	1.00	1565.53	1542.89
d15N ~ NDF	Intercept	-1.33	0.46	-2.21	-0.42	1.00	1742.61	2479.37
d15N ~ NDF	class_focaladult_female	-1.44	1.05	-3.50	0.59	1.00	5420.77	3237.71
d15N ~ NDF	class_focaladult_female_with_dependent	0.32	0.48	-0.63	1.24	1.00	2138.35	2637.16
d15N ~ NDF	class_focaladult_flanged_male	0.14	0.51	-0.86	1.18	1.00	2223.84	2788.15
d15N ~ NDF	class_focaladult_unflanged_male	-0.20	0.77	-1.73	1.31	1.00	2319.76	2696.95
d15N ~ NDF	class_focalweaned_immature_female	-1.30	1.29	-3.91	1.22	1.00	3941.14	3357.70
d15N ~ NDF	class_focalweaned_immature_male	1.35	0.92	-0.46	3.10	1.00	3341.72	3110.46
d15N ~ NDF	sfai_1	2.30	3.42	-2.00	10.66	1.00	1190.66	2823.08
d15N ~ NDF	sprev_day_ndf_kcal_low_1	-2.93	5.17	-14.39	5.85	1.00	2345.21	2817.30
d15N ~ NDF	sisotope_time_collected_1	0.55	2.38	-4.92	5.09	1.00	3175.33	2569.12
d15N ~ NDF	sds(sfai_1)	1.52	1.28	0.05	4.60	1.00	667.69	1669.36
d15N ~ NDF	sds(sprev_day_ndf_kcal_low_1)	3.34	2.19	0.15	8.41	1.00	1173.37	1693.70

d15N ~ NDF	sds(sisotope_time_collected_1)	1.09	0.87	0.06	3.34	1.00	2078.48	1940.22
d15N ~ Npe	Intercept	-1.27	0.46	-2.14	-0.31	1.00	1949.75	2330.25
d15N ~ Npe	class_focaladult_female	-1.44	1.04	-3.49	0.62	1.00	4828.44	3165.82
d15N ~ Npe	class_focaladult_female_with_dependent	0.27	0.48	-0.73	1.20	1.00	2102.94	2379.18
d15N ~ Npe	class_focaladult_flanged_male	0.09	0.52	-0.96	1.08	1.00	1913.54	2455.87
d15N ~ Npe	class_focaladult_unflanged_male	-0.31	0.77	-1.82	1.18	1.00	2874.40	2677.03
d15N ~ Npe	class_focalweaned_immature_female	-1.30	1.28	-3.86	1.17	1.00	3472.50	2854.82
d15N ~ Npe	class_focalweaned_immature_male	1.22	0.89	-0.54	2.98	1.00	3567.34	3253.12
d15N ~ Npe	sfai_1	2.66	3.61	-1.85	11.25	1.00	1275.02	2429.49
d15N ~ Npe	sprev_day_total_kcal_npe_low_fermentation_1	-4.97	5.24	-15.96	4.18	1.00	2723.44	2722.31
d15N ~ Npe	sisotope_time_collected_1	0.89	2.15	-4.15	5.09	1.00	3025.96	2499.67
d15N ~ Npe	sds(sfai_1)	1.58	1.34	0.05	4.88	1.00	1046.69	1977.51
d15N ~ Npe	sds(sprev_day_total_kcal_npe_low_fermentation_1)	2.88	1.57	0.65	6.71	1.00	2016.61	1488.34
d15N ~ Npe	sds(sisotope_time_collected_1)	0.92	0.84	0.04	3.15	1.00	2038.55	2498.23
d15N ~ Npe:P	Intercept	-1.41	0.46	-2.31	-0.48	1.00	2468.40	3528.58
d15N ~ Npe:P	class_focaladult_female	-1.61	1.12	-3.79	0.63	1.00	5702.63	4309.62
d15N ~ Npe:P	class_focaladult_female_with_dependent	0.39	0.49	-0.61	1.31	1.00	3135.39	3552.46
d15N ~ Npe:P	class_focaladult_flanged_male	0.23	0.53	-0.80	1.26	1.00	2565.32	3764.22
d15N ~ Npe:P	class_focaladult_unflanged_male	-0.16	0.78	-1.72	1.38	1.00	3666.62	3726.74
d15N ~ Npe:P	class_focalweaned_immature_female	-1.36	1.22	-3.75	1.10	1.00	5468.34	4758.92
d15N ~ Npe:P	class_focalweaned_immature_male	1.35	0.96	-0.53	3.26	1.00	3734.18	4307.52
d15N ~ Npe:P	sfai_1	2.99	3.73	-1.97	11.55	1.00	1369.97	2771.47
d15N ~ Npe:P	sprev_day_npe_ab_1	-0.70	2.99	-7.97	4.58	1.00	2998.94	2586.17
d15N ~ Npe:P	sisotope_time_collected_1	0.75	2.47	-4.88	5.63	1.00	2627.68	2979.40
d15N ~ Npe:P	sds(sfai_1)	1.61	1.23	0.08	4.66	1.00	1101.62	2162.35
d15N ~ Npe:P	sds(sprev_day_npe_ab_1)	1.16	1.08	0.03	3.95	1.00	2428.76	2630.38
d15N ~ Npe:P	sds(sisotope_time_collected_1)	1.10	0.92	0.05	3.54	1.00	2777.98	2900.83
d15N ~ Lipid:P	Intercept	-1.48	0.42	-2.32	-0.65	1.00	2298.95	3109.81
d15N ~ Lipid:P	class_focaladult_female	-1.58	1.10	-3.72	0.58	1.00	4844.90	4244.88
d15N ~ Lipid:P	class_focaladult_female_with_dependent	0.47	0.45	-0.40	1.37	1.00	2425.93	3123.67
d15N ~ Lipid:P	class_focaladult_flanged_male	0.23	0.49	-0.72	1.21	1.00	2315.53	3080.25
d15N ~ Lipid:P	class_focaladult_unflanged_male	-0.04	0.74	-1.53	1.38	1.00	2931.63	3730.73
d15N ~ Lipid:P	class_focalweaned_immature_female	-1.43	1.45	-4.22	1.47	1.00	4049.13	4201.01
d15N ~ Lipid:P	class_focalweaned_immature_male	1.40	0.89	-0.39	3.16	1.00	3901.84	4388.38
d15N ~ Lipid:P	sfai_1	3.43	3.83	-1.79	12.14	1.00	1249.66	2674.81
d15N ~ Lipid:P	sPREV_lipid_ap_1	-0.41	2.54	-6.35	4.21	1.00	2557.33	2451.28
d15N ~ Lipid:P	sisotope_time_collected_1	0.63	2.38	-4.66	5.06	1.00	2754.79	2749.03
d15N ~ Lipid:P	sds(sfai_1)	1.72	1.26	0.09	4.62	1.00	1107.53	1944.04
d15N ~ Lipid:P	sds(sPREV_lipid_ap_1)	1.04	0.88	0.05	3.31	1.00	2461.56	2779.75

d15N ~ Lipid:P	sds(sisotope_time_collected_1)	1.06	0.89	0.06	3.32	1.00	2323.92	3020.98
d15N ~ Lipid:TNC	Intercept	-1.44	0.44	-2.29	-0.54	1.00	2542.33	3194.17
d15N ~ Lipid:TNC	class_focaladult_female	-1.49	1.10	-3.62	0.66	1.00	6962.56	4498.36
d15N ~ Lipid:TNC	class_focaladult_female_with_dependent	0.43	0.47	-0.50	1.33	1.00	2763.76	3426.70
d15N ~ Lipid:TNC	class_focaladult_flanged_male	0.21	0.50	-0.75	1.20	1.00	2844.93	3157.12
d15N ~ Lipid:TNC	class_focaladult_unflanged_male	-0.14	0.76	-1.64	1.31	1.00	3442.80	3901.67
d15N ~ Lipid:TNC	class_focalweaned_immature_female	-1.11	1.29	-3.66	1.42	1.00	4348.84	4696.66
d15N ~ Lipid:TNC	class_focalweaned_immature_male	1.35	0.91	-0.50	3.14	1.00	4489.89	3792.76
d15N ~ Lipid:TNC	sfai_1	1.99	3.30	-2.22	10.01	1.00	1657.04	2700.78
d15N ~ Lipid:TNC	sPREV_lipid_tnc_1	0.16	3.17	-6.72	6.54	1.00	3754.22	2911.77
d15N ~ Lipid:TNC	sisotope_time_collected_1	0.61	2.41	-4.74	5.21	1.00	3545.34	2896.50
d15N ~ Lipid:TNC	sds(sfai_1)	1.35	1.14	0.04	4.23	1.00	1227.82	2231.29
d15N ~ Lipid:TNC	sds(sPREV_lipid_tnc_1)	1.41	1.37	0.04	4.96	1.00	2433.93	2975.48
d15N ~ Lipid:TNC	sds(sisotope_time_collected_1)	1.08	0.92	0.05	3.53	1.00	2489.75	2976.13
d15N ~ Lipid:NDF	Intercept	-1.49	0.43	-2.33	-0.63	1.00	2313.80	2653.76
d15N ~ Lipid:NDF	class_focaladult_female	-1.49	1.06	-3.58	0.64	1.00	6074.59	4603.43
d15N ~ Lipid:NDF	class_focaladult_female_with_dependent	0.47	0.46	-0.44	1.35	1.00	2479.68	3063.79
d15N ~ Lipid:NDF	class_focaladult_flanged_male	0.25	0.49	-0.70	1.23	1.00	2446.55	2723.09
d15N ~ Lipid:NDF	class_focaladult_unflanged_male	0.06	0.74	-1.43	1.50	1.00	3106.50	3441.06
d15N ~ Lipid:NDF	class_focalweaned_immature_female	-0.94	1.12	-3.19	1.27	1.00	4721.30	3745.05
d15N ~ Lipid:NDF	class_focalweaned_immature_male	1.42	0.88	-0.32	3.14	1.00	3661.02	4106.80
d15N ~ Lipid:NDF	sfai_1	2.66	3.59	-1.90	11.24	1.00	1526.45	3084.84
d15N ~ Lipid:NDF	sPREV_lipid_ndf_1	1.73	3.48	-4.13	9.94	1.00	3153.85	3359.13
d15N ~ Lipid:NDF	sisotope_time_collected_1	0.94	2.31	-4.33	5.20	1.00	2899.98	2910.84
d15N ~ Lipid:NDF	sds(sfai_1)	1.46	1.17	0.07	4.31	1.00	1305.69	2306.59
d15N ~ Lipid:NDF	sds(sPREV_lipid_ndf_1)	1.73	1.10	0.29	4.50	1.00	2599.55	2130.45
d15N ~ Lipid:NDF	sds(sisotope_time_collected_1)	1.05	0.87	0.04	3.34	1.00	2216.06	2530.89
d15N ~ TNC:P	Intercept	-1.42	0.47	-2.35	-0.50	1.00	2332.15	2937.74
d15N ~ TNC:P	class_focaladult_female	-1.60	1.11	-3.76	0.63	1.00	5264.22	4560.39
d15N ~ TNC:P	class_focaladult_female_with_dependent	0.40	0.50	-0.60	1.38	1.00	2708.19	3547.09
d15N ~ TNC:P	class_focaladult_flanged_male	0.23	0.54	-0.81	1.30	1.00	2464.11	3666.14
d15N ~ TNC:P	class_focaladult_unflanged_male	-0.15	0.79	-1.74	1.37	1.00	3392.40	4215.81
d15N ~ TNC:P	class_focalweaned_immature_female	-1.20	1.19	-3.57	1.12	1.00	4957.82	4327.89
d15N ~ TNC:P	class_focalweaned_immature_male	1.36	0.96	-0.50	3.28	1.00	3926.99	4306.98
d15N ~ TNC:P	sfai_1	2.75	3.68	-2.11	11.67	1.00	1686.50	2923.61
d15N ~ TNC:P	sPREV_tnc_ap_1	-0.76	3.03	-8.05	4.79	1.00	2897.75	2427.72
d15N ~ TNC:P	sisotope_time_collected_1	0.90	2.38	-4.16	5.83	1.00	3454.61	2793.59
d15N ~ TNC:P	sds(sfai_1)	1.55	1.20	0.07	4.50	1.00	1429.77	1677.08
d15N ~ TNC:P	sds(sPREV_tnc_ap_1)	1.28	1.27	0.04	4.67	1.00	2454.74	3280.81

d15N ~ TNC:P	sds(sisotope_time_collected_1)	1.07	0.91	0.05	3.48	1.00	2641.66	2804.07
d15N ~ TNC:NDF	Intercept	-1.37	0.45	-2.25	-0.49	1.00	3743.07	4121.73
d15N ~ TNC:NDF	class_focaladult_female	-1.70	1.10	-3.78	0.45	1.00	6988.90	5111.93
d15N ~ TNC:NDF	class_focaladult_female_with_dependent	0.34	0.48	-0.60	1.27	1.00	3933.51	3662.05
d15N ~ TNC:NDF	class_focaladult_flanged_male	0.18	0.51	-0.79	1.23	1.00	3649.57	3840.38
d15N ~ TNC:NDF	class_focaladult_unflanged_male	-0.11	0.78	-1.69	1.39	1.00	5416.53	5090.26
d15N ~ TNC:NDF	class_focalweaned_immature_female	-1.45	1.16	-3.75	0.80	1.00	6800.82	4977.77
d15N ~ TNC:NDF	class_focalweaned_immature_male	1.11	0.95	-0.73	3.00	1.00	5816.22	5293.31
d15N ~ TNC:NDF	sfai_1	2.26	3.40	-2.16	10.53	1.00	2038.15	2784.49
d15N ~ TNC:NDF	sPREV_tnc_ndf_1	-2.16	3.54	-9.38	5.58	1.00	5060.27	3495.41
d15N ~ TNC:NDF	sisotope_time_collected_1	1.09	2.33	-3.85	5.87	1.00	4762.92	3599.35
d15N ~ TNC:NDF	sds(sfai_1)	1.51	1.21	0.05	4.54	1.00	1462.68	2654.47
d15N ~ TNC:NDF	sds(sPREV_tnc_ndf_1)	1.49	1.38	0.04	5.01	1.00	2960.98	3227.84
d15N ~ TNC:NDF	sds(sisotope_time_collected_1)	1.07	0.91	0.05	3.53	1.00	2454.13	2631.49
d15N ~ P:NDF	Intercept	-1.42	0.46	-2.33	-0.50	1.00	2752.73	3409.10
d15N ~ P:NDF	class_focaladult_female	-1.63	1.11	-3.84	0.50	1.00	4955.77	4122.06
d15N ~ P:NDF	class_focaladult_female_with_dependent	0.37	0.48	-0.63	1.33	1.00	3212.03	3891.81
d15N ~ P:NDF	class_focaladult_flanged_male	0.28	0.52	-0.72	1.33	1.00	2855.87	3646.06
d15N ~ P:NDF	class_focaladult_unflanged_male	-0.13	0.80	-1.75	1.41	1.00	3606.79	3518.75
d15N ~ P:NDF	class_focalweaned_immature_female	-1.40	1.18	-3.71	0.90	1.00	5897.56	4251.41
d15N ~ P:NDF	class_focalweaned_immature_male	1.28	0.96	-0.64	3.15	1.00	4577.74	4538.84
d15N ~ P:NDF	sfai_1	2.20	3.41	-2.23	10.43	1.00	1468.36	3085.30
d15N ~ P:NDF	sPREV_ap_ndf_1	-0.76	2.22	-5.08	4.37	1.00	3741.68	3286.55
d15N ~ P:NDF	sisotope_time_collected_1	0.65	2.57	-5.02	5.45	1.00	3997.39	3813.93
d15N ~ P:NDF	sds(sfai_1)	1.43	1.20	0.05	4.36	1.00	1101.59	1811.06
d15N ~ P:NDF	sds(sPREV_ap_ndf_1)	0.88	0.82	0.03	2.98	1.00	3222.52	2842.06
d15N ~ P:NDF	sds(sisotope_time_collected_1)	1.19	0.95	0.08	3.63	1.00	3021.66	2714.97
Urea ~ Total kcal	Intercept	6.63	0.14	6.37	6.90	1.00	9462.00	9999.96
Urea ~ Total kcal	class_focaladolescent_male	0.15	0.49	-0.74	1.18	1.00	17896.32	12138.42
Urea ~ Total kcal	class_focaladult_female	0.16	0.36	-0.52	0.90	1.00	21772.53	12807.75
Urea ~ Total kcal	class_focaladult_female_with_dependent	-0.13	0.14	-0.41	0.15	1.00	10590.36	12201.02
Urea ~ Total kcal	class_focaladult_flanged_male	-0.04	0.16	-0.35	0.26	1.00	10028.98	11133.06
Urea ~ Total kcal	class_focaladult_unflanged_male	-0.19	0.26	-0.70	0.33	1.00	12768.90	12156.02
Urea ~ Total kcal	class_focalweaned_immature_female	-0.19	0.39	-0.92	0.60	1.00	13565.14	11891.95
Urea ~ Total kcal	class_focalweaned_immature_male	0.01	0.29	-0.54	0.58	1.00	13587.95	12434.31
Urea ~ Total kcal	sfai_1	-0.28	3.77	-7.36	7.29	1.00	7225.13	9758.81
Urea ~ Total kcal	sprev_day_total_kcal_using_ap_low_fermentation_1	-4.32	3.15	-9.71	2.87	1.00	11264.20	9465.34
Urea ~ Total kcal	surea_time_collected_1	-0.08	2.19	-4.68	4.41	1.00	10530.70	10681.28
Urea ~ Total kcal	sds(sfai_1)	5.39	1.70	2.82	9.40	1.00	6106.30	8019.41

Urea ~ Total kcal	sds(sprev_day_total_kcal_using_ap_low_fermentation_1)	1.27	0.98	0.06	3.71	1.00	8450.31	7801.87
Urea ~ Total kcal	sds(surea_time_collected_1)	1.31	0.99	0.09	3.83	1.00	5558.55	6843.58
Urea ~ Protein	Intercept	6.64	0.14	6.36	6.92	1.00	7061.07	9219.87
Urea ~ Protein	class_focaladolescent_male	0.15	0.50	-0.78	1.21	1.00	13669.29	11412.72
Urea ~ Protein	class_focaladult_female	0.21	0.38	-0.49	1.00	1.00	16167.15	11648.98
Urea ~ Protein	class_focaladult_female_with_dependent	-0.09	0.15	-0.38	0.19	1.00	8149.74	10898.32
Urea ~ Protein	class_focaladult_flanged_male	-0.04	0.16	-0.36	0.29	1.00	7513.23	10133.71
Urea ~ Protein	class_focaladult_unflanged_male	-0.27	0.27	-0.79	0.25	1.00	10103.74	10889.01
Urea ~ Protein	class_focalweaned_immature_female	-0.15	0.40	-0.92	0.67	1.00	12463.92	11249.25
Urea ~ Protein	class_focalweaned_immature_male	0.15	0.30	-0.43	0.75	1.00	9671.13	11428.56
Urea ~ Protein	sfai_1	-0.07	3.57	-6.84	7.23	1.00	6569.53	9004.45
Urea ~ Protein	sprev_day_ap_kcal_1	-0.35	1.75	-4.48	2.37	1.00	6964.65	7739.35
Urea ~ Protein	surea_time_collected_1	0.59	2.16	-3.88	5.30	1.00	8452.73	8835.59
Urea ~ Protein	sds(sfai_1)	5.49	1.73	2.85	9.59	1.00	4708.41	6917.60
Urea ~ Protein	sds(sprev_day_ap_kcal_1)	0.74	0.58	0.04	2.22	1.00	5435.58	6721.06
Urea ~ Protein	sds(surea_time_collected_1)	1.18	1.04	0.04	3.88	1.00	4823.32	6413.08
Urea ~ Lipid	Intercept	6.65	0.13	6.39	6.91	1.00	6820.62	9464.79
Urea ~ Lipid	class_focaladolescent_male	0.16	0.48	-0.70	1.16	1.00	14614.74	11169.88
Urea ~ Lipid	class_focaladult_female	0.20	0.36	-0.47	0.96	1.00	16113.73	11443.19
Urea ~ Lipid	class_focaladult_female_with_dependent	-0.10	0.14	-0.38	0.17	1.00	7626.68	10408.51
Urea ~ Lipid	class_focaladult_flanged_male	-0.06	0.15	-0.37	0.23	1.00	7002.92	9643.52
Urea ~ Lipid	class_focaladult_unflanged_male	-0.16	0.26	-0.66	0.35	1.00	9548.77	11122.92
Urea ~ Lipid	class_focalweaned_immature_female	-0.17	0.39	-0.91	0.62	1.00	11700.99	11658.88
Urea ~ Lipid	class_focalweaned_immature_male	0.03	0.27	-0.50	0.58	1.00	9600.21	10483.65
Urea ~ Lipid	sfai_1	-1.05	3.21	-7.08	5.41	1.00	6740.98	8418.36
Urea ~ Lipid	sprev_day_lipid_kcal_1	-4.68	2.65	-9.98	0.59	1.00	7729.23	9206.17
Urea ~ Lipid	surea_time_collected_1	-0.03	2.05	-4.49	4.17	1.00	7776.98	9011.54
Urea ~ Lipid	sds(sfai_1)	5.03	1.56	2.68	8.83	1.00	4441.48	6866.64
Urea ~ Lipid	sds(sprev_day_lipid_kcal_1)	1.98	0.98	0.69	4.42	1.00	6225.07	9110.29
Urea ~ Lipid	sds(surea_time_collected_1)	1.17	0.95	0.05	3.54	1.00	4414.19	5195.60
Urea ~ TNC	Intercept	6.63	0.14	6.36	6.91	1.00	8983.13	10555.75
Urea ~ TNC	class_focaladolescent_male	0.15	0.47	-0.72	1.16	1.00	16423.28	11658.73
Urea ~ TNC	class_focaladult_female	0.13	0.36	-0.54	0.88	1.00	18533.22	12079.55
Urea ~ TNC	class_focaladult_female_with_dependent	-0.14	0.14	-0.43	0.14	1.00	10308.92	12105.49
Urea ~ TNC	class_focaladult_flanged_male	-0.05	0.16	-0.36	0.26	1.00	9333.78	11211.02
Urea ~ TNC	class_focaladult_unflanged_male	-0.19	0.26	-0.71	0.32	1.00	12038.41	12565.01
Urea ~ TNC	class_focalweaned_immature_female	-0.21	0.39	-0.95	0.56	1.00	15533.81	13315.88
Urea ~ TNC	class_focalweaned_immature_male	-0.02	0.29	-0.58	0.55	1.00	11570.66	11935.59
Urea ~ TNC	sfai_1	-0.47	3.83	-7.71	7.35	1.00	7433.80	10127.95

Urea ~ TNC	sprev_day_tnc_kcal_1	-7.42	4.18	-14.89	1.65	1.00	11328.94	10836.37
Urea ~ TNC	surea_time_collected_1	-0.20	2.25	-5.02	4.37	1.00	10389.84	10168.13
Urea ~ TNC	sds(sfai_1)	5.71	1.77	3.07	9.85	1.00	5170.78	8445.49
Urea ~ TNC	sds(sprev_day_tnc_kcal_1)	1.78	1.32	0.10	5.04	1.00	7218.82	6753.71
Urea ~ TNC	sds(surea_time_collected_1)	1.46	1.05	0.11	4.03	1.00	5747.34	6498.79
Urea ~ NDF	Intercept	6.64	0.14	6.36	6.92	1.00	7198.88	10130.86
Urea ~ NDF	class_focaladolescent_male	0.14	0.51	-0.78	1.22	1.00	13922.25	12191.40
Urea ~ NDF	class_focaladult_female	0.17	0.37	-0.52	0.94	1.00	18330.56	12025.39
Urea ~ NDF	class_focaladult_female_with_dependent	-0.08	0.15	-0.37	0.21	1.00	8375.39	11252.83
Urea ~ NDF	class_focaladult_flanged_male	-0.01	0.16	-0.34	0.30	1.00	7656.11	10941.40
Urea ~ NDF	class_focaladult_unflanged_male	-0.22	0.27	-0.76	0.30	1.00	9812.65	10755.30
Urea ~ NDF	class_focalweaned_immature_female	-0.13	0.41	-0.93	0.70	1.00	13972.51	12790.14
Urea ~ NDF	class_focalweaned_immature_male	0.12	0.29	-0.44	0.70	1.00	10003.04	11415.14
Urea ~ NDF	sfai_1	-2.41	3.23	-8.43	4.17	1.00	6781.85	9150.48
Urea ~ NDF	sprev_day_ndf_kcal_low_1	-1.93	1.58	-5.65	0.89	1.00	8308.23	7066.64
Urea ~ NDF	surea_time_collected_1	0.31	2.08	-4.02	4.84	1.00	9087.23	8914.71
Urea ~ NDF	sds(sfai_1)	4.62	1.52	2.30	8.21	1.00	4920.80	6718.99
Urea ~ NDF	sds(sprev_day_ndf_kcal_low_1)	0.61	0.61	0.02	2.26	1.00	6959.37	7916.49
Urea ~ NDF	sds(surea_time_collected_1)	1.24	1.05	0.05	3.87	1.00	4612.68	7289.01
Urea ~ Npe	Intercept	6.63	0.14	6.36	6.90	1.00	8503.21	9744.06
Urea ~ Npe	class_focaladolescent_male	0.15	0.49	-0.74	1.18	1.00	18129.80	12345.43
Urea ~ Npe	class_focaladult_female	0.14	0.36	-0.52	0.88	1.00	17704.96	12747.10
Urea ~ Npe	class_focaladult_female_with_dependent	-0.13	0.14	-0.41	0.15	1.00	9658.48	11341.12
Urea ~ Npe	class_focaladult_flanged_male	-0.05	0.16	-0.36	0.25	1.00	8767.33	11588.86
Urea ~ Npe	class_focaladult_unflanged_male	-0.18	0.26	-0.69	0.32	1.00	11798.57	12047.66
Urea ~ Npe	class_focalweaned_immature_female	-0.18	0.38	-0.92	0.59	1.00	15102.37	12420.16
Urea ~ Npe	class_focalweaned_immature_male	0.00	0.28	-0.54	0.56	1.00	13173.66	12776.11
Urea ~ Npe	sfai_1	-0.37	3.71	-7.34	7.17	1.00	8094.60	9904.21
Urea ~ Npe	sprev_day_total_kcal_npe_low_fermentation_1	-4.84	3.13	-10.23	2.12	1.00	10145.10	9908.78
Urea ~ Npe	surea_time_collected_1	-0.14	2.16	-4.67	4.30	1.00	11176.25	9607.23
Urea ~ Npe	sds(sfai_1)	5.50	1.71	2.95	9.53	1.00	5003.01	8330.66
Urea ~ Npe	sds(sprev_day_total_kcal_npe_low_fermentation_1)	1.32	1.06	0.06	3.96	1.00	7877.98	7722.61
Urea ~ Npe	sds(surea_time_collected_1)	1.34	0.99	0.08	3.84	1.00	4335.90	4786.06
Urea ~ Npe:P	Intercept	6.61	0.14	6.35	6.89	1.00	7559.39	9509.54
Urea ~ Npe:P	class_focaladolescent_male	0.21	0.46	-0.64	1.16	1.00	15225.08	12696.31
Urea ~ Npe:P	class_focaladult_female	0.09	0.32	-0.52	0.75	1.00	19306.91	12081.23
Urea ~ Npe:P	class_focaladult_female_with_dependent	-0.11	0.14	-0.38	0.16	1.00	9489.00	10937.26
Urea ~ Npe:P	class_focaladult_flanged_male	-0.10	0.16	-0.41	0.20	1.00	8471.16	10496.88
Urea ~ Npe:P	class_focaladult_unflanged_male	-0.24	0.25	-0.74	0.25	1.00	9773.62	11033.65

Urea ~ Npe:P	class_focalweaned_immature_female	-0.14	0.40	-0.90	0.66	1.00	14214.80	12297.26
Urea ~ Npe:P	class_focalweaned_immature_male	-0.03	0.27	-0.54	0.50	1.00	10901.00	11862.95
Urea ~ Npe:P	sfai_1	-2.65	3.11	-8.65	3.56	1.00	9108.63	9796.27
Urea ~ Npe:P	sprev_day_npe_ab_1	-4.71	1.60	-8.39	-1.92	1.00	10203.16	8498.82
Urea ~ Npe:P	surea_time_collected_1	-0.19	2.01	-4.54	3.79	1.00	10752.71	9897.27
Urea ~ Npe:P	sds(sfai_1)	5.31	1.59	2.91	9.06	1.00	5998.07	9236.35
Urea ~ Npe:P	sds(sprev_day_npe_ab_1)	0.69	0.57	0.03	2.17	1.00	7055.94	7855.83
Urea ~ Npe:P	sds(surea_time_collected_1)	1.14	0.86	0.07	3.27	1.00	5895.93	6415.48
Urea ~ Lipid:P	Intercept	6.65	0.13	6.40	6.91	1.00	8031.21	11010.29
Urea ~ Lipid:P	class_focaladolescent_male	0.12	0.47	-0.73	1.10	1.00	17016.40	12135.83
Urea ~ Lipid:P	class_focaladult_female	0.16	0.35	-0.49	0.88	1.00	20744.91	11808.21
Urea ~ Lipid:P	class_focaladult_female_with_dependent	-0.11	0.14	-0.38	0.15	1.00	8756.73	11929.98
Urea ~ Lipid:P	class_focaladult_flanged_male	-0.12	0.15	-0.42	0.18	1.00	8412.15	10791.74
Urea ~ Lipid:P	class_focaladult_unflanged_male	-0.22	0.25	-0.71	0.27	1.00	11505.36	12179.19
Urea ~ Lipid:P	class_focalweaned_immature_female	-0.19	0.39	-0.94	0.61	1.00	14742.45	12280.28
Urea ~ Lipid:P	class_focalweaned_immature_male	-0.05	0.27	-0.58	0.48	1.00	11581.11	12509.58
Urea ~ Lipid:P	sfai_1	1.28	3.31	-5.02	7.84	1.00	8103.08	9426.58
Urea ~ Lipid:P	sPREV_lipid_ap_1	-3.96	2.24	-8.61	0.29	1.00	10183.28	10341.35
Urea ~ Lipid:P	surea_time_collected_1	-0.16	1.98	-4.48	3.72	1.00	9662.68	9460.14
Urea ~ Lipid:P	sds(sfai_1)	5.76	1.70	3.23	9.80	1.00	5504.66	7808.56
Urea ~ Lipid:P	sds(sPREV_lipid_ap_1)	1.38	0.70	0.48	3.18	1.00	8812.49	11092.81
Urea ~ Lipid:P	sds(surea_time_collected_1)	1.14	0.93	0.05	3.47	1.00	5508.70	7110.14
Urea ~ Lipid:TNC	Intercept	6.65	0.14	6.38	6.93	1.00	8981.06	10752.73
Urea ~ Lipid:TNC	class_focaladolescent_male	0.11	0.51	-0.82	1.18	1.00	16708.20	11445.25
Urea ~ Lipid:TNC	class_focaladult_female	0.20	0.38	-0.50	0.97	1.00	21319.37	12133.98
Urea ~ Lipid:TNC	class_focaladult_female_with_dependent	-0.07	0.15	-0.36	0.21	1.00	9665.90	11880.20
Urea ~ Lipid:TNC	class_focaladult_flanged_male	-0.04	0.16	-0.35	0.28	1.00	8460.09	11244.88
Urea ~ Lipid:TNC	class_focaladult_unflanged_male	-0.22	0.27	-0.75	0.31	1.00	12381.78	12133.08
Urea ~ Lipid:TNC	class_focalweaned_immature_female	-0.18	0.41	-0.96	0.65	1.00	15536.07	13078.79
Urea ~ Lipid:TNC	class_focalweaned_immature_male	0.13	0.29	-0.44	0.71	1.00	11771.92	12335.42
Urea ~ Lipid:TNC	sfai_1	-1.38	3.31	-7.53	5.33	1.00	8781.73	9606.08
Urea ~ Lipid:TNC	sPREV_lipid_tnc_1	-2.87	2.63	-8.69	1.84	1.00	8509.19	10351.61
Urea ~ Lipid:TNC	surea_time_collected_1	0.50	2.15	-4.01	5.07	1.00	9387.65	8556.58
Urea ~ Lipid:TNC	sds(sfai_1)	5.00	1.65	2.51	8.86	1.00	5002.52	7477.65
Urea ~ Lipid:TNC	sds(sPREV_lipid_tnc_1)	1.53	1.04	0.13	4.03	1.00	4124.51	5047.70
Urea ~ Lipid:TNC	sds(surea_time_collected_1)	1.20	1.04	0.04	3.81	1.00	5120.96	6789.32
Urea ~ Lipid:NDF	Intercept	6.65	0.14	6.38	6.92	1.00	7463.37	9782.47
Urea ~ Lipid:NDF	class_focaladolescent_male	0.13	0.48	-0.76	1.15	1.00	17571.32	11869.65
Urea ~ Lipid:NDF	class_focaladult_female	0.20	0.36	-0.47	0.95	1.00	18165.98	11776.07

Urea ~ Lipid:NDF	class_focaladult_female_with_dependent	-0.08	0.14	-0.36	0.20	1.00	7921.21	10016.09
Urea ~ Lipid:NDF	class_focaladult_flanged_male	-0.07	0.16	-0.38	0.24	1.00	7831.06	10302.29
Urea ~ Lipid:NDF	class_focaladult_unflanged_male	-0.21	0.26	-0.71	0.29	1.00	9952.37	11230.16
Urea ~ Lipid:NDF	class_focalweaned_immature_female	-0.20	0.38	-0.92	0.60	1.00	12927.12	11874.16
Urea ~ Lipid:NDF	class_focalweaned_immature_male	0.03	0.28	-0.52	0.59	1.00	10308.54	12124.40
Urea ~ Lipid:NDF	sfai_1	-0.60	3.23	-6.70	5.96	1.00	7440.11	8929.64
Urea ~ Lipid:NDF	sPREV_lipid_ndf_1	-3.42	3.65	-10.77	4.01	1.00	9293.38	9066.20
Urea ~ Lipid:NDF	surea_time_collected_1	0.18	2.10	-4.52	4.41	1.00	8529.11	8332.59
Urea ~ Lipid:NDF	sds(sfai_1)	5.55	1.71	2.98	9.61	1.00	5328.54	6969.96
Urea ~ Lipid:NDF	sds(sPREV_lipid_ndf_1)	2.28	1.30	0.49	5.57	1.00	7398.88	5851.08
Urea ~ Lipid:NDF	sds(surea_time_collected_1)	1.20	1.02	0.04	3.77	1.00	4606.48	6205.80
Urea ~ TNC:P	Intercept	6.66	0.14	6.38	6.94	1.00	8975.07	10617.81
Urea ~ TNC:P	class_focaladolescent_male	0.17	0.46	-0.69	1.13	1.00	17657.70	13003.78
Urea ~ TNC:P	class_focaladult_female	0.08	0.34	-0.55	0.78	1.00	23680.62	12030.69
Urea ~ TNC:P	class_focaladult_female_with_dependent	-0.15	0.14	-0.43	0.13	1.00	9960.13	12274.73
Urea ~ TNC:P	class_focaladult_flanged_male	-0.13	0.16	-0.45	0.18	1.00	9271.65	11310.99
Urea ~ TNC:P	class_focaladult_unflanged_male	-0.27	0.26	-0.77	0.24	1.00	12734.52	12768.38
Urea ~ TNC:P	class_focalweaned_immature_female	-0.23	0.38	-0.96	0.53	1.00	14123.61	12611.11
Urea ~ TNC:P	class_focalweaned_immature_male	-0.03	0.27	-0.56	0.51	1.00	12358.83	12350.88
Urea ~ TNC:P	sfai_1	-3.01	3.18	-9.03	3.33	1.00	8983.04	11715.49
Urea ~ TNC:P	sPREV_tnc_ap_1	-5.55	2.23	-10.47	-1.44	1.00	11654.06	11312.07
Urea ~ TNC:P	surea_time_collected_1	-0.21	2.16	-4.98	4.05	1.00	11581.16	10360.55
Urea ~ TNC:P	sds(sfai_1)	5.43	1.63	2.94	9.25	1.00	6376.34	8911.44
Urea ~ TNC:P	sds(sPREV_tnc_ap_1)	1.29	0.79	0.15	3.19	1.00	7301.51	6406.31
Urea ~ TNC:P	sds(surea_time_collected_1)	1.22	0.93	0.06	3.56	1.00	5866.04	7992.37
Urea ~ TNC:NDF	Intercept	6.70	0.14	6.43	6.97	1.00	6321.08	8805.64
Urea ~ TNC:NDF	class_focaladolescent_male	0.12	0.49	-0.77	1.13	1.00	13757.17	11321.73
Urea ~ TNC:NDF	class_focaladult_female	0.17	0.37	-0.51	0.94	1.00	17236.58	11567.31
Urea ~ TNC:NDF	class_focaladult_female_with_dependent	-0.12	0.14	-0.41	0.15	1.00	7684.03	10418.73
Urea ~ TNC:NDF	class_focaladult_flanged_male	-0.10	0.16	-0.42	0.21	1.00	6835.72	9444.70
Urea ~ TNC:NDF	class_focaladult_unflanged_male	-0.28	0.26	-0.80	0.24	1.00	9395.87	11491.13
Urea ~ TNC:NDF	class_focalweaned_immature_female	-0.33	0.41	-1.12	0.50	1.00	10851.77	10854.79
Urea ~ TNC:NDF	class_focalweaned_immature_male	0.03	0.28	-0.53	0.59	1.00	9551.08	10882.90
Urea ~ TNC:NDF	sfai_1	-0.24	3.68	-7.17	7.34	1.00	5391.59	8022.01
Urea ~ TNC:NDF	sPREV_tnc_ndf_1	-5.98	4.41	-14.68	2.72	1.00	10543.22	11343.53
Urea ~ TNC:NDF	surea_time_collected_1	0.05	2.29	-5.10	4.59	1.00	8389.43	8019.40
Urea ~ TNC:NDF	sds(sfai_1)	6.00	1.83	3.25	10.30	1.00	5247.55	7459.91
Urea ~ TNC:NDF	sds(sPREV_tnc_ndf_1)	3.94	1.65	1.53	7.95	1.00	9771.68	9419.17
Urea ~ TNC:NDF	sds(surea_time_collected_1)	1.35	1.10	0.06	4.10	1.00	4781.21	7385.21

Urea ~ P:NDF	Intercept	6.64	0.14	6.36	6.92	1.00	9396.19	11021.86
Urea ~ P:NDF	class_focaladolescent_male	0.15	0.50	-0.76	1.20	1.00	17698.90	10752.91
Urea ~ P:NDF	class_focaladult_female	0.16	0.37	-0.52	0.93	1.00	21723.73	11999.96
Urea ~ P:NDF	class_focaladult_female_with_dependent	-0.06	0.15	-0.34	0.22	1.00	10680.65	11264.41
Urea ~ P:NDF	class_focaladult_flanged_male	-0.04	0.17	-0.37	0.28	1.00	9489.85	11516.78
Urea ~ P:NDF	class_focaladult_unflanged_male	-0.25	0.27	-0.78	0.30	1.00	12584.21	12797.03
Urea ~ P:NDF	class_focalweaned_immature_female	-0.07	0.42	-0.88	0.78	1.00	16098.69	12703.60
Urea ~ P:NDF	class_focalweaned_immature_male	0.17	0.30	-0.41	0.75	1.00	12266.97	12687.70
Urea ~ P:NDF	sfai_1	-1.48	3.32	-7.67	5.36	1.00	8788.49	9674.04
Urea ~ P:NDF	sPREV_ap_ndf_1	5.04	4.55	-3.66	14.70	1.00	13611.17	10561.44
Urea ~ P:NDF	surea_time_collected_1	0.50	2.02	-3.72	4.83	1.00	10845.56	9546.71
Urea ~ P:NDF	sds(sfai_1)	4.68	1.51	2.41	8.26	1.00	5468.91	9150.03
Urea ~ P:NDF	sds(sPREV_ap_ndf_1)	2.70	1.63	0.37	6.75	1.00	8411.45	5978.83
Urea ~ P:NDF	sds(surea_time_collected_1)	1.09	0.94	0.04	3.52	1.00	5970.57	7639.82

table S6. Full results of Bayesian models (n = 52 models) based on weak priors testing the probability that the prior day daily macronutrient intake (Kcal) and ratios vary with the next day urinary metabolites. All models included fruit availability index (FAI), sex class as a fixed effect, and orangutan ID as a random effect. TNC = total non-structural carbohydrates; P = available protein; NDF = neutral detergent fiber; NPe = non-protein energy. (Ketone presence n=782; C-peptide n=422; urea n=632; δN^{15} n=131). A condensed version of this table is provided in Table 2. Total kcal = total daily caloric intake (kcal); NPe = non-protein energy; TNC = total non-structural carbohydrates (kcal); NDF = neutral detergent fiber (kcal); P = available protein (kcal).

Plant Part	# of Species	% Kcal Protein	%Kcal Lipids	% Kcal NDF	% Kcal TNC	% Kcal Total Carbohydrates
**Aril**	2	6.59	28.91	1.01	63.50	64.50
**Inner cambium**	6	9.12	10.75	16.87	63.26	80.13
**Flower**	7	18.12	12.19	11.63	58.05	69.68
**Honey**	1	2.10	0.00	0.43	97.48	97.90
**Fruit husk**	32	10.53	17.66	15.89	55.92	71.81
**Insects**	3	70.96	22.16	1.78	5.11	6.89
**Mature leaves**	9	21.43	11.97	13.11	53.49	66.60
**Pith**	5	28.93	4.33	22.97	43.77	66.74
**Fruit pulp**	62	10.52	15.69	10.68	63.10	73.78
**Pulp & husk**	6	11.47	6.69	10.73	71.10	81.83
**Pulp & seed**	9	15.23	17.67	11.80	55.30	67.10
**Seed**	54	10.86	26.33	10.92	51.90	62.81
**Whole fruit**	14	14.95	16.64	9.95	58.47	68.41
**Young leaves**	15	32.07	3.93	13.22	50.77	63.99

table S7. The percentage of kilocalories from macronutrients in the foods consumed by wild orangutans in the Tuanan orangutan research area. The digestibility coefficient for neutral detergent fiber (NDF) is the low digestibility coefficient and protein is the available protein as described in detail in (*18*). TNC = Total non-structural carbohydrates; NDF = Neutral detergent fiber; Total carbohydrates = NDF + TNC.

Age-sex class	# of individuals	# follows	Years of Collection
Adult female without dependent	5	71	2003-2018
Adult female with dependent	21	2585	2003-2018
Adult flanged male	48	1090	2003-2018
Adult unflanged male	25	388	2003-2018
Adolescent female	13	536	2003-2018
Adolescent male	7	61	2003-2018
Weaned immature female	6	207	2003-2018
Weaned immature male	9	195	2003-2018

table S8. The number of individuals and the number of full-day focal animal follows in each age-sex class included in the nutritional intake dataset. Adolescents are defined as individuals who travel independently of their mothers and are nulliparous (females) or have not been observed to mate with females (males). Weaned immature individuals still travel with their mothers but have a younger sibling present, sleep in their own nest, and thus are presumed weaned.

Analysis	Age-sex class	# of urine samples from high fruit period	# of urine sampl es from low fruit period	Total # urine samples used in biomarker FAI analyses	# of urine samples from high fruit periods with previous day nutritional data	# of urine samples from low fruit periods with previous day nutritional data	Total # urine samples with matched previous day nutritional intakes
Ketones	Adult female without dependent	4	5	9	3	2	5
	Adult female with dependent	143	423	566	111	316	427
	Adult flanged male	85	274	359	47	174	221
	Adult unflanged male	13	23	36	9	14	23
	Adolescent female	25	78	103	14	64	78
	Adolescent male	1	8	9	0	3	3
	Weaned immature female	5	8	13	3	7	10
	Weaned immature male	5	15	20	5	10	15
	**TOTAL**	**281**	**834**	**1115**	**192**	**590**	**782**
UCP	Adult female without dependent	5	1	6	3	0	3
	Adult female with dependent	98	164	262	73	120	193
	Adult flanged male	67	154	221	41	114	155
	Adult unflanged male	5	6	11	3	3	6
	Adolescent female	14	45	59	9	41	50
	Adolescent male	1	2	3	0	0	0
	Weaned immature female	4	5	9	3	4	7
	Weaned immature male	5	4	9	**5**	3	8
	**TOTAL**	**199**	**381**	**580**	**137**	**285**	**422**
Urea	Adult female without dependent	5	5	10	3	2	5
	Adult female with dependent	112	311	423	81	230	311
	Adult flanged male	83	264	347	46	168	214
	Adult unflanged male	8	20	28	4	13	17
	Adolescent female	19	60	79	12	50	62
	Adolescent male	1	8	9	0	3	3
	Weaned immature female	2	7	9	0	6	6
	Weaned immature male	5	14	19	5	9	14

	**TOTAL**	**235**	**689**	**924**	**151**	**481**	**632**
dN15	Adult female without dependent	0	2	2	0	1	1
	Adult female with dependent	34	65	99	22	49	71
	Adult flanged male	14	53	67	5	33	38
	Adult unflanged male	5	1	6	5	0	5
	Adolescent female	10	7	17	6	7	13
	Adolescent male	0	0	0	0	0	0
	Weaned immature female	0	1	1	0	1	1
	Weaned immature male	1	2	3	1	1	2
	**TOTAL**	**64**	**131**	**195**	**39**	**92**	**131**

table S9. Number of urine samples from unique genetically identified individuals collected from full day follows. Data are broken down by fruiting period and if samples were paired with previous day nutritional intakes. Ketone samples were collected from 2003-2018; UCP from 2009-2016; Urea from 2009-2018; and dN15 2011-2015. Adolescents are defined as individuals who travel independently of their mothers and are nulliparous (females) or have not been observed to mate with females (males). Weaned immature individuals still travel with their mothers but have a younger sibling present, sleep in their own nest, and thus are presumed weaned

Other Supplementary Materials for this manuscript include the following:

Data S1(Vogeletal_MetabolicFlexibility_TuananOrangutans_FINAL.csv) can be found on Dryad https://doi.org/10.5061/dryad.c59zw3rjx).

REFERENCES AND NOTES

1. P. S. Barboza, K. L. Parker, I. D. Hume, *Intergrative Wildlife Nutrition* (Springer-Verlag, 2009).

2. J. Freese, R. J. Klement, B. Ruiz-Núñez, S. Schwarz, H. Lötzerich, The sedentary (r)evolution: Have we lost our metabolic flexibility? *F1000Res* **6**, 1787 (2017).

3. R. L. Smith, M. R. Soeters, R. C. I. Wüst, R. H. Houtkooper, Metabolic flexibility as an adaptation to energy resources and requirements in health and disease. *Endocr. Rev.* **39**, 489–517 (2018).

4. K. A. Carscadden, N. C. Emery, C. A. Arnillas, M. W. Cadotte, M. E. Afkhami, D. Gravel, S. W. Livingstone, J. J. Wiens, Niche breadth: Causes and consequences for ecology, evolution, and conservation. *Q. Rev. Biol.* **95**, 179–214 (2020).

5. S. J. Simpson, D. Raubenheimer, *The Nature of Nutrition: A Unifying Framework from Animal Adaptation to Human Obesity* (Princeton Univ. Press, 2012), pp. 229.

6. S. C. Cotter, S. J. Simpson, D. Raubenheimer, K. Wilson, Macronutrient balance mediates trade-offs between immune function and life history traits. *Funct. Ecol.* **25**, 186–198 (2011).

7. X. Huang, D. P. Hancock, A. K. Gosby, A. C. McMahon, S. M. C. Solon, D. G. Le Couteur, A. D. Conigrave, D. Raubenheimer, S. J. Simpson, Effects of dietary protein to carbohydrate balance on energy intake, fat storage, and heat production in mice. *Obesity* **21**, 85–92 (2013).

8. D. Raubenheimer, S. J. Simpson, Protein appetite as an integrator in the obesity system: The protein leverage hypothesis. *Philos. Trans. R. Soc. Lond. B Biol. Sci.* **378**, 20220212 (2023).

9. S. M. Solon-Biet, S. J. Mitchell, R. de Cabo, D. Raubenheimer, D. G. Le Couteur, S. J. Simpson, Macronutrients and caloric intake in health and longevity. *J. Endocrinol.* **226**, R17–R28 (2015).

10. S. Solon, A. McMahon, J. W. O. Ballard, D. Raubenheimer, G. J. Cooney, D. G. Le Couteur, S. J. Simpson, The effects of balance of macronutrients on ageing and glucose tolerance. *Australas. J. Ageing* **31**, 33–33 (2012).

11. M. L. Blumfield, C. Nowson, A. J. Hure, R. Smith, S. J. Simpson, D. Raubenheimer, L. MacDonald-Wicks, C. E. Collins, Lower protein-to-carbohydrate ratio in maternal diet is associated with higher childhood systolic blood pressure up to age four years. *Nutrients* **7**, 3078–3093 (2015).

12. S. J. Simpson, D. Raubenheimer, A multilevel analysis of feeding-behavior - the geometry of nutritional decisions. *Philos. Trans. R. Soc. Lond. B Biol. Sci.* **342**, 381–402 (1993).

13. S. J. Simpson, R. M. Sibly, K. P. Lee, S. T. Behmer, D. Raubenheimer, Optimal foraging when regulating intake of multiple nutrients. *Anim. Behav.* **68**, 1299–1311 (2004).

14. D. Raubenheimer, S. J. Simpson, D. Mayntz, Nutrition, ecology and nutritional ecology: Toward an integrated framework. *Funct. Ecol.* **23**, 4–16 (2009).

15. A. I. Houston, A. D. Higginson, J. M. McNamara, Optimal foraging for multiple nutrients in an unpredictable environment. *Ecol. Lett.* **14**, 1101–1107 (2011).

16. D. Raubenheimer, R. Hou, Y. Dong, C. Ren, Z. Cui, Towards an integrated understanding of dietary phenotypes. *Philos. Trans. R. Soc. Lond. B Biol. Sci.* **378**, 20220545 (2023).

17. A. M. Felton, A. Felton, D. B. Lindenmayer, W. J. Foley, Nutritional goals of wild primates. *Funct. Ecol.* **23**, 70–78 (2009).

18. J. M. Rothman, D. Raubenheimer, C. A. Chapman, Nutritional geometry: Gorillas prioritize non-protein energy while consuming surplus protein. *Biol. Lett.* **7**, 847–849 (2011).

19. A. M. Felton, A. Felton, D. Raubenheimer, S. J. Simpson, W. J. Foley, J. T. Wood, I. R. Wallis, D. B. Lindenmayer, Protein content of diets dictates the daily energy intake of a free-ranging primate. *Behav. Ecol.* **20**, 685–690 (2009).

20. M. Q. Takahashi, J. M. Rothman, D. Raubenheimer, M. Cords, Daily protein prioritization and long-term nutrient balancing in a dietary generalist, the blue monkey. *Behav. Ecol.* **32**, 223–235 (2021).

21. N. Beeby, J. M. Rothman, A. L. Baden, Nutrient balancing in a fruit-specialist primate, the black-and-white ruffed lemur (*Varecia variegata*). *Am. J. Primatol.* **85**, e23484 (2023).

22. M. Uwimbabazi, D. Raubenheimer, M. Tweheyo, G. I. Basuta, N. L. Conklin-Brittain, R. W. Wrangham, J. M. Rothman, Nutritional geometry of female chimpanzees (*Pan troglodytes*). *Am. J. Primatol.* **83**, e23269 (2021).

23. Z.-W. Cui, Z.-L. Wang, Q. Shao, D. Raubenheimer, J.-Q. Lu, Macronutrient signature of dietary generalism in an ecologically diverse primate in the wild. *Behav. Ecol.* **29**, 804–813 (2018).

24. M. T. Irwin, J. L. Raharison, D. R. Raubenheimer, C. A. Chapman, J. M. Rothman, The nutritional geometry of resource scarcity: Effects of lean seasons and habitat disturbance on nutrient intakes and balancing in wild sifakas. *PLOS ONE* **10**, e0128046 (2015).

25. C. P. van Schaik, K. R. Pfannes, Tropical climates and phenology: A primate perspective, in *Seasonality in Primates: Studies of Living and Extinct Human and Non-Human Primates*, D. K. Brockman, C. P. van Schaik, Eds. (Cambridge Univ. Press, 2005), pp. 23–54.

26. A. E. Russon, S. A. Wich, M. Ancrenaz, T. Kanamori, C. D. Knott, N. Kuze, H. Morrogh-Bernard, P. Pratje, H. Ramlee, P. Rodman, Geographic variation in orangutan diets, in *Orangutans: Geographic Variation in Behavioral Ecology*, S. A. Wich, S. S. Utami Atmoko, T. Mitra Setia, C. P. van Schaik, Eds. (Oxford Univ. Press, 2008), pp. 135–156.

27. L. A. Shipley, J. S. Forbey, B. D. Moore, Revisiting the dietary niche: When is a mammalian herbivore a specialist? *Integr. Comp. Biol.* **49**, 274–290 (2009).

28. G. E. Machovsky-Capuska, A. M. Senior, S. J. Simpson, D. Raubenheimer, The multidimensional nutritional niche. *Trends Ecol. Evol.* **31**, 355–365 (2016).

29. M. Leighton, Modeling dietary selectivity by Bornean orangutans: Evidence for integration of multiple criteria in fruit selection. *Int. J. Primatol.* **14**, 257–313 (1993).

30. E. R. Vogel, S. E. Alavi, S. S. Utami-Atmoko, M. A. van Noordwijk, T. D. Bransford, W. M. Erb, A. Zulfa, F. Sulistyo, W. R. Farida, J. M. Rothman, Nutritional ecology of wild Bornean orangutans (*Pongo pygmaeus wurmbii*) in a peat swamp habitat: Effects of age, sex, and season. *Am. J. Primatol.* **79**, 1–20 (2017).

31. M. E. Harrison, H. C. Morrogh-Bernard, D. J. Chivers, Orangutan energetics and the influence of fruit availability in the nonmasting peat-swamp forest of Sabangau, Indonesian Borneo. *Int. J.Primatol.* **31**, 585–607 (2010).

32. C. D. Knott, Changes in orangutan caloric intake, energy balance, and ketones in response to fluctuating fruit availability. *Int. J.Primatol.* **19**, 1061–1079 (1998).

33. E. R. Vogel, M. E. Harrison, A. Zulfa, T. D. Bransford, S. E. Alavi, S. Husson, H. Morrogh-Bernard, Santiano, T. Firtsman, S. S. Utami-Atmoko, M. A. van Noordwijk, W. R. Farida, Nutritional differences between two orangutan habitats: Implications for population density. *PLOS ONE* **10**, e0138612 (2015).

34. M. Emery Thompson, C. D. Knott, Urinary C-peptide of insulin as a non-invasive marker of energy balance in wild orangutans. *Horm. Behav.* **53**, 526–535 (2008).

35. C. A. O'Connell, A. L. DiGiorgio, A. D. Ugarte, R. S. A. Brittain, D. J. Naumenko, S. S. U. Atmoko, E. R. Vogel, Wild Bornean orangutans experience muscle catabolism during episodes of fruit scarcity. *Sci. Rep.* **11**, 10185 (2021).

36. M. A. van Noordwijk, S. S. Utami Atmoko, C. D. Knott, N. Kuze, H. C. Morrogh-Bernard, F. Oram, C. Schuppli, C. P. van Schaik, E. P. Willems, The slow ape: High infant survival and long interbirth intervals in wild orangutans. *J. Hum. Evol.* **125**, 38–49 (2018).

37. H. Pontzer, D. A. Raichlen, R. W. Shumaker, C. Ocobock, S. A. Wich, Metabolic adaptation for low energy throughput in orangutans. *Proc. Natl. Acad. Sci. U.S.A.* **107**, 14048–14052 (2010).

38. Y. Narita, S.-i. Oda, O. Takensaka, T. Kageyama, Lineage-specific duplication and loss of pepsinogen genes in hominoid evolution. *J. Mol. Evol.* **70**, 313–324 (2010).

39. E. R. Vogel, J. T. van Woerden, P. W. Lucas, S. S. U. Atmoko, C. P. van Schaik, N. J. Dominy, Functional ecology and evolution of hominoid molar enamel thickness: *Pan troglodytes schweinfurthii* and *Pongo pygmaeus wurmbii*. *J. Hum. Evol.* **55**, 60–74 (2008).

40. A. B. Taylor, E. R. Vogel, N. J. Dominy, Food material properties and mandibular load resistance abilities in large-bodied hominoids. *J. Hum. Evol.* **55**, 604–616 (2008).

41. M. P. Mattle-Greminger, T. Bilgin Sonay, A. Nater, M. Pybus, T. Desai, G. de Valles, F. Casals, A. Scally, J. Bertranpetit, T. Marques-Bonet, C. P. van Schaik, M. Anisimova, M. Krützen, Genomes reveal marked differences in the adaptive evolution between orangutan species. *Genome Biol.* **19**, 193 (2018).

42. S. S. Utami, S. A. Wich, E. H. M. Sterck, J. van Hooff, Food competition between wild orangutans in large fig trees. *Int. J. Primatol.* **18**, 909–927 (1997).

43. C. P. van Schaik, The socioecology of fission-fusion sociality in orangutans. *Primates* **40**, 69–86 (1999).

44. J. A. Kunz, G. J. Duvot, M. A. van Noordwijk, E. P. Willems, M. Townsend, N. Mardianah, S. S. Utami Atmoko, E. R. Vogel, T. P. Nugraha, M. Heistermann, M. Agil, T. Weingrill, C. P. van Schaik, The cost of associating with males for Bornean and Sumatran female orangutans: A hidden form of sexual conflict? *Behav. Ecol. Sociobiol.* **75**, 6 (2021).

45. G. F. Cahill Jr., Fuel metabolism in starvation. *Annu. Rev. Nutr.* **26**, 1–22 (2006).

46. M. Watford, Starvation: Metabolic changes. *eLS* 1–7 (2015).

47. D. S. Sherry, P. T. Ellison, Potential applications of urinary C-peptide of insulin for comparative energetics research. *Am. J. Phys. Anthropol.* **133**, 771–778 (2007).

48. M. Emery Thompson, Energetics of feeding, social behavior, and life history in non-human primates. *Horm. Behav.* **91**, 84–96 (2017).

49. M. Emery Thompson, in *The International Encyclopedia of Primatology*, A. Fuentes, Ed. (Wiley-Blackwell Press, 2017), vol. 1, pp. 1–2.

50. M. Emery Thompson, M. N. Muller, R. W. Wrangham, J. S. Lwanga, K. B. Potts, Urinary C-peptide tracks seasonal and individual variation in energy balance in wild chimpanzees. *Horm. Behav.* **55**, 299–305 (2009).

51. M. L. Bergstrom, U. Kalbitzer, F. A. Campos, A. D. Melin, M. Emery Thompson, L. M. Fedigan, Non-invasive estimation of the costs of feeding competition in a neotropical primate. *Horm. Behav.* **118**, 104632 (2020).

52. T. Deschner, J. Kratzsch, G. Hohmann, Urinary C-peptide as a method for monitoring body mass changes in captive bonobos (*Pan paniscus*). *Horm. Behav.* **54**, 620–626 (2008).

53. P. D. Valé, J.-C. K. Béné, A. K. N'Guessan, C. Crockford, T. Deschner, I. Koné, C. Girard-Buttoz, R. M. Wittig, Energetic management in wild chimpanzees (*Pan troglodytes verus*) in Taï National Park, Côte d'Ivoire. *Behav. Ecol. Sociobiol.* **75**, 1 (2021).

54. Y. T. Kruszynska, P. D. Home, I. Hanning, K. G. Alberti, Basal and 24-h C-peptide and insulin secretion rate in normal man. *Diabetologia* **30**, 16–21 (1987).

55. E. R. Vogel, C. D. Knott, B. E. Crowley, M. D. Blakely, M. D. Larsen, N. J. Dominy, Bornean orangutans on the brink of protein bankruptcy. *Biol. Lett.* **8**, 333–336 (2012).

56. P. S. Barboza, K. L. Parker, Body protein stores and isotopic indicators of N balance in female reindeer (*Rangifer tarandus*) during winter. *Physiol. Biochem. Zool.* **79**, 628–644 (2006).

57. K. N. Frayn, R. D. Evans, *Human Metabolism: A Regulatory Perspective, 4th Edition* (Wiley, 2019).

58. D. Raubenheimer, S. J. Simpson, Nutrient balancing in grasshoppers: Behavioural and physiological correlates of dietary breadth. *J. Exp. Biol.* **206**, 1669–1681 (2003).

59. M. Watford, in *Encyclopedia of Biochemistry* (Elsevier, ed. 3, 2020).

60. D. Raubenheimer, S. J. Simpson, Protein leverage: Theoretical foundations and ten points of clarification. *Obesity (Silver Spring)* **27**, 1225–1238 (2019).

61. K. D. Hall, The potential role of protein leverage in the US obesity epidemic. *Obesity* **27**, 1222–1224 (2019).

62. S. J. Simpson, D. Raubenheimer, Obesity: The protein leverage hypothesis. *Obes. Rev.* **6**, 133–142 (2005).

63. J. E. Lambert, J. M. Rothman, Fallback foods, optimal diets, and nutritional targets: Primate responses to varying food availability and quality. *Ann. Rev. Anthropol.* **44**, 493–512 (2015).

64. Food and Nutrition Board of the Institute of Medicine of the National Academies, in *Food and Nutrition Board of the Institute of Medicine of the National Academies* (National Academies Press, 2005).

65. S. E. Alavi, "Diet, cognition, and nutrient balancing in an orangutan habitat," thesis, The State University of New Jersey, Rutgers (2018).

66. A. L. DiGiorgio, E. M. Upton, T. W. Susanto, C. D. Knott, Wild Bornean orangutan (*Pongo pygmaeus wurmbii)* feeding rates and the Marginal Value Theorem. *Am. J. Primatol.* **82**, e23183 (2020).

67. C. P. Campbell, D. Raubenheimer, A. V. Badaloo, P. D. Gluckman, C. Martinez, A. Gosby, S. J. Simpson, C. Osmond, M. S. Boyne, T. E. Forrester, Developmental contributions to macronutrient selection: A randomized controlled trial in adult survivors of malnutrition. *Evol. Med. Public Heallth* **2016**, 158–169 (2016).

68. D. J. Naumenko, M. Watford, S. S. Utami Atmoko, W. M. Erb, E. R. Vogel, Evaluating ketosis in primate field studies: Validation of urine test strips in wild Bornean orangutans (*Pongo pygmaeus wurmbii*). *Folia Primatol.* **91**, 159–168 (2019).

69. O. E. Owen, P. Felig, A. P. Morgan, J. Wahren, G. F. Cahill Jr., Liver and kidney metabolism during prolonged starvation. *J. Clin. Invest.* **48**, 574–583 (1969).

70. A. A. Gibson, E. I. Eroglu, K. Rooney, C. Harper, S. McClintock, J. Franklin, T. P. Markovic, R. V. Seimon, A. Sainsbury, Urine dipsticks are not accurate for detecting mild ketosis during a severely energy restricted diet. *Obes. Sci. Pract.* **6**, 544–551 (2020).

71. R. W. Wrangham, N. L. Conklin-Brittain, K. D. Hunt, Dietary response of chimpanzees and cercopithecines to seasonal variation in fruit abundance. I. Antifeedants. *Int. J. Primatol.* **19**, 949–970 (1998).

72. C. C. Grueter, T. Deschner, V. Behringer, K. Fawcett, M. M. Robbins, Socioecological correlates of energy balance using urinary C-peptide measurements in wild female mountain gorillas. *Physiol. Behav.* **127**, 13–19 (2014).

73. Y. Nie, F. Wei, W. Zhou, Y. Hu, A. M. Senior, Q. Wu, L. Yan, D. Raubenheimer, Giant pandas are macronutritional carnivores. *Curr. Biol.* **29**, 1677–1682.e2 (2019).

74. I. Fürtbauer, C. Christensen, A. Bracken, M. J. O'Riain, M. Heistermann, A. J. King, Energetics at the urban edge: Environmental and individual predictors of urinary C-peptide levels in wild chacma baboons (*Papio ursinus*). *Horm. Behav.* **126**, 104846 (2020).

75. A. M. Ashbury, J. Meric de Bellefon, J. A. Kunz, M. Abdullah, A. M. Marzec, C. Fryns, S. S. Utami Atmoko, C. P. van Schaik, M. A. van Noordwijk, After the smoke has cleared: Extended low fruit productivity following forest fires decreased gregariousness and social tolerance among wild female Bornean orangutans (*Pongo pygmaeus wurmbii*). *Int. J. Primatol.* **43**, 189–215 (2022).

76. A. Rietman, J. Schwarz, D. Tomé, F. J. Kok, M. Mensink, High dietary protein intake, reducing or eliciting insulin resistance? *Eur. J. Clin. Nutr.* **68**, 973–979 (2014).

77. R. L. Jungas, M. L. Halperin, J. T. Brosnan, Quantitative analysis of amino acid oxidation and related gluconeogenesis in humans. *Physiol. Rev.* **72**, 419–448 (1992).

78. V. M. Oelze, J. S. Head, M. M. Robbins, M. Richards, C. Boesch, Niche differentiation and dietary seasonality among sympatric gorillas and chimpanzees in Loango National Park (Gabon) revealed by stable isotope analysis. *J. Hum. Evol.* **66**, 95–106 (2014).

79. E. G. Wessling, V. M. Oelze, H. Eshuis, J. D. Pruetz, H. S. Kuhl, Stable isotope variation in savanna chimpanzees (*Pan troglodytes verus*) indicate avoidance of energetic challenges through dietary compensation at the limits of the range. *Am. J. Phys. Anthropol.* **168**, 665–675 (2019).

80. L. Heck, B. E. Crowley, S. Thoren, U. Radespiel, Determinants of isotopic variation in two sympatric mouse lemur species from northwestern Madagascar, in *The Dwarf and Mouse Lemurs of Madagascar: Biology, Behavior and Conservation Biogeography of the Cheirogaleidae*, S. M. Lehman, U. Radespiel, Eds. (Cambridge Univ. Press, 2016), pp. 282–304.

81. T. Tsutaya, A. Wong, P. T. Malim, H. Bernard, N. O. Ogawa, N. Ohkouchi, S. Hongo, T. Tajima, T. Kanamori, N. Kuze, Stable isotopic investigation of the feeding ecology of wild Bornean orangutans. *Am. J. Biol. Anthropol.* **179**, 276–290 (2022).

82. C. D. Knott, B. Crowley, M. Brown, T. W. Susanto, Fecal isotopes as indicators of weaning and diet in wild Bornean orangutans. *Amer J. Phys. Anthrop.* **168**, 128–129 (2019).

83. C. J. Kettle, J. Ghazoul, P. S. Ashton, C. H. Cannon, L. Chong, B. Diway, E. Faridah, R. Harrison, A. Hector, P. Hollingsworth, L. P. Koh, E. Khoo, K. Kitayama, K. Kartawinata, A. J. Marshall, C. R. Maycock, S. Nanami, G. Paoli, M. D. Potts, D. Sheil, S. Tan, I. Tomoaki, C. Webb, T. Yamakura, D. F. R. P. Burslem, Mass fruiting in Borneo: A missed opportunity. *Science* **330**, 584–584 (2010).

84. C. H. Cannon, L. M. Curran, A. J. Marshall, M. Leighton, Beyond mast-fruiting events: Community asynchrony and individual dormancy dominate woody plant reproductive behavior across seven Bornean forest types. *Curr. Sci.* **93**, 1558–1566 (2007).

85. L. M. Curran, M. Leighton, Vertebrate responses to spatiotemporal variation in seed production of mast-fruiting Dipterocarpaceae. *Ecological monographs* **70**, 101–128 (2000).

86. S. A. Wich, C. P. van Schaik, The impact of El Niño on mast fruiting in Sumatra and elsewhere in Malesia. *J. Trop. Ecol.* **16**, 563–577 (2000).

87. C. H. Cannon, L. M. Curran, A. J. Marshall, M. Leighton, Long-term reproductive behaviour of woody plants across seven Bornean forest types in the Gunung Palung National Park (Indonesia): Suprannual synchrony, temporal productivity and fruiting diversity. *Ecol. Lett.* **10**, 956–969 (2007).

88. S. Kaboth-Bahr, W. D. Gosling, R. Vogelsang, A. Bahr, E. M. L. Scerri, A. Asrat, A. S. Cohen, W. Dusing, V. Foerster, H. F. Lamb, M. A. Maslin, H. M. Roberts, F. Schabitz, M. H. Trauth, Paleo-ENSO influence on African environments and early modern humans. *Proc. Natl. Acad. Sci. U.S.A.* **118**, e2018277118 (2021).

89. F. Luca, G. H. Perry, A. Di Rienzo, Evolutionary adaptations to dietary changes. *Annu. Rev. Nutr.* **30**, 291–314 (2010).

90. J. V. Neel, Diabetes mellitus: A "thrifty" genotype rendered detrimental by "progress"? *Am. J. Hum. Genet.* **14**, 353–362 (1962).

91. P. Carrera-Bastos, M. Fontes-Villalba, J. H. O'Keefe, The western diet and lifestyle and diseases of civilization. *Res. Rep. Clin. Cardiol.* **2**, 15–35 (2011).

92. L. Cordain, S. B. Eaton, A. Sebastian, N. Mann, S. Lindeberg, B. A. Watkins, J. H. O'Keefe, J. Brand-Miller, Origins and evolution of the Western diet: Health implications for the 21st century. *Am. J. Clin. Nutr.* **81**, 341–354 (2005).

93. R. Blekhman, A. Oshlack, A. E. Chabot, G. K. Smyth, Y. Gilad, Gene regulation in primates evolves under tissue-specific selection pressures. *PLOS Genet.* **4**, e1000271 (2008).

94. G. H. Perry, N. J. Dominy, K. G. Claw, A. S. Lee, H. Fiegler, R. Redon, J. Werner, F. A. Villanea, J. L. Mountain, R. Misra, N. P. Carter, C. Lee, A. C. Stone, Diet and the evolution of human amylase gene copy number variation. *Nat. Genet.* **39**, 1256–1260 (2007).

95. P. Pajic, P. Pavlidis, K. Dean, L. Neznanova, R. A. Romano, D. Garneau, E. Daugherity, A. Globig, S. Ruhl, O. Gokcumen, Independent amylase gene copy number bursts correlate with dietary preferences in mammals. *eLife* **8**, e44628 (2019).

96. P. J. D. Elder, D. B. Ramsden, D. Burnett, M. O. Weickert, T. M. Barber, Human amylase gene copy number variation as a determinant of metabolic state. *Expert Rev. Endocrinol. Metab.* **13**, 193–205 (2018).

97. C. M. K. Venkatapoorna, P. Ayine, E. P. Parra, T. Koenigs, M. Phillips, J. R. Babu, M. Sandey, T. Geetha, Association of salivary amylase (*AMY1*) gene copy number with obesity in Alabama elementary school children. *Nutrients* **11**, 1379 (2019).

98. C. K. Martin, S. K. Das, L. Lindblad, S. B. Racette, M. A. McCrory, E. P. Weiss, J. P. Delany, W. E. Kraus, CALERIE Study Team, Effect of calorie restriction on the free-living physical activity levels of nonobese humans: Results of three randomized trials. *J. Appl. Physiol.* **110**, 956–963 (2011).

99. P. Baker, P. Machado, T. Santos, K. Sievert, K. Backholer, M. Hadjikakou, C. Russell, O. Huse, C. Bell, G. Scrinis, A. Worsley, S. Friel, M. Lawrence, Ultra-processed foods and the nutrition transition: Global, regional and national trends, food systems transformations and political economy drivers. *Obes. Rev.* **21**, e13126 (2020).

100. A. Grech, Z. Sui, A. Rangan, S. J. Simpson, S. C. P. Coogan, D. Raubenheimer, Macronutrient (im)balance drives energy intake in an obesogenic food environment: An ecological analysis. *Obesity (Silver Spring)* **30**, 2156–2166 (2022).

101. K. D. Hall, A. Ayuketah, R. Brychta, H. Y. Cai, T. Cassimatis, K. Y. Chen, S. T. Chung, E. Costa, A. Courville, V. Darcey, L. A. Fletcher, C. G. Forde, A. M. Gharib, J. Guo, R. Howard, P. V. Joseph, S. McGehee, R. Ouwerkerk, K. Raisinger, I. Rozga, M. Stagliano, M. Walter, P. J. Walter, S. Yang, M. G. Zhou, Ultra-processed diets cause excess calorie intake and weight gain: An inpatient randomized controlled trial of ad libitum food intake. *Cell Metab.* **30**, 226–226 (2019).

102. S. Hamano, M. Sawada, M. Aihara, Y. Sakurai, R. Sekine, S. Usami, N. Kubota, T. Yamauchi, Ultra-processed foods cause weight gain and increased energy intake associated with reduced chewing frequency: A randomized, open-label, crossover study. *Diabetes Obes. Metab.* **26**, 5431–5443 (2024).

103. T. A. Gresl, S. T. Baum, J. W. Kemnitz, Glucose regulation in captive *Pongo pygmaeus abelii*, *P-p. pygmaeus*, and *P-p. abelii* x *P-p. pygmaeus* orangutans. *Zoo Biol.* **19**, 193–208 (2000).

104. A. L. Zihlman, R. K. McFarland, C. E. Underwood, Functional anatomy and adaptation of male gorillas (*Gorilla gorilla gorilla*) with comparison to male orangutans (*Pongo pygmaeus*). *Anat. Rec.* **294**, 1842–1855 (2011).

105. R. C. Miller, E. Brindle, D. J. Holman, J. Shofer, N. A. Klein, M. R. Soules, K. A. O'Connor, Comparison of specific gravity and creatinine for normalizing urinary reproductive hormone concentrations. *Clin. Chem.* **50**, 924–932 (2004).

106. B. E. Crowley, Stable isotope techniques and applications for primatologists. *Int. J. Primatol.* **33**, 673–701 (2012).

107. G. Skrzypek, Normalization procedures and reference material selection in stable HCNOS isotope analyses: An overview. *Anal. Bioanal. Chem.* **405**, 2815–2823 (2013).

108. P.-C. Bürkner, Advanced Bayesian multilevel modeling with the R package brms. *R J.* **10**, 395–411 (2018).

109. A. Baez-Ortega, in In silico: *Naïve Thoughts on Data* (2018), vol. 2022; https://baezortega.github.io

110. A. S. Kurz, in Bayesian robust correlations with brms (and why you should love Student's `t`), *Blog* (2019), vol. 2022; https://solomonkurz.netlify.app/blog/2019-02-10-bayesian-robust-correlations-with-brms-and-why-you-should-love-student-s-t/.

111. D. J. Barr, R. Levy, C. Scheepers, H. J. Tily, Random effects structure for confirmatory hypothesis testing: Keep it maximal. *J. Mem. Lang.* **68**, 255–278 (2013).

112. C. J. Curtis, G. L. Simpson, Trends in bulk deposition of acidity in the UK, 1988–2007, assessed using additive models. *Ecol. Indic.* **37**, 274–286 (2014).

113. D. T. Monteith, C. D. Evans, P. A. Henrys, G. L. Simpson, I. A. Malcolm, Trends in the hydrochemistry of acid-sensitive surface waters in the UK 1988–2008. *Ecol. Indic.* **37**, 287–303 (2014).

114. Z. Goldsborough, M. C. Crofoot, S. E. Alavi, E. Del Rosario-Vargas, S. F. Garza, K. Tiedeman, B. J. Barrett, Coupling of coastal activity with tidal cycles is stronger in tool-using capuchins (*Cebus capucinus imitator*). *R. Soc. Open Sci.* **10**, 230355 (2023).

115. S. E. Alavi, sealavi/GAM-first-derivative-functions: v1.0 (v1.0) (Zenodo, 2022).

116. S. E. Alavi, Integrated behavioral and metabolically flexible responses of wild orangutans to dietary variation v1 (v1.0) (Zenodo, 2024); https://doi.org/10.5281/zenodo.10451962.

117. I. o. Medicine, *Dietary Reference Intakes for Energy, Carbohydrate, Fiber, Fat, Fatty Acids, Cholesterol, Protein, and Amino Acids* (The National Academies Press, 2005), pp. 1358.

118. N. L. Conklin-Brittain, E. S. Dierenfeld, R. W. Wrangham, M. Norconk, S. C. Silver, Chemical protein analysis: A comparison of Kjeldahl crude protein and total ninhydrin protein from wild, tropical vegetation. *J. Chem. Ecol.* **25**, 2601–2622 (1999).

119. N. L. Conklin-Brittain, C. D. Knott, R. W. Wrangham, in *Feeding Ecology in Apes and Other Primates: Ecological, Physiological and Behavioural Aspects*, G. Hohmann, M. Robbins, C. Boesch, Eds. (Cambridge Studies in Biological and Evolutionary Anthropology, Cambridge Univ. Press, 2006), pp. 445–471.

120. A. A. Kolodziejczyk, D. Zheng, E. Elinav, Diet-microbiota interactions and personalized nutrition. *Nat. Rev. Microbiol.* **17**, 742–753 (2019).

121. N. Reichardt, M. Vollmer, G. Holtrop, F. M. Farquharson, D. Wefers, M. Bunzel, S. H. Duncan, J. E. Drew, L. M. Williams, G. Milligan, T. Preston, D. Morrison, H. J. Flint, P. Louis, Specific substrate-driven changes in human faecal microbiota composition contrast with functional redundancy in short-chain fatty acid production. *ISME J.* **12**, 610–622 (2018).

122. F. Fava, L. Rizzetto, K. M. Tuohy, Gut microbiota and health: Connecting actors across the metabolic system. *Proc. Nutr. Soc.* **78**, 177–188 (2018).

123. R. V. Barbehenn, C. Peter Constabel, Tannins in plant-herbivore interactions. *Phytochemistry* **72**, 1551–1565 (2011).

124. K. D. Evans, W. J. Foley, C. A. Chapman, J. M. Rothman, Deconstructing protein in the diet and biomass of Colobine primates. *Int. J. Primatol.* **42**, 283–300 (2021).

125. T. J. Hastie, R. Tibshirani, Generalized additive models. *Stat. Sci.* **1**, 297–310 (1986).

126. S. N. Wood, Low-rank scale-invariant tensor product smooths for generalized additive mixed models. *Biometrics* **62**, 1025–1036 (2006).
